# Nickel-catalyzed transamidation of aliphatic amide derivatives†
†Electronic supplementary information (ESI) available. See DOI: 10.1039/c7sc01980g



**DOI:** 10.1039/c7sc01980g

**Published:** 2017-07-10

**Authors:** Jacob E. Dander, Emma L. Baker, Neil K. Garg

**Affiliations:** a Department of Chemistry and Biochemistry , University of California , 607 Charles Young Drive East, Box 951569 , Los Angeles , CA 90095 , USA . Email: neilgarg@chem.ucla.edu

## Abstract

Transamidation, or the conversion of one amide to another, is a long-standing challenge in organic synthesis. Although notable progress has been made in the transamidation of primary amides, the transamidation of secondary amides has remained underdeveloped, especially when considering aliphatic substrates. Herein, we report a two-step approach to achieve the transamidation of secondary aliphatic amides, which relies on non-precious metal catalysis. The method involves initial Boc-functionalization of secondary amide substrates to weaken the amide C–N bond. Subsequent treatment with a nickel catalyst, in the presence of an appropriate amine coupling partner, then delivers the net transamidated products. The transformation proceeds in synthetically useful yields across a range of substrates. A series of competition experiments delineate selectivity patterns that should influence future synthetic design. Moreover, the transamidation of Boc-activated secondary amide derivatives bearing epimerizable stereocenters underscores the mildness and synthetic utility of this methodology. This study provides the most general solution to the classic problem of secondary amide transamidation reported to date.

## Abstract 2

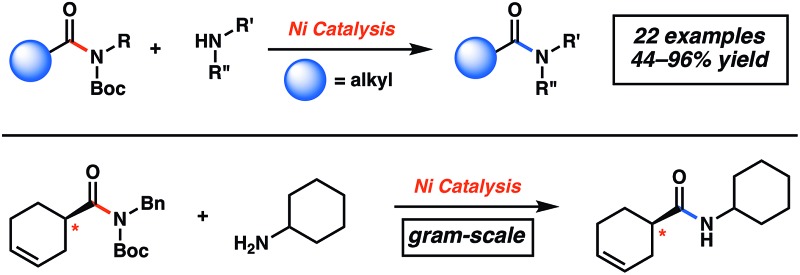
We report a two-step approach to achieve the transamidation of secondary aliphatic amides using non-precious metal catalysis.

## Introduction

**Fig. 1:**
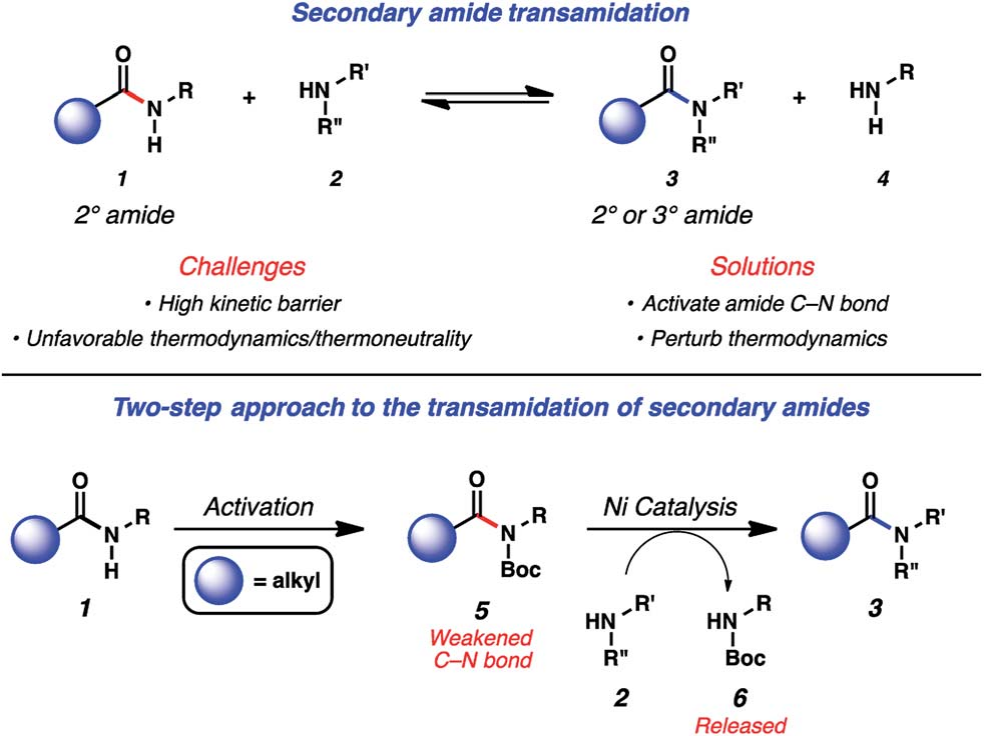
Challenges associated with secondary amide transamidation and the two step-approach to realize this challenging synthetic transformation.

The ability to convert one amide to another, known as the transamidation reaction, represents a long-standing synthetic challenge.^1,2^ Although significant progress has been made with regard to the transamidation of 1° amides,^3^ the corresponding reaction involving secondary amides (**1** + **2** → **3** + **4**) has remained largely underdeveloped (Fig. 1). Two factors are primarily responsible for the difficulty of this transformation. First, the kinetic barrier to break the amide C–N bond is considered high because of well-known resonance effects.^4^ The second complication stems from thermodynamics, as the energetics of starting materials and products in transamidation reactions are often comparable, resulting in thermoneutral rections.^1a,5^

Despite these challenges, several breakthroughs have been reported with regard to secondary amide transamidation. Gellman and Stahl utilized a dimeric aluminum complex to affect secondary amide transamidation, albeit with equilibrium mixtures resulting, thus highlighting the difficulty regarding thermodynamics.^6^ Bertrand has reported a means to achieve secondary amide transamidation of simple substrates using excess AlCl_3_.^7^ In both of these cases, the transamidation is made possible by Lewis acid activation of the amide carbonyl. Most recently, Szostak reported two simple protocols for achieving the transamidation of secondary amide derivatives, each with a focus on benzamide-derived substrates. The first uses Lewis base catalysis,^8^ while the other utilizes Pd–NHC complexes.^9^ Despite these discoveries, a general solution to the transamidation of aliphatic 2° amides has remained elusive.

In considering the challenges noted earlier, we sought to develop an alternative strategy to achieve the transamidation of secondary amides. As summarized in Fig. 1, it was envisioned that N-functionalization of secondary amide substrates **1** could lead to weakening of the acyl amide C–N bond,^10^ if the appropriate activating group was utilized. Electron-withdrawing groups, such as the Boc group, were viewed as ideal for this purpose,^11^ given the ease by which Boc groups can be introduced. From the resulting Boc-activated secondary amide **5**, it was believed that oxidative addition with an appropriate nickel catalyst could occur through a reasonable kinetic process. *In situ* interception of this species with an amine nucleophile **2** would furnish transamidated product **3**. The process would be driven thermodynamically by the favorable release of carbamate **6**.^12^

Encouraged by the successful activation of amide C–N bonds using transition metal catalysis, as demonstrated by studies from Szostak, Shi, and our laboratory,^13–17^ we explored the sequence described above. In 2016, we validated this approach to achieve a two-step transamidation of *N*-Bn,Boc benzamide derivatives.^18^ However, the corresponding reaction sequence using substrates derived from aliphatic secondary amides was unsuccessful. In this manuscript, we describe our efforts to overcome this hurdle, which have led to the nickel-catalyzed transamidation of aliphatic amide derivatives. The methodology presented herein offers a robust solution to the classic problem of secondary aliphatic amide transamidation, and is expected to inform future efforts toward natural product synthesis and derivatization.

## Results and discussion

### Reaction discovery and optimization

To initiate our studies, we selected imide **7**, obtained by Boc-activation of the corresponding aliphatic amide, and cyclohexylamine (**8**) as the reaction partners (Table 1). As both coupling partners possess α-branching and are sterically hindered, they were considered excellent challenges for methodology development. We first tested the amidation using 10 mol% Ni(cod)_2_ and 20 mol% SIPr (**10**), to parallel the conditions we employed in our original disclosure involving benzamide substrates (entry 1).^18^ As anticipated, only a low yield of amide **9** was obtained. We also tested terpyridine (**11**) as the ligand (entry 2), as this was shown to be effective in promoting the Ni-catalyzed esterification of aliphatic amide derivatives.^14e^ To our surprise, no reaction occurred, which prompted us to evaluate additional NHC ligands. We were delighted to find that use of ligand precursor **12**, in combination with NaO*t*Bu for *in situ* free-basing, afforded the desired amide product **9** in quantitative yield (entry 3). We attribute the improved competency of **12** (compared to **10**) to its more electron-rich nature, which ultimately renders oxidative addition more facile.^19,20^ Of note, the reaction also took place using lower amounts of catalyst, ligand, and base (entry 4). Finally, we found that by increasing the concentration, the equivalents of amine could be reduced from 2.0 to 1.5, while also allowing for shorter reaction times (entry 5). These conditions (entry 5) were found to be sufficiently general and were used to explore the reaction scope. Of note, in the absence of Ni(cod)_2_, no reaction occurs, thus demonstrating that the conversion of **7** to **9** is indeed catalyzed by nickel.^21^ Likewise, Lewis base-promoted transamidation conditions were also deemed ineffective.^22^

**Table 1:** Optimization of the reaction conditions

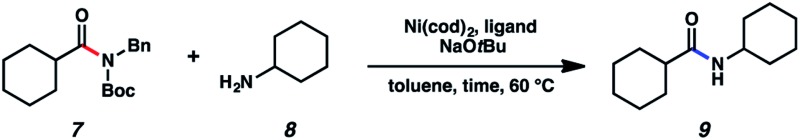							
Entry	Mol% of Ni(cod)_2_	Ligand (mol%)	Mol% of NaO*t*Bu	Equiv. of **8**	Conc.	Time	Yield of **9**^*a*^
1	10	**10** (20 mol%)	—	2.0	1.0 M	24 h	15%
2	10	**11** (20 mol%)	—	2.0	1.0 M	24 h	0%
3	10	**12** (20 mol%)	22	2.0	1.0 M	24 h	100%
4	5	**12** (10 mol%)	11	2.0	1.0 M	24 h	100%
5	5	**12** (10 mol%)	11	1.5	1.5 M	18 h	90%
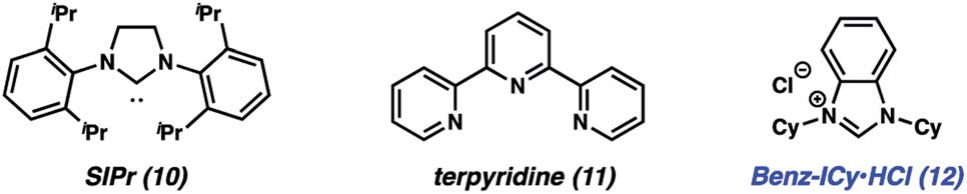							

^*a*^Yields determined by ^1^H NMR analysis using 1,3,5-trimethoxybenzene as an internal standard.

### Scope of methodology

**Fig. 2:**
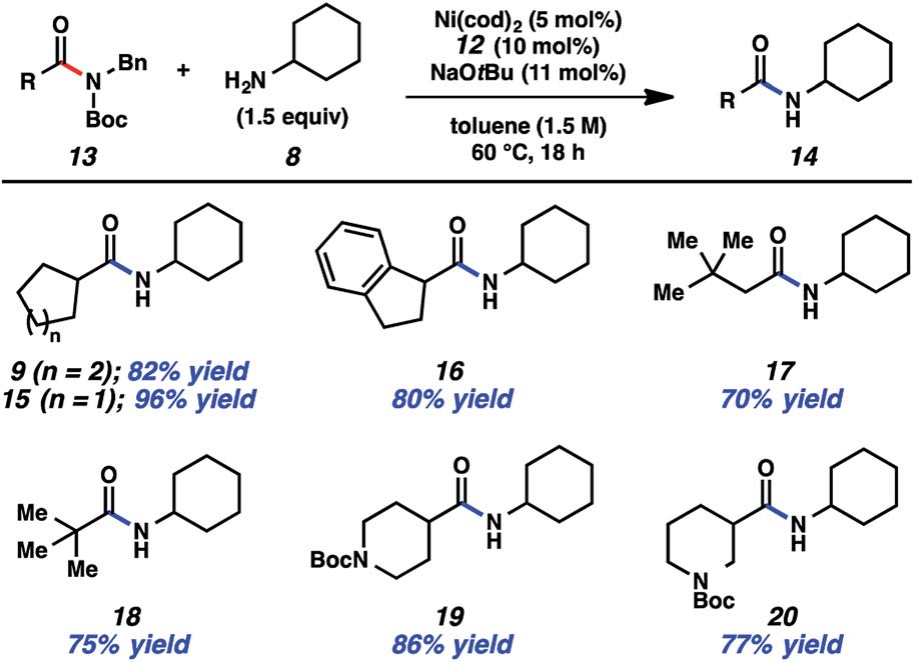
Variation of the amide substrate. Yields shown reflect the average of two isolation experiments.

Having arrived at suitable reaction conditions to achieve the transamidation of aliphatic amide derivative **7**, we explored the generality of our methodology by first varying the aliphatic amide partner (Fig. 2). Using cyclohexylamine (**8**), a variety of amide derivatives underwent the desired transamidation reaction. Beginning with the parent example, the desired amide product **9** was obtained in 82% isolated yield. Similarly, the corresponding cyclopentyl substrate could be utilized in the transamidation reaction to furnish **15** in excellent yield. As a further test of the methodology, an indane substrate was evaluated and found to undergo smooth coupling to give amide **16**. Additionally, two substrates bearing sterically encumbered *t*-butyl groups were tested. When the *t*-butyl group was positioned on the alpha carbon, neopentylic amide **17** was obtained in 70% yield. Direct linkage of the *t*-butyl group to the amide carbonyl carbon also did not hinder the reaction, as judged by the formation of pivalamide **18**. Lastly, we evaluated two piperidine-containing substrates, given the prevalence and importance of piperidines in medicinal chemistry.^23,24^ In both cases, the desired secondary amides were obtained in synthetically useful yields, as shown by the formation of **19** and **20**.

**Fig. 3:**
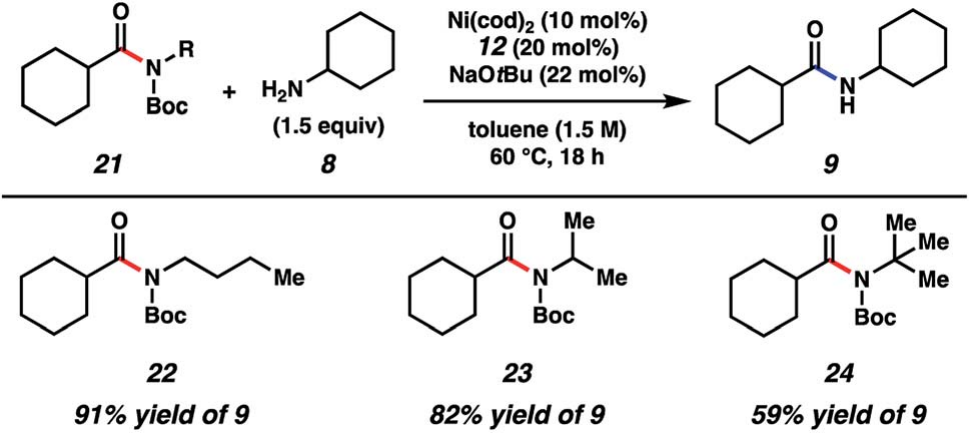
Scope of the amide substrate *N*-substituent. Yields shown reflect the average of two isolation experiments.

As shown in Fig. 3, the scope of this methodology was not limited to *N*-benzylamide derivatives. For example, *N-n*-Bu-containing substrate **22** could be employed, thus demonstrating that the aromatic benzyl substituent was not critical for success. Additionally, α-branching was tolerated, as judged by the successful coupling of the isopropylamine-derived substrate **23**. The methodology also displayed notable tolerance to sterics, given that *t*-butylamine substrate **24** could be coupled with cyclohexylamine (**8**) to furnish **9** in 59% yield.

**Fig. 4:**
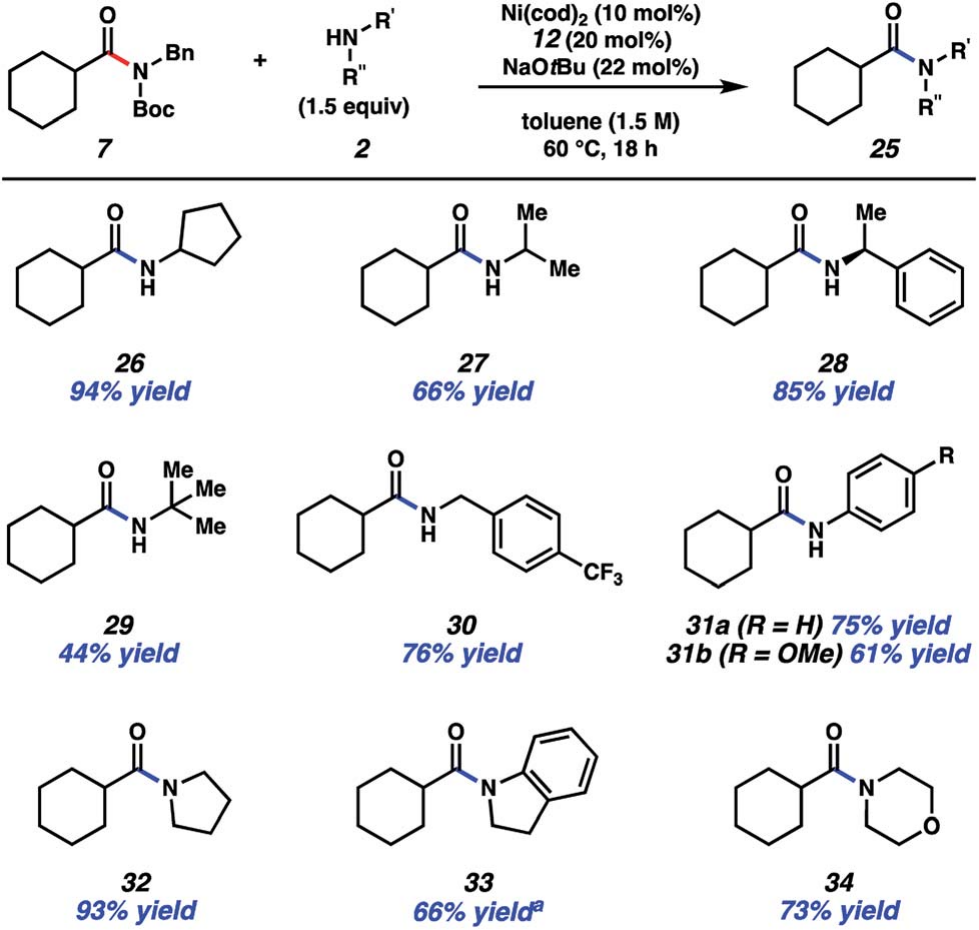
Scope of the amine nucleophile. Yields shown reflect the average of two isolation experiments. ^a^Yield determined by ^1^H NMR analysis using 1,3,5-trimethoxybenzene as an internal standard.

The scope of this methodology with respect to the amine nucleophile was also evaluated (Fig. 4). Several α-branched primary amines were tested, such as cyclopentyl amine, iso-propylamine, and *sec*-phenethylamine. These experiments led to the desired amides, **26–28**, respectively, in good to excellent yields. Even *t*-butylamine, which bears considerable steric hindrance, could be coupled as shown by the formation of **29**, albeit in somewhat diminished yield. *p*-Trifluoromethylbenzylamine was also utilized and gave rise to amide **30** in 76% yield. This result showcases an unbranched primary amine nucleophile, while also incorporating the medicinally relevant –CF_3_ group.^25^ The formation of **31a** and **31b** demonstrate that aniline and aniline derivatives can be utilized in this methodology. It should also be emphasized that secondary amines can be employed in the transamidation reaction to give tertiary amide products. The formation of **32–34**, which relied on the use of pyrrolidine, indoline, and morpholine, respectively, are representative of this notion.

### Amine competition studies

**Fig. 5:**
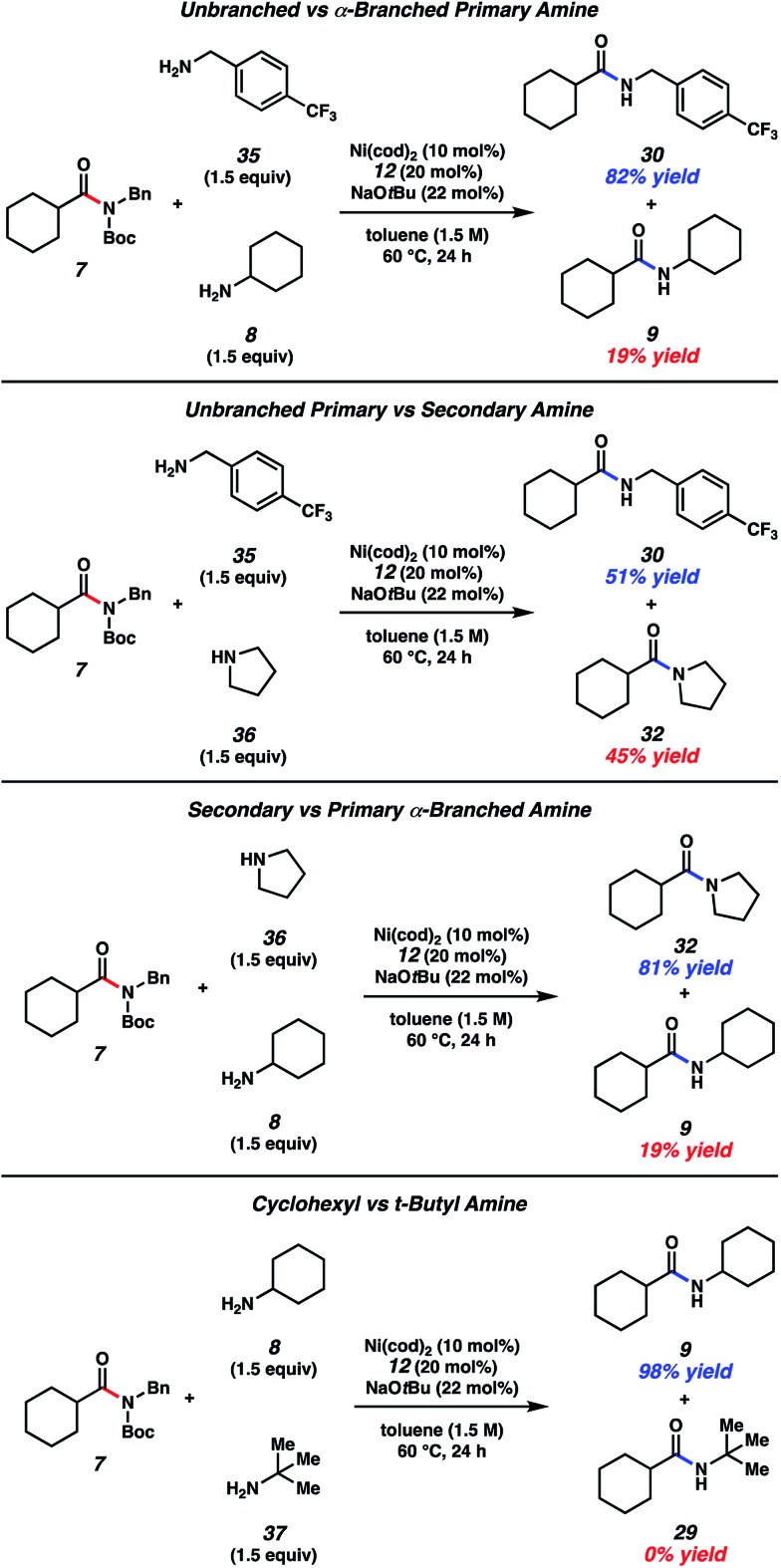
A series of amine competition experiments. Yields determined by ^1^H NMR analysis using hexamethylbenzene as an internal standard.

With the aim of identifying selectivity patterns that may aid in synthetic design, a series of competition experiments were performed using substrate **7** and various amine nucleophiles (Fig. 5). First, we compared *p*-trifluoromethylbenzylamine (**35**) and cyclohexylamine (**8**). The major product obtained was benzylamide **30** in 82% yield, with **9** being formed as the minor product. We attribute this selectivity to steric factors. Next, we compared *p*-trifluoromethylbenzylamine (**35**) and pyrrolidine (**36**). This reaction gave nearly a 1 : 1 ratio of products **30** and **32**, suggesting a fine balance between steric and electronic factors about the nucleophilic nitrogen in this case. In another comparison, pyrrolidine (**36**) and cyclohexylamine (**8**) were treated with amide **7**. Pyrrolidine-derived tertiary amide **32** was formed as the major product, rather than secondary amide **9**, consistent with the relative nucleophilicity of the amines being utilized.^26^ Lastly, we performed a competition experiment between cyclohexylamine (**8**) and *t*-butylamine (**37**), which led to the exclusive formation of cyclohexylamide **9**, presumably as a result of steric factors.

**Fig. 6:**
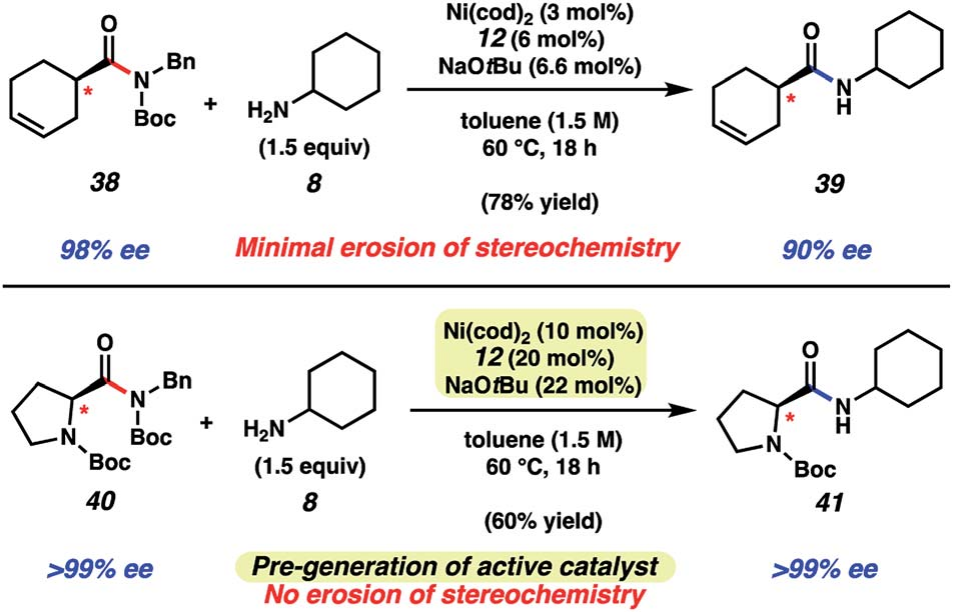
Transamidation of enantioenriched amide substrate **38** on gram-scale and transamidation of enantioenriched *N*-Boc proline substrate **40** using a modified protocol.

As a final test of our methodology, we evaluated two substrates that each bear an epimerizable stereocenter (Fig. 6). Treatment of cyclohexenamide **38** with cyclohexylamine (**8**) under typical reaction conditions, notably using only 3 mol% Ni(cod)_2_, delivered amide **39** in 78% yield on gram-scale. Of note, product **39** was obtained in 90% ee, indicative of minimal racemization occurring. Attempts to couple proline-derived amide **40** using our standard reaction protocol, on the other hand, led to substantial epimerization. We attribute this to the increased acidity of the substrate's α-proton relative to **38**. As a workaround, we developed a modified protocol that involves free-basing of ligand **12** in the presence of Ni(cod)_2_ in toluene to access the active catalyst in solution. Addition of the catalyst solution and amine **8** to substrate **40** afforded amide **41** after 18 h at 60 °C. Amide **41** was obtained in 60% yield and high optical purity. The mild and scalable nature of the reaction conditions bodes well for future synthetic applications.

## Conclusions

We have developed a facile approach to achieve the transamidation of secondary aliphatic amides, an unmet challenge in organic synthesis. Our strategy involves first preparing Boc-activated secondary amide derivatives and subsequently treating them with appropriate amine coupling partners under Ni-mediated reaction conditions. The methodology delivers secondary and tertiary amide products in synthetically useful yields across a range of substrates and amine nucleophiles. A variety of competition experiments were undertaken to reveal selectivity patterns, the results of which are expected to influence future synthetic design. Moreover, the transamidation of N-functionalized secondary amide derivatives bearing epimerizable stereocenters highlights the mildness and synthetic utility of this transformation. This study addresses the long-standing problem of secondary amide transamidation through the use of a general and mild nickel catalysis platform.
