# Supplementary material for: Nickel-catalyzed transamidation of aliphatic amide derivatives
Source: Chem Sci. 2017 Jul 10;8(9):6433–8. doi: 10.1039/c7sc01980g (PMC5632791; doi:10.1039/c7sc01980g)
Supplement: Supplementary file 1 [file SC-008-C7SC01980G-s001.pdf]

Electronic Supplementary Material (ESI) for Chemical Science.
This journal is © The Royal Society of Chemistry 2017

Electronic Supplementary Information – Table of Contents

Materials and Methods **S2**

Experimental Procedures **S3**
- **A. Syntheses of Starting Materials** **S3**
- **B. Reaction Discovery, Optimization, and Relevant Control Experiments** **S12**
- **C. Scope of Amide Substrates** **S14**
- **D. Scope of Amine Nucleophiles** **S18**
- **E. Amine Nucleophile Competition Experiments** **S23**
- **F. Gram Scale Transamidation of Enantioenriched Aliphatic Amide 38** **S26**
- **G. Procedure for the Transamidation of 40 using Pre-Formed Catalyst Solution** **S27**
- **H. Verification of Enantiopurity** **S28**
- **I. Lewis Base-Catalyzed Transamidation Control Experiments** **S36**

References **S41**

^{1}H NMR Spectra **S42**

^{13}C NMR Spectra… **S73**

Materials and Methods

Unless stated otherwise, reactions were conducted in flame-dried glassware under an atmosphere of nitrogen and commercially obtained reagents were used as received. Reagent **SI-5** was purchased from Sigma-Aldrich. Reagents **SI-3**, **SI-7**, **SI-9**, **SI-18** and **rac-SI-18** were purchased from Combi-Blocks, Inc. Amines **SI-11**, **SI-15**, **SI-19**, **SI-20**, **SI-21**, **SI-22**, **SI-23**, **8**, **35**, **36** and **37** were purchased from Sigma-Aldrich. Amine **SI-24** was purchased from Spectrum Chemical. Benzylamine was purchased from Sigma-Aldrich. Amines were passed through a plug of basic Alumina, distilled over CaH_2, and stored in an inert atmosphere glovebox prior to use. EDC and HOBt were purchased from Chem-Impex International. DMAP and Boc_2O were purchased from Oakwood Products, Inc. LiHMDS was purchased from Sigma-Aldrich and stored in an inert atmosphere glovebox. **SI-13** was obtained from Sigma-Aldrich and distilled over CaH_2 prior to use. Toluene was purified by distillation and taken through five freeze-pump-thaw cycles prior to use. CH_2Cl_2, NEt_3, THF, DMF, and CH_3CN were pass through activated alumina columns before use. $Ni(cod)_2$, 1,3-dicyclohexylbenzimidazolium chloride (Benz-ICy•HCl) and 1,3-Bis(2,6-di-*i*-propylphenyl)-4,5-dihydroimidazol-2-ylidine (SIPr) were obtained from Strem Chemicals. Terpyridine and 1,3-dicyclohexylimidazolium chloride (ICy•HCl) were obtained from Sigma Aldrich. 1,3,5-trimethoxybenzene (TMB), hexamethylbenzene (HMB) and NaO*t*Bu were obtained from Alfa Aesar. Reaction temperatures were controlled using an IKAmag temperature modulator, and unless stated otherwise, reactions were performed at room temperature (approximately 23 °C). Thin-layer chromatography (TLC) was conducted with EMD gel 60 F254 pre-coated plates (0.25 mm for analytical chromatography and 0.50 mm for preparative chromatography) and visualized using a combination of UV, anisaldehyde, ceric ammonium molybdate, iodine, vanillin, and potassium permanganate staining techniques. Silicycle Siliaflash P60 (particle size 0.040–0.063 mm) was used for flash column chromatography. ^1H NMR spectra were recorded on Bruker spectrometers (at 500 MHz) and are reported relative to residual solvent signals. Data for ^1H NMR spectra are reported as follows: chemical shift (δ ppm), multiplicity, coupling constant (Hz), and integration. Data for ^{13}C NMR are reported in terms of chemical shift (at 125 MHz). IR spectra were recorded on a Perkin-Elmer 100 spectrometer and are reported in terms of frequency absorption (cm^{-1}). High-resolution mass spectra were obtained on Thermo Scientific™ Exactive Mass Spectrometer with DART ID-CUBE. DART-MS spectra were collected on a Thermo Exactive Plus MSD (Thermo Scientific) equipped with an ID-CUBE ion source and a Vapur Interface (IonSense Inc.). Both the source and MSD were controlled by Excalibur software v. 3.0. The analyte was spotted onto OpenSpot sampling cards (IonSense Inc.) using $CHCl_3$ as the solvent. Ionization was accomplished using UHP He (Airgas Inc.) plasma with no additional ionization agents. The mass calibration was carried out using Pierce LTQ Velos ESI (+) and (–) Ion calibration solutions (Thermo Fisher Scientific). Optical rotations were measured with a Rudolf Autopol III Automatic Polarimeter. Determination of enantiopurity was carried out on an Agilent 1260 HPLC (high performance liquid chromatography) and a Mettler Toledo SFC (supercritical fluid chromatography) using a Daicel ChiralPak OJ-H columns and Daicel ChiralPak IC-H.

Experimental Procedure

A. Syntheses of Starting Materials

The following amides were synthesized following known protocols: cyclohexylamide **7**,[1] cyclopentylamide **SI-1**,[1] *tert*-butylamide **SI-2**,[1] and **40**.[1]

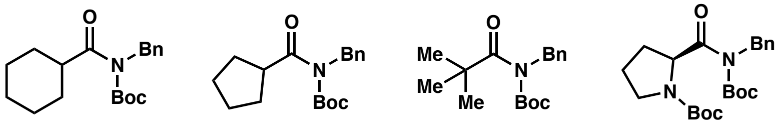

7 *SI-1* *SI-2* *40*

Syntheses for the remaining substrates shown in Figures 2, 3, 5, and 6 are as follows:

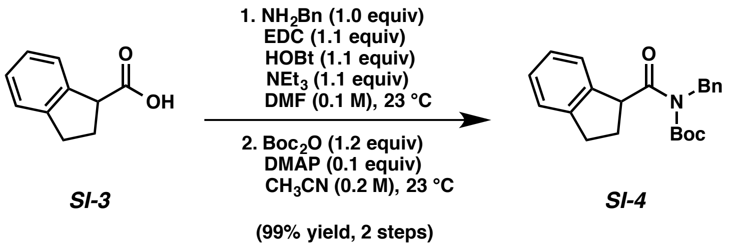

Amide SI-4: To a mixture of carboxylic acid **SI-3** (1.0 g, 6.17 mmol, 1.0 equiv), EDC (1.30 g, 6.79 mmol, 1.1 equiv), HOBt (917 mg, 6.79 mmol, 1.1 equiv), triethylamine (0.94 mL, 6.79 mmol, 1.1 equiv), and DMF (61.7 mL, 0.1 M) was added benzylamine (0.74 mL, 6.79 mmol, 1.1 equiv). The resulting mixture was stirred at 23 °C for 12 h, and then diluted with deionized water (50 mL) and EtOAc (50 mL). The layers were separated and the organic layer was washed successively with 1.0 M HCl (50 mL), saturated aqueous $NaHCO_3$ (50 mL), and brine (50 mL). The organic layer was dried over Na_2SO_4 and concentrated under reduced pressure. The resulting crude solid material was used in the subsequent step without further purification.

To a flask containing the crude material from the previous step was added DMAP (75 mg, 0.62 mmol, 0.1 equiv) followed by acetonitrile (31 mL, 0.2 M). Boc_2O (1.55 g, 7.40 mmol, 1.2 equiv) was added in one portion and the reaction vessel was flushed with N_2. The reaction mixture was allowed to stir at 23 °C for 16 h. The reaction mixture was diluted with EtOAc (50 mL) and then washed with saturated aqueous $NaHCO_3$ (2 x 50 mL) and brine (50 mL). The organic layer was dried over Na_2SO_4 and concentrated under reduced pressure. The resulting crude residue was purified by flash chromatography (29:1 Hexanes:EtOAc) to yield amide **SI-4** (3.0 g, 99% yield, over two steps) as a white solid. Amide **SI-4**: mp: 52.8–54.5 °C; R_f 0.56 (5:1 Hexanes:EtOAc); ^{1}H NMR (500 MHz, $CDCl_3$): δ 7.33–7.27 (m, 2H), 7.26–7.21 (m, 3H), 7.21–

7.17 (m, 2H), 7.16–7.12 (m, 2H), 4.91 (s, 2H), 4.39 (quint, *J* = 8.3, 1H), 3.34–3.23 (m, 4H), 1.42 (s, 9H); ^{13}C NMR (125 MHz, $CDCl_3$): δ 178.3, 153.1, 142.0, 138.5, 128.5, 127.6, 127.3, 126.6, 124.4, 83.4, 48.0, 45.8, 37.2, 28.0; IR (film): 3022, 2979, 1732, 1693, 1368 cm^{-1}; HRMS-APCI (*m/z*) $[M + H]^+$ calcd for $C_{22}H_{26}NO_3$, 352.1913; found 352.1886.

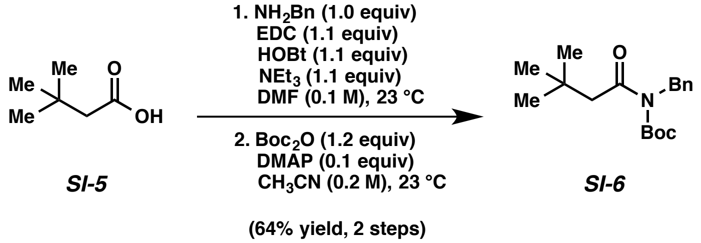

Amide SI-6: To a mixture of carboxylic acid **SI-5** (1.0 g, 8.61 mmol, 1.0 equiv), EDC (1.82 g, 9.47 mmol, 1.1 equiv), HOBt (1.28 g, 9.47 mmol, 1.1 equiv), triethylamine (1.31 mL, 9.47 mmol, 1.1 equiv), and DMF (86.1 mL, 0.1 M) was added benzylamine (1.03 mL, 9.47 mmol, 1.1 equiv). The resulting mixture was stirred at 23 °C for 12 h, and then diluted with deionized water (50 mL) and EtOAc (50 mL). The layers were separated and the organic layer was washed successively with 1.0 M HCl (50 mL), saturated aqueous $NaHCO_3$ (50 mL), and brine (50 mL). The organic layer was dried over Na_2SO_4 and concentrated under reduced pressure. The resulting crude solid material was used in the subsequent step without further purification.

To a flask containing the crude material from the previous step was added DMAP (105 mg, 0.86 mmol, 0.1 equiv) followed by acetonitrile (43 mL, 0.2 M). Boc_2O (2.30 g, 10.3 mmol, 1.2 equiv) was added in one portion and the reaction vessel was flushed with N_2. The reaction mixture was allowed to stir at 23 °C for 24 h. The reaction mixture was diluted with EtOAc (50 mL) and then washed with saturated aqueous $NaHCO_3$ (2 x 50 mL) and brine (50 mL). The combined organic layers were dried over Na_2SO_4 and concentrated under reduced pressure. The resulting crude residue was purified by flash chromatography (49:1 Hexanes:EtOAc) to yield amide **SI-6** (675 mg, 64% yield, over two steps) as a clear oil. Amide **SI-6**: R_f 0.70 (5:1 Hexanes:EtOAc); ^{1}H NMR (500 MHz, $CDCl_3$): δ 7.31–7.26 (m, 2H), 7.26–7.19 (m, 3H), 4.87 (s, 2H), 2.91 (s, 2H), 1.39 (s, 9H), 1.03 (s, 9H); ^{13}C NMR (125 MHz, $CDCl_3$): δ 175.0, 153.6, 138.6, 128.4, 127.7, 127.1, 83.1, 49.3, 47.5, 31.7, 29.9, 28.0; IR (film): 2955, 2869, 1731, 1689, 1355 cm^{-1}; HRMS-APCI (*m/z*) $[M + H]^+$ calcd for $C_{18}H_{28}NO_3$, 306.2069; found 306.2047.

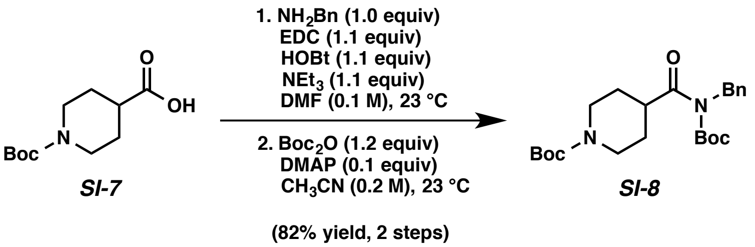

Amide SI-8: To a mixture of carboxylic acid **SI-7** (3.0 g, 13.1 mmol, 1.0 equiv), EDC (2.76 g, 14.4 mmol, 1.1 equiv), HOBt (1.94 g, 14.4 mmol, 1.1 equiv), triethylamine (1.99 mL, 14.4 mmol, 1.1 equiv), and DMF (131 mL, 0.1 M) was added benzylamine (1.57 mL, 14.4 mmol, 1.1 equiv). The resulting mixture was stirred at 23 °C for 17 h, and then diluted with deionized water (250 mL) and EtOAc (150 mL). The aqueous layer was extracted with EtOAc (3 x 150 mL). The organic layer was back-extracted with deionized water (3 x 60 mL). The organic layer was then dried over Na_2SO_4 and concentrated under reduced pressure. The resulting crude solid material was used in the subsequent step without further purification.

To a flask containing the crude material from the previous step was added DMAP (148 mg, 1.21 mmol, 0.1 equiv) followed by acetonitrile (60 mL, 0.2 M). Boc_2O (3.86 g, 15.7 mmol, 1.2 equiv) was added in one portion and the reaction vessel was flushed with N_2. The reaction mixture was allowed to stir at 23 °C for 43 h. The reaction mixture was diluted with saturated aqueous $NaHCO_3$ (200 mL), EtOAc (200 mL), and deionized water (200 mL). The aqueous layer was extracted with EtOAc (3 x 150 mL). The combined organic layers were then dried over Na_2SO_4 and concentrated under reduced pressure. The resulting crude residue was purified by flash chromatography (9:1 Hexanes:EtOAc) and then recrystallized from heptanes to yield amide **SI-8** (3.6 g, 82% yield, over two steps) as a white solid. Amide **SI-8**: mp: 84.2–85.3 °C; R_f 0.32 (5:1 Hexanes:EtOAc); ^{1}H NMR (500 MHz, $CDCl_3$, 14 of 15 carbons observed): δ 7.31–7.26 (m, 2H), 7.25–7.18 (m, 3H), 4.87 (s, 2H), 4.13 (br s, 2H), 3.60 (tt, J = 11.3, 3.3, 1H), 2.80 (br s, 2H), 1.93–1.77 (m, 2H), 1.72–1.60 (m, 2H), 1.46 (s, 9H), 1.40 (s, 9H); ^{13}C NMR (125 MHz, $CDCl_3$): δ 178.3, 154.9, 153.1, 138.4, 128.5, 127.5, 127.3, 83.5, 79.6, 47.8, 43.1, 29.0, 28.6, 28.0; IR (film): 2977, 2927, 2864, 1732, 1693 cm^{-1}; HRMS-APCI (*m/z*) [M + H]$^+$ calcd for $C_{23}H_{35}N_2O_5$, 419.2546; found 419.2546.

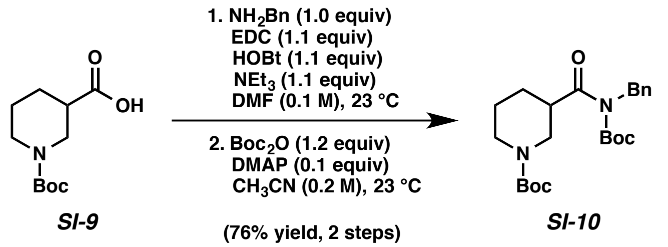

Amide SI-10: To a mixture of carboxylic acid **SI-9** (3.0 g, 13.1 mmol, 1.0 equiv), EDC (2.76 g, 14.4 mmol, 1.1 equiv), HOBt (1.94 g, 14.4 mmol, 1.1 equiv), triethylamine (1.99 mL, 14.4 mmol, 1.1 equiv), and DMF (131 mL, 0.1 M) was added benzylamine (1.57 mL, 14.4 mmol, 1.1 equiv). The resulting mixture was stirred at 23 °C for 17 h, and then diluted with deionized water (250 mL) and EtOAc (150 mL). The aqueous layer was extracted with EtOAc (3 x 150 mL). The organic layer was back-extracted with deionized water (3 x 30 mL). The combined organic layers were dried over Na_2SO_4 and concentrated under reduced pressure. The resulting crude solid material was used in the subsequent step without further purification.

To a flask containing the crude material from the previous step was added DMAP (160 mg, 1.31 mmol, 0.1 equiv) followed by acetonitrile (60 mL, 0.2 M). Boc_2O (3.72 g, 17.0 mmol, 1.2 equiv) was added in one portion and the reaction vessel was flushed with N_2. The reaction mixture was allowed to stir at 23 °C for 43 h. The reaction mixture was diluted with saturated aqueous $NaHCO_3$ (200 mL), EtOAc (200 mL) and deionized water (200 mL). The aqueous layer was extracted with EtOAc (3 x 150 mL). The combined organic layers were dried over Na_2SO_4 and concentrated under reduced pressure. The resulting crude residue was purified by flash chromatography (9:1 Hexanes:EtOAc) to yield amide **SI-10** (2.8 g, 76% yield, over two steps) as a white solid. Amide **SI-10**: mp: 75.1–79.1 °C; R_f 0.62 (5:1 Hexanes:EtOAc); ^{1}H NMR (500 MHz, $CDCl_3$): δ 7.32–7.26 (m, 2H), 7.26–7.17 (m, 3H), 4.94–4.77 (m, 2H), 4.33–3.87 (m, 2H), 3.56–3.46 (m, 1H), 2.99 (app t, *J* = 12.2, 1H), 2.75 (br s, 1H), 2.11 (br s, 1H), 1.76–1.67 (m, 1H), 1.63–1.48 (m, 2H), 1.45 (s, 9H), 1.41 (s, 9H); ^{13}C NMR (125 MHz, $CDCl_3$, 15 of 17 carbons observed): δ 177.2, 154.9, 152.9, 138.3, 128.5, 127.5, 127.3, 83.6, 79.7, 47.7, 43.5, 28.7, 28.6, 28.0, 24.8; IR (film): 2977, 2935, 1732, 1689, 1367 cm^{-1}; HRMS-APCI (*m/z*) $[M + H]^+$ calcd for $C_{23}H_{35}N_2O_5$, 419.2546; found 419.2516.

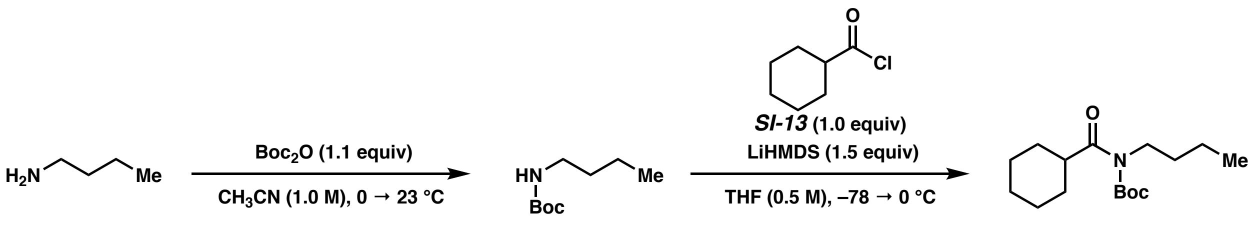

SI-11 *SI-12* *22*

Amide 22: To a solution of *n*-BuNH_2 (**SI-11**, 1.0 g, 13.67 mmol, 1.0 equiv) in CH_3CN (8.7 mL) maintained at 0 °C was added Boc_2O (3.28 g, 15.05 mmol, 1.1 equiv) dropwise (1 drop/sec) as a solution in CH_3CN (5 mL). The ice bath was removed and the resulting mixture was warmed to 23 °C and stirred an additional 0.5 h. The reaction was concentrated under reduced pressure, then subsequently re-suspended in CH_2Cl_2 (50 mL), and transferred to a separatory funnel with deionized water (30 mL). The layers were separated and the organic layer was washed with deionized water (3 x 30 mL). The organic layer was dried over Na_2SO_4 and concentrated under reduced pressure to yield **SI-12** (1.45 g, 59% yield) as a white solid. The resulting crude solid material was used in the subsequent step without further purification.

To a flask containing crude **SI-12** (1.4 g, 8.08 mmol, 1.0 equiv) under N_2 from the previous step, THF (6 mL) was added. The resultant solution was cooled to –78 °C. A solution of LiHMDS (2.03 g, 12.12 mmol, 1.5 equiv) in THF (6 mL) was then added dropwise (1 drop/sec) at –78 °C with vigorous stirring. The resulting mixture was stirred at –78 °C for 1 h. Acid chloride **SI-13** (1.08 mL, 8.08 mmol, 1.0 equiv) was then added dropwise (1 drop/sec) at –78 °C and stirred for an additional 1 h. This mixture was then warmed to 0 °C and stirred for an additional 1 h. The reaction was warmed to 23 °C before quenching with saturated aqueous $NaHCO_3$ (30 mL). This biphasic solution was transferred to a separatory funnel with brine (10 mL) and EtOAc (30 mL). The layers were separated and the aqueous layer was extracted with EtOAc (3 x 30 mL). The combined organic layers were dried over Na_2SO_4 and concentrated under reduced pressure. The resulting crude residue was purified by flash chromatography (29:1 Hexanes:Et_2O) to yield amide **22** (1.99 g, 86% yield, over two steps) as a colorless oil. Amide **22**: R_f 0.37 (19:1 Hexanes: Et_2O); ^{1}H NMR (500 MHz, $CDCl_3$): δ 3.65–3.60 (m, 2H), 3.29 (tt, *J* = 11.3, 3.2, 1H), 1.88–1.80 (m, 2H), 1.80–1.73 (m, 2H), 1.70–1.73 (m, 1H), 1.53 (s, 9H), 1.50–1.38 (m, 4H), 1.35–1.20 (m, 5H), 0.91 (t, *J* = 7.3, 3H); ^{13}C NMR (125 MHz, $CDCl_3$): δ 179.7, 153.5, 82.6, 45.0, 44.8, 31.1, 30.0, 28.2, 26.1, 26.0, 20.2, 14.0; IR (film): 2931, 1855, 1729,

1693, 1369, 1147 cm^{-1}; HRMS-APCI (*m/z*) [M + H]$^+$ calcd for $C_{23}H_{35}N_2O_5$, 284.2226; found 284.2208.

*Note: Amide **22** can be prepared from the corresponding N-Bu,H secondary amide using the procedure outlined below. The procedure described above was used for preparative scale synthesis of **22** as it was higher yielding and operationally simpler.*

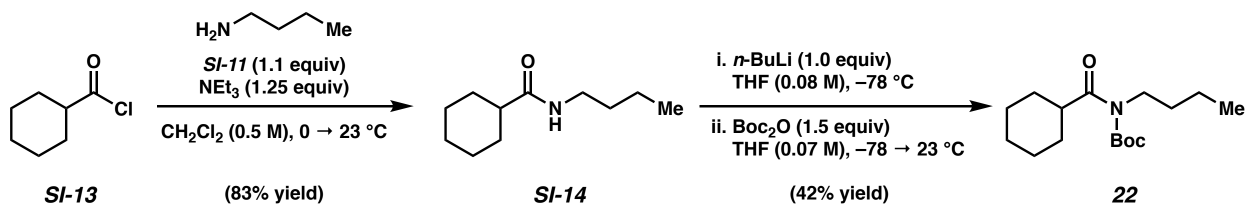

Amide 22: To a solution of **SI-13** (1.0 g, 6.80 mmol, 1.0 equiv) and NEt_3 (1.20 mL, 8.50 mmol, 1.25 equiv) in CH_2Cl_2 (13.6 mL) maintained at 0 °C was added **SI-11** (738 μL, 7.48 mmol, 1.1 equiv) dropwise (1 drop/sec). The ice bath was removed and the resulting mixture was warmed to 23 °C and stirred an additional 2.5 h. The reaction was quenched with deionized water (20 mL) and 1 N HCl (4 mL), and then transferred to a separatory funnel with EtOAc (20 mL). The layers were separated and the aqueous layer was extracted with EtOAc (3 x 20 mL). The combined organic layers were dried over Na_2SO_4 and concentrated under reduced pressure to yield **SI-14** (1.04 g, 83% yield) as a white solid. The resulting crude solid material was used in the subsequent step without further purification.

To a flask containing crude **SI-14** (250 mg, 1.36 mmol, 1.0 equiv) under N_2 from the previous step, THF (17 mL, 0.08 M) was added. The resultant solution was cooled to –78 °C. A solution of *n*-BuLi (2.20 M in hexanes, 618 μL, 1.36 mmol, 1.0 equiv) was then added dropwise (1 drop/sec) at –78 °C with vigorous stirring. The resulting mixture was stirred at –78 °C for 15 min. A solution of Boc_2O (445 mg, 2.04 mmol, 1.50 equiv) in THF (4.1 mL, 0.5 M) was then added dropwise (1 drop/sec) at –78 °C and stirred for an additional 1 h. This mixture was then warmed to 0 °C and stirred for an additional 1 h. The reaction was warmed to 23 °C before quenching with saturated aqueous NH_4Cl (20 mL). This biphasic solution was transferred to a separatory funnel with deionized water (10 mL) and EtOAc (20 mL). The layers were separated and the aqueous layer was extracted with EtOAc (3 x 20 mL). The combined organic layers were

dried over Na_2SO_4 and concentrated under reduced pressure. The resulting crude residue was purified by flash chromatography (49:1 Hexanes:Et_2O) and the remaining Boc_2O was removed by evaporation at 70 °C under high vacuum to yield amide **22** (161 mg, 42% yield) as a colorless oil. Amide **22**: R_f 0.37 (19:1 Hexanes: Et_2O); Spectral data match those previously reported for amide **22** (see page S7).

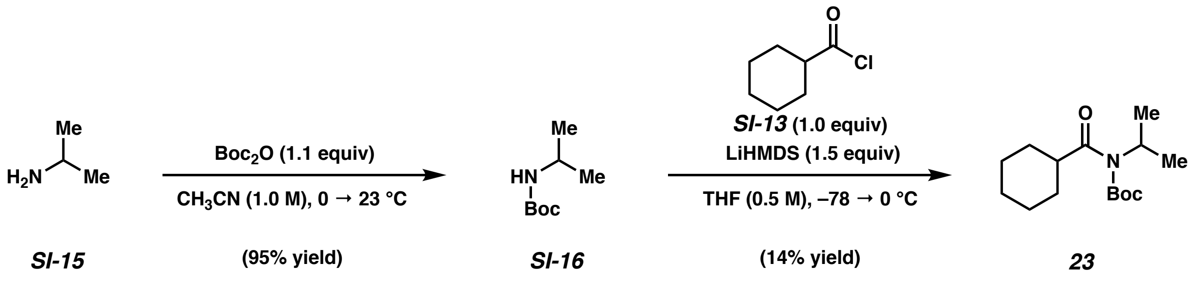

Amide 23: To a solution of *i*-$PrNH_2$ (**SI-15**, 1.0 g, 16.95 mmol, 1.0 equiv) in CH_3CN (10 mL) maintained at 0 °C was added Boc_2O (4.07 g, 18.65 mmol, 1.1 equiv) dropwise (1 drop/sec) as a solution in CH_3CN (7 mL). The ice bath was removed and the resulting mixture was warmed to 23 °C and stirred an additional 0.5 h. The reaction was concentrated under reduced pressure, then subsequently re-suspended in CH_2Cl_2 (50 mL), and transferred to a separatory funnel with deionized water (30 mL). The layers were separated and the organic layer was washed with deionized water (3 x 30 mL). The organic layer was dried over Na_2SO_4 and concentrated under reduced pressure to yield **SI-16** (2.56 g, 95% yield) as a white solid. The resulting crude solid material was used in the subsequent step without further purification.

To a flask containing crude **SI-16** (2.25 g, 14.2 mmol, 1.0 equiv) under N_2 from the previous step, THF (14.2 mL) was added. The resultant solution was cooled to –78 °C. A solution of LiHMDS (3.36 g, 21.2 mmol, 1.5 equiv) in THF (14.2 mL) was then added dropwise (1 drop/sec) at –78 °C with vigorous stirring. The resulting mixture was stirred at –78 °C for 1 h. Acid chloride **SI-13** (1.89 mL, 14.2 mmol, 1.0 equiv) was then added dropwise (1 drop/sec) at –78 °C and stirred for an additional 1 h. This mixture was then warmed to 0 °C and stirred for an additional 1 h. The reaction was warmed to 23 °C before quenching with saturated aqueous $NaHCO_3$ (30 mL). This biphasic solution was transferred to a separatory funnel with brine (10 mL) and EtOAc (30 mL). The layers were separated and the aqueous layer was extracted with EtOAc (3 x 30 mL). The combined organic layers were dried over Na_2SO_4 and concentrated under reduced pressure. The resulting crude residue was purified by flash chromatography (49:1

Hexanes:Et_2O) to yield amide **23** (537 mg, 14% yield) as a colorless oil. Amide **23**: R_f 0.35 (19:1 Hexanes: Et_2O); ^1H NMR (500 MHz, $CDCl_3$): δ 4.68 (septet, *J* = 6.8, 1H), 2.95 (tt, *J* = 11.4, 3.2, 1H), 1.87–1.81 (m, 2H), 1.80–1.73 (m, 2H), 1.69–1.63 (m, 1H), 1.54 (s, 9H), 1.48–1.37 (m, 3H), 1.27–1.22 (m, 8H); ^{13}C NMR (125 MHz, $CDCl_3$): δ 179.7, 154.0, 82.9, 47.1, 45.8, 30.0, 28.2, 26.0, 25.95, 20.6; IR (film): 2974, 2932, 2855, 1727, 1687, 1368 cm^{-1}; HRMS-APCI (*m/z*) [M + H]$^+$ calcd for $C_{23}H_{35}N_2O_5$, 270.2069; found 270.2051.

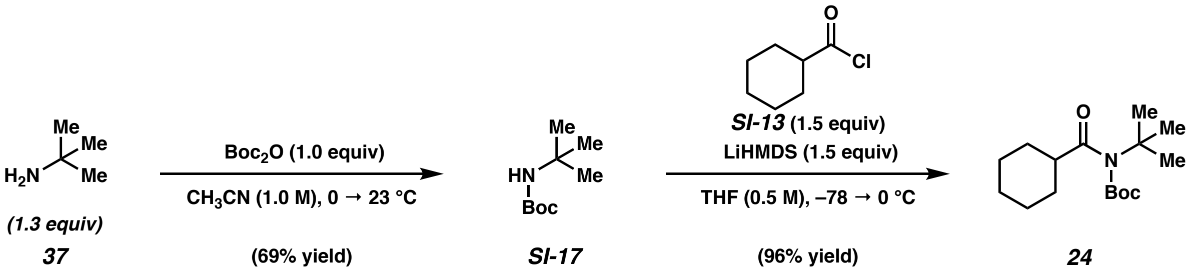

Amide 24: To a solution of *t*-BuNH_2 (**37**, 1.42 g, 19.5 mmol, 1.3 equiv) in CH_3CN (10 mL) maintained at 0 °C was added Boc_2O (3.27 g, 15.0 mmol, 1.0 equiv) dropwise (1 drop/sec) as a solution in CH_3CN (5 mL). The ice bath was removed and the resulting mixture was warmed to 23 °C and stirred an additional 0.5 h. The reaction was concentrated under reduced pressure, subsequently resuspended in CH_2Cl_2 (50 mL), and transferred to a separatory funnel with deionized water (30 mL). The layers were separated and the organic layer was washed with deionized water (3 x 30 mL). The organic layer was dried over Na_2SO_4 and concentrated under reduced pressure to yield **SI-17** (1.80 g, 69% yield) as a white solid. The resulting crude solid material was used in the subsequent step without further purification.

To a flask containing crude **SI-17** (1.0g, 5.78 mmol, 1.0 equiv) under N_2 from the previous step, THF (6 mL) was added. The resultant solution was cooled to –78 °C. A solution of LiHMDS (1.45 g, 8.67 mmol, 1.5 equiv) in THF (6 mL) was then added dropwise (1 drop/sec) at –78 °C with vigorous stirring. The resulting mixture was stirred at –78 °C for 1 h. Acid chloride **SI-13** (1.16 mL, 8.67 mmol, 1.5 equiv) was then added dropwise (1 drop/sec) at –78 °C and stirred for an additional 1 h. This mixture was then warmed to 0 °C and stirred for an additional 1 h. The reaction was warmed to 23 °C before quenching with saturated aqueous $NaHCO_3$ (30 mL). This biphasic solution was transferred to a separatory funnel with brine (10 mL) and EtOAc (30 mL). The layers were separated and the aqueous layer was extracted with EtOAc (3 x 30 mL). The combined organic layers were dried over Na_2SO_4 and concentrated

under reduced pressure. The resulting crude residue was purified by flash chromatography (49:1 Hexanes:EtOAc) to yield amide **24** (1.57 g, 96% yield) as a white solid. Amide **24**: R_f 0.19 (19:1 Hexanes: Et_2O); ^{1}H NMR (500 MHz, $CDCl_3$): δ 2.26 (tt, *J* = 11.3, 3.1, 1H), 1.87–1.74 (m, 4H), 1.69–1.63 (m, 1H), 1.53 (s, 9H), 1.50–1.40 (m, 11H), 1.27–1.12 (m, 3H); ^{13}C NMR (125 MHz, $CDCl_3$): δ 176.2, 155.0, 83.6, 57.6, 46.4, 29.7, 28.5, 27.7, 25.9, 25.87; IR (film): 2977, 2932, 2855, 1739, 1703, 1677 cm^{-1}; HRMS-APCI (*m/z*) [M + H]$^+$ calcd for $C_{16}H_{31}NO_3$, 284.2226; found 284.2210.

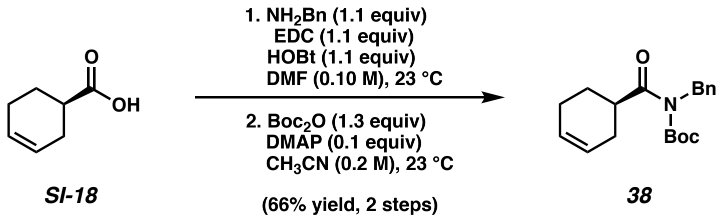

SI-18 *38*

Amide 38. To a mixture of carboxylic acid **SI-18** (1.0 g, 7.9 mmol, 1.0 equiv), EDC (1.67 g, 8.7 mmol, 1.1 equiv), HOBt (1.2 g, 8.7 mmol, 1.1 equiv), and DMF (79 mL, 0.1 M) was added benzylamine (0.95 mL, 8.7 mmol, 1.1 equiv). The resulting mixture was stirred at 23 °C for 22 h, and then diluted with deionized water (50 mL) and EtOAc (50 mL). The aqueous layer was extracted with EtOAc (3 x 30 mL). The organic layer was back-extracted with deionized water (3 x 30 mL). The organic layer was then dried over Na_2SO_4 and concentrated under reduced pressure. The resulting crude solid material was used in the subsequent step without further purification.

To a flask containing the crude material from the previous step was added DMAP (97 mg, 0.79 mmol, 0.1 equiv) followed by acetonitrile (40 mL, 0.2 M). Boc_2O (2.25 g, 10.3 mmol, 1.3 equiv) was added in one portion and the reaction vessel was flushed with N_2. The reaction mixture was allowed to stir at 23 °C for 20 h. The reaction mixture was diluted with saturated aqueous $NaHCO_3$ (40 mL), EtOAc (50 mL) and deionized water (40 mL). The aqueous layer was extracted with EtOAc (3 x 50 mL). The combined organic layers were then dried over Na_2SO_4 and concentrated under reduced pressure. The resulting crude residue was purified by flash chromatography (19:1 Hexanes:EtOAc) to yield amide **38** (5.3 g, 66% yield, over two steps) as a clear oil. Amide **38**: R_f 0.63 (5:1 Hexanes:EtOAc); ^{1}H NMR (500 MHz, $CDCl_3$): δ 7.32–7.26 (m, 2H), 7.25–7.20 (m, 3H), 5.74–5.64 (m, 2H), 4.88 (dd, *J* = 18.1, 14.9, 2H), 3.64 (m, 1H), 2.33–2.21 (m, 2H), 2.19–2.06 (m, 2H), 2.00–1.93 (m, 1H), 1.78–1.65 (m, 1H), 1.46–1.36 (s, 9H);

^{13}C NMR (125 MHz, $CDCl_3$): δ 179.6, 153.2, 138.6, 128.4, 127.6, 127.2, 126.7, 125.8, 83.3, 47.8, 41.1, 28.6, 28.0, 26.3, 25.1; IR (film): 3028, 2978, 1732, 1693, 1369 cm^{-1}; HRMS-APCI (*m/z*) $[M + H]^+$ calcd for $C_{19}H_{26}NO_3$, 316.1913; found 316.1888; $[\alpha]^{23.4}_D$ –20.40 ° (c = 1.00, $CHCl_3$).

B. Reaction Discovery, Optimization, and Relevant Control Experiments

Representative procedure for ligand screening (coupling of amide 7 and cyclohexylamine (8) is used as an example). Amide 9 (Table S1, entry 3): A 1-dram vial containing a magnetic stir bar was flame-dried under reduced pressure, and then allowed to cool under N_2. The vial was charged with amide substrate **7** (63.4 mg, 0.2 mmol, 1.0 equiv), 1,3,5-trimethoxybenzene (TMB) (10.1 mg, 0.06 mmol, 0.3 equiv), and the vial was flushed with N_2. The vial was then taken into a glove box and charged with 1,3-dicyclohexylbenzimidazolium chloride (**12**, 0.04 mmol, 20 mol%), $Ni(cod)_2$ (5.5 mg, 0.02 mmol, 10 mol%), and NaO*t*Bu (4.2 mg, 0.044 mmol, 22 mol%). Subsequently, cyclohexylamine (**8**) (46 mL, 0.4 mmol, 2.0 equiv) and then toluene (0.2 mL, 1.0 M) were added. The vial was sealed with a Teflon-lined screw cap, removed from the glove box, and stirred at 60 °C for 24 h. After cooling to 23 °C, the mixture was diluted with hexanes (0.5 mL) and filtered over a plug of silica gel (10 mL of EtOAc eluent). The volatiles were evaporated under reduced pressure, and the yield was determined by ^1H NMR analysis with 1,3,5-trimethoxybenzene as an internal standard.

Optimization efforts that deviate from the above conditions are indicated below.

Table S1. Optimization of Reaction Conditions.[a]

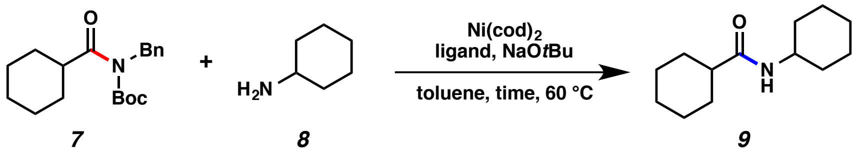

Entry	*mol% of Ni(cod)$_2$*	*Ligand (mol%)*	*mol% of NaOtBu*	*Equivs of 8*	*Conc.*	*Time*	*Yield of 9*
1	10	***10*** (20 mol%)	–	2.0	1.0	24 h	15%
2	10	***11*** (20 mol%)	–	2.0	1.0	24 h	0%
3	10	***12*** (20 mol%)	22	2.0	1.0	24 h	Quant. Yield
4	5	***12*** (10 mol%)	11	2.0	1.0	24 h	Quant. Yield
5	5	***12*** (10 mol%)	11	1.5	1.0	24 h	88%
6	5	***12*** (10 mol%)	11	1.5	1.5	18 h	90%
7	–	***12*** (10 mol%)	11	1.5	1.5	18 h	0%
8	–	–	–	1.5	1.5	18 h	0%

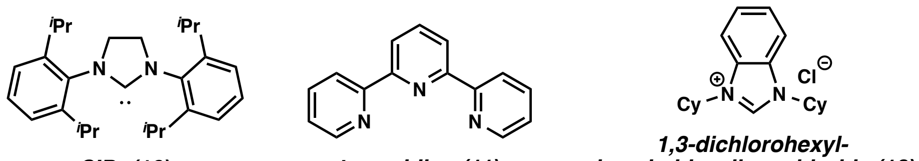

SIPr (10) ***terpyridine (11)*** ***1,3-dichlorohexyl-benzimidazolium chloride (12)***

[a] Yields were determined using ^{1}H NMR analysis with 1,3,5-trimethoxybenzene (TMB) as an internal standard.

C. Scope of Amide Substrates

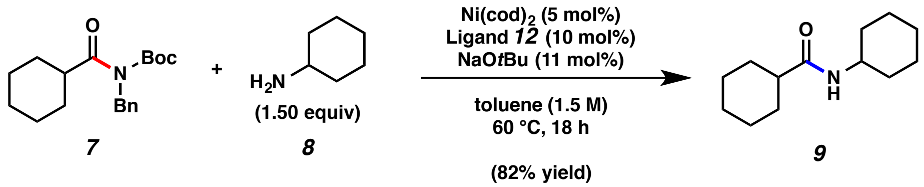

Representative Procedure (coupling of amide 7 and cyclohexylamine (8) is used as an example). Amide 9 (Figure 2): A 1-dram vial containing a magnetic stir bar was flame-dried under reduced pressure, and then allowed to cool under N_2. The vial was charged with amide substrate **7** (63.4 mg, 0.200 mmol, 1.0 equiv) and the vial was flushed with N_2. The vial was then taken into a glove box and charged with 1,3-dicyclohexylbenzimidazolium chloride (**12**, 6.4 mg, 0.020 mmol, 10 mol%), Ni(cod)$_2$ (2.8 mg, 0.010 mmol, 5 mol%), and NaO*t*Bu (2.1 mg, 0.022 mmol, 11 mol%). Subsequently, cyclohexylamine (**8**, 34 μL, 0.30 mmol, 1.5 equiv) and toluene (0.133 mL, 1.5 M) were added. The vial was sealed with a Teflon-lined screw cap, removed from the glove box, and stirred at 60 °C for 18 h. After cooling to 23 °C, the mixture was diluted with hexanes (0.5 mL) and filtered over a plug of silica gel (10 mL of EtOAc eluent). The volatiles were removed under reduced pressure and the crude residue was purified by flash chromatography (9:1 → 3:1 Hexanes:EtOAc) to generate amide **9** (82% yield, average of two experiments) as a white solid. Amide **9**: mp: 167.5–168.3 °C; R_f 0.42 (2:1 Hexanes:EtOAc); ^{1}H NMR (500 MHz, $CDCl_3$): δ 5.24 (br s, 1H), 3.81–3.69 (m, 1H), 2.00 (tt, J = 11.7, 3.4, 1H), 1.92–1.81 (m, 4H), 1.81–1.74 (m, 2H), 1.72–1.64 (m, 3H), 1.63–1.58 (m, 1H), 1.46–1.31 (m, 4H), 1.30–1.03 (m, 6H); ^{13}C NMR (125 MHz, $CDCl_3$): δ 175.2, 47.8, 45.9, 33.4, 29.9, 25.9, 25.7, 25.0 (8 of 9 carbons observed); IR (film): 3293, 2929, 2853, 1638, 1549 cm^{-1}; HRMS-APCI (*m/z*) [M + H]$^+$ calcd for $C_{13}H_{24}NO$, 210.1858; found 210.1842.

Note: It was found that the purity of both coupling partners had a significant effect on the yield of a given coupling. Additionally, maintaining a moderate stir rate (500–900 rpm) and ensuring all NaOtBu enters the reaction mixture are essential for the success of these couplings. Increased amounts of NaOtBu are not detrimental to the reaction and can help alleviate the latter of the aforementioned scenarios.

Any modifications of the conditions shown in the representative procedure above are specified in the following schemes, which depict all of the results shown in Figures 2 and 3.

*For each of the nickel-catalyzed reactions described herein, control experiments were performed concurrently where Ni(cod)$_2$ and both Ni(cod)$_2$ and ligand **12**/NaOtBu were omitted from the reactions. In all cases, these control experiments led to the recovery of the amide substrates with minimal (<5%) conversion to the corresponding amides.*

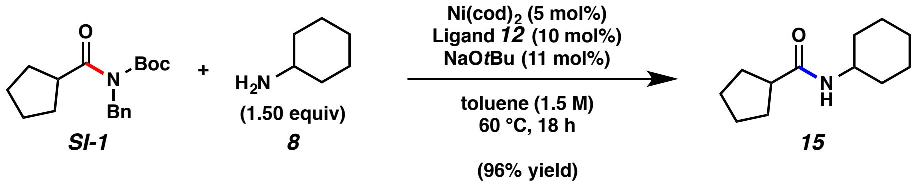

Amide 15 (Figure 2): Purification by flash chromatography (9:1 → 3:1 Hexanes:EtOAc) generated amide **15** (96% yield, average of two experiments) as a white solid. Amide **15**: mp: 157.1–159.3 °C; R_f 0.41 (2:1 Hexanes:EtOAc); ^{1}H NMR (500 MHz, $CDCl_3$): δ 5.25 (br s, 1H), 3.81–3.71 (m, 1H), 2.45 (app quintet, J = 7.8, 1H), 1.95–1.87 (m, 2H), 1.87–1.80 (m, 2H), 1.80–1.65 (m, 6H), 1.65–1.55 (m, 2H), 1.42–1.30 (m, 2H), 1.21–1.05 (m, 3H); ^{13}C NMR (125 MHz, $CDCl_3$): δ 175.3, 48.1, 46.2, 33.5, 30.6, 26.1, 25.7, 25.0; IR (film): 3298, 2934, 2854, 1640, 1545 cm^{-1}; HRMS-APCI (*m/z*) [M + H]$^+$ calcd for $C_{12}H_{22}NO$, 196.1701; found 196.1685.

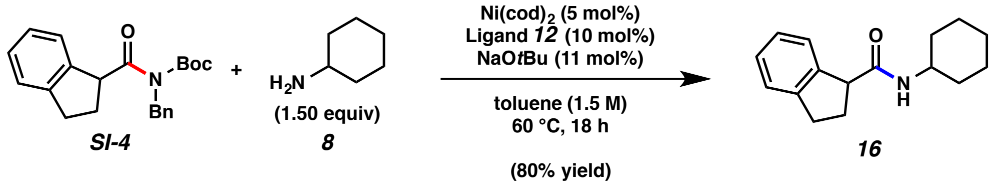

Amide 16 (Figure 2): Purification by flash chromatography (9:1 → 3:1 Hexanes:EtOAc) generated amide **16** (80% yield, average of two experiments) as a white solid. Amide **16**: mp: 175.2–178.5 °C; R_f 0.47 (2:1 Hexanes:EtOAc); ^{1}H NMR (500 MHz, $CDCl_3$): δ 7.23–7.18 (m, 2H), 7.17–7.12 (m, 2H), 5.36 (br s, 1H), 3.86–3.76 (m, 1H), 3.29–3.18 (m, 2H), 3.18–3.07 (m, 3H), 1.97–1.89 (m, 2H), 1.74–1.65 (m, 2H), 1.65–1.58 (m, 1H), 1.43–1.32 (m, 2H), 1.21–1.07 (m, 3H); ^{13}C NMR (125 MHz, $CDCl_3$): δ 173.8, 142.0, 126.7, 124.5, 48.3, 46.2, 37.1, 33.4, 25.7, 25.0; IR (film): 3296, 2933, 2854, 1633, 1541 cm^{-1}; HRMS-APCI (*m/z*) [M + H]$^+$ calcd for $C_{16}H_{22}NO$, 244.1701; found 244.1683.

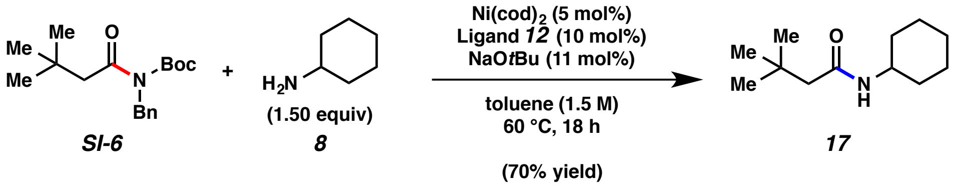

Amide 17 (Figure 2): Purification by flash chromatography (9:1 → 3:1 Hexanes:EtOAc) generated amide **17** (70% yield, average of two experiments) as a white solid. Amide **17**: mp: 146.5–148.8 °C; R_f 0.43 (2:1 Hexanes:EtOAc); ^{1}H NMR (500 MHz, $CDCl_3$): δ 5.18 (br s, 1H), 3.84–3.71 (m, 1H), 2.00 (s, 2H), 1.95–1.88 (m, 2H), 1.74–1.65 (m, 2H), 1.65–1.54 (m, 2H), 1.42–1.31 (m, 2H), 1.21–1.06 (m, 2H), 1.03 (s, 9H); ^{13}C NMR (125 MHz, $CDCl_3$): δ 170.8, 51.1, 48.1, 33.5, 31.0, 30.0, 25.7, 25.0; IR (film): 3284, 2931, 2853, 1634, 1550 cm^{-1}; HRMS-APCI (*m/z*) $[M + H]^+$ calcd for $C_{12}H_{24}NO$, 198.1858; found 198.1843.

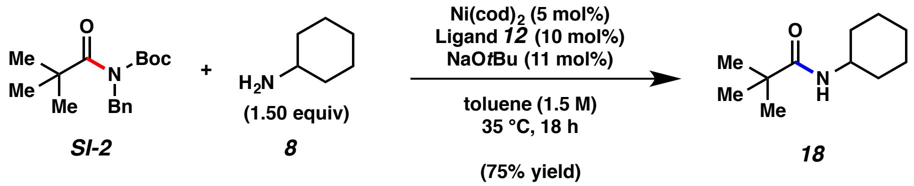

Amide 18 (Figure 2): Purification by flash chromatography (9:1 → 3:1 Hexanes:EtOAc) generated amide **18** (75% yield, average of two experiments) as a white solid. Amide **18**: mp: 121.8–123.1 °C; R_f 0.48 (2:1 Hexanes:EtOAc); ^{1}H NMR (500 MHz, $CDCl_3$): δ 5.43 (br s, 0.5H), 3.79–3.69 (m, 1H), 1.94–1.86 (m, 2H), 1.74–1.65 (m, 2H), 1.65–1.58 (m, 1H), 1.42–1.32 (m, 2H), 1.18 (s, 9H), 1.17–1.04 (m, 3H); ^{13}C NMR (125 MHz, $CDCl_3$): δ 177.6, 48.1, 38.7, 33.3, 27.8, 25.8, 25.0; IR (film): 3332, 2935, 2854, 1627, 1535 cm^{-1}; HRMS-APCI (*m/z*) $[M + H]^+$ calcd for $C_{11}H_{22}NO$, 184.1701; found 184.1687.

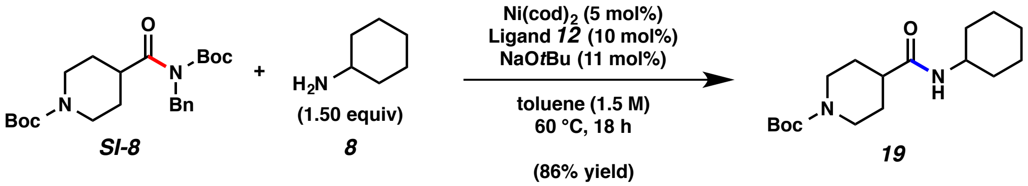

Amide 19 (Figure 2): Purification by flash chromatography (9:1 → 3:1 Hexanes:EtOAc) generated amide **19** (86% yield, average of two experiments) as a white solid. Amide **19**: mp: 148.9–150.2 °C; R_f 0.70 (100% EtOAc); ^{1}H NMR (500 MHz, $CDCl_3$): δ 5.30–5.23 (m, 1H), 4.14

(br s, 2H), 3.81–3.70 (m, 1H), 2.73 (m, 2H), 2.16 (tt, J = 11.6, 3.7, 1H), 1.94–1.86 (m, 2H), 1.82–1.75 (m, 2H), 1.73–1.66 (m, 2H), 1.66–1.58 (m, 3H), 1.45 (s, 9H), 1.42–1.31 (m, 2H), 1.21–1.03 (m, 3H); ^{13}C NMR (125 MHz, $CDCl_3$, (10 of 11 carbons observed): δ 173.5, 154.8, 79.7, 48.1, 43.7, 33.4, 28.9, 28.6, 25.7, 25.0; IR (film): 3300, 2931, 2855, 1697, 1642 cm^{-1}; HRMS-APCI (*m/z*) [M + H]$^+$ calcd for $C_{17}H_{31}N_2O_3$, 311.2335; found 311.2310.

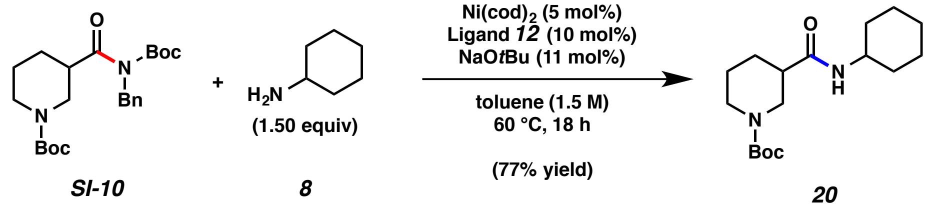

Amide 20 (Figure 2): Purification by flash chromatography (9:1 → 3:1 Hexanes:EtOAc) generated amide **20** (77% yield, average of two experiments) as a white solid. Amide **20**: mp: 136.2–138.9 °C; R$_f$ 0.48 (1:1 Hexanes:EtOAc); ^{1}H NMR (500 MHz, $CDCl_3$, 60 °C): δ 5.63 (br s, 1H), 3.91–3.66 (m, 3H), 3.33–3.21 (m, 1H), 3.15–3.02 (m, 1H), 2.28–2.20 (m, 1H), 1.96–1.79 (m, 4H), 1.75–1.67 (m, 2H), 1.67–1.59 (m, 2H), 1.48 (s, 9H), 1.44–1.33 (m, 3H), 1.25–1.09 (m, 3H); ^{13}C NMR (125 MHz, $CDCl_3$, 60 °C): δ 172.1, 155.2, 80.0, 48.4, 46.2, 44.7, 43.3, 33.4, 33.3, 28.7, 27.9, 25.8, 25.04, 25.02, 24.3; IR (film): 3303, 2855, 1696, 1642, 1544 cm^{-1}; HRMS-APCI (*m/z*) [M + H]$^+$ calcd for $C_{17}H_{31}N_2O_3$, 311.2335; found 311.2301.

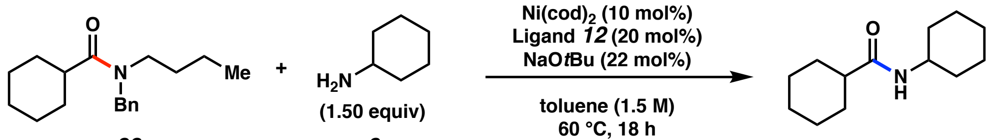

Amide 9 (Figure 3): Purification by flash chromatography (9:1 → 3:1 Hexanes:EtOAc) generated amide **9** (91% yield, average of two experiments) as a white solid. Amide **9**: R$_f$ 0.42 (2:1 Hexanes:EtOAc). Spectral data match those previously reported for amide **9** (see page S14).

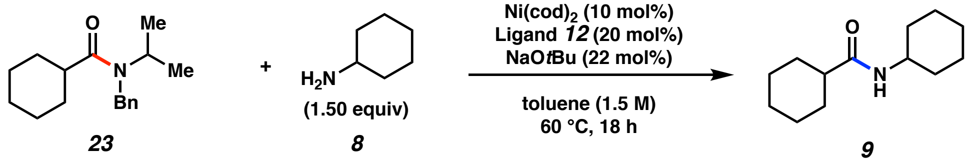

Amide 9 (Figure 3): Purification by flash chromatography (9:1 → 3:1 Hexanes:EtOAc) generated amide **9** (82% yield, average of two experiments) as a white solid. Amide **9**: R_f 0.42 (2:1 Hexanes:EtOAc). Spectral data match those previously reported for amide **9** (see page S14).

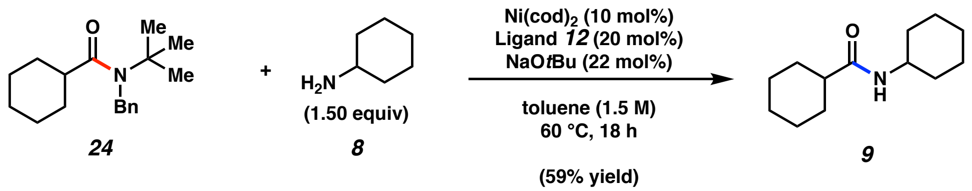

Amide 9 (Figure 3): Purification by flash chromatography (9:1 → 3:1 Hexanes:EtOAc) generated amide **9** (59% yield, average of two experiments) as a white solid. Amide **9**: R_f 0.42 (2:1 Hexanes:EtOAc). Spectral data match those previously reported for amide **9** (see page S14).

D. Scope of Amine Nucleophiles

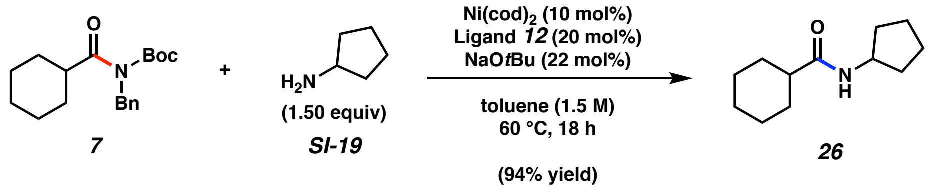

Representative Procedure (coupling of amide 7 and cyclopentylamine (SI-19) is used as an example). Amide 22 (Figure 4): A 1-dram vial containing a magnetic stir bar was flame-dried under reduced pressure, and then allowed to cool under N_2. The vial was charged with amide substrate **7** (63.4 mg, 0.200 mmol, 1.0 equiv) and the vial was flushed with N_2. The vial was then taken into a glove box and charged with 1,3-dicyclohexylbenzimidazolium chloride (**12**, 12.8 mg, 0.040 mmol, 20 mol%), $Ni(cod)_2$ (5.5 mg, 0.010 mmol, 10 mol%) and NaO*t*Bu (4.2 mg, 0.044 mmol, 22 mol%). Subsequently, cyclopentylamine (**SI-19**, 34 μL, 0.30 mmol, 1.5 equiv) and toluene (0.133 mL, 1.5 M) were added. The vial was sealed with a Teflon-lined screw cap, removed from the glove box, and stirred at 60 °C for 18 h. After cooling to 23 °C, the mixture was diluted with hexanes (0.5 mL) and filtered over a plug of silica gel (10 mL of EtOAc eluent). The volatiles were removed under reduced pressure and the crude residue was purified by flash chromatography (9:1 → 3:1 Hexanes:EtOAc) to generate amide **26** (94% yield, average of two experiments) as a white solid. Amide **26**: mp: 174.6–176.6 °C; R_f 0.39 (2:1 Hexanes:EtOAc); ^{1}H

NMR (500 MHz, $CDCl_3$): δ 5.33 (br s, 1H), 4.20 (sextet, J = 7.0, 1H), 2.04–1.95 (m, 3H), 1.85–1.76 (m, 4H), 1.67–1.57 (m, 5H), 1.45–1.37 (m, 2H), 1.36–1.29 (m, 2H), 1.27–1.22 (m, 3H); ^{13}C NMR (125 MHz, $CDCl_3$): δ 175.8, 51.0, 45.8, 33.4, 29.9, 25.92, 23.9; IR (film): 3265, 3074, 2929, 2852, 1704, 1635, 1552, 1453 cm^{-1}; HRMS-APCI (*m/z*) $[M + H]^+$ calcd for $C_{12}H_{22}NO_2$, 196.1701; found 196.1702.

Note: It was found that the purity of both coupling partners had a significant effect on the yield of a given coupling. Additionally, maintaining a moderate stir rate (500–900 rpm) and ensuring all NaOtBu enters the reaction mixture are essential for the success of these couplings. Increased amounts of NaOtBu are not detrimental to the reaction and can help alleviate the latter of the aforementioned scenarios.

Any modifications of the conditions shown in the representative procedure above are specified in the following schemes, which depict all of the results shown in Figure 4.

*For each of the nickel-catalyzed reactions described herein, control experiments were performed concurrently where Ni(cod)$_2$ and both Ni(cod)$_2$ and **12**/NaOtBu were omitted from the reactions. In all cases, these control experiments led to the recovery of the amide substrates with minimal (< 10%) detectable conversion to the corresponding amides.*

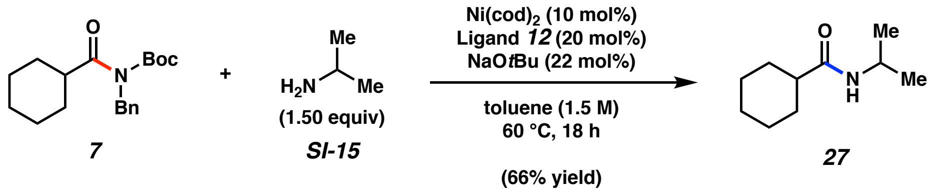

Amide 27 (Figure 4): Purification by flash chromatography (9:1 → 3:1 Hexanes:EtOAc) generated amide **27** (66% yield, average of two experiments) as a white solid. Amide **27**: R_f 0.29 (2:1 Hexanes:EtOAc). Spectral data match those previously reported.[2]

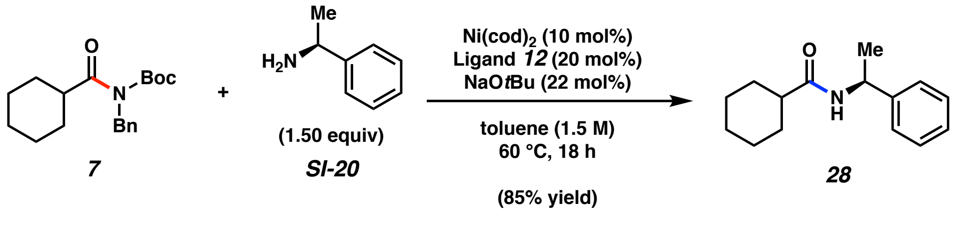

Amide 28 (Figure 4): Purification by flash chromatography (100% Hexanes → 9:1 Hexanes:EtOAc → 100% EtOAc) generated amide **28** (85% yield, average of two experiments) as a yellow solid Amide **28**: mp: 131.1–134.6 °C; R_f 0.46 (2:1 Hexanes:EtOAc); ^1H NMR (500

MHz, $CDCl_3$): δ 7.35–7.23 (m, 5H), 5.73–5.60 (m, 1H), 5.13 (quint, J = 7.2, 1H), 2.06 (tt, *J* = 11.7, 3.3, 1H), 1.88–1.77 (m, 4H), 1.67–1.64 (m, 1H), 1.48–1.38 (m, 5H), 1.31–1.15 (m, 3H); ^{13}C NMR (125 MHz, $CDCl_3$): δ 175.2, 143.6, 128.8, 127.4, 126.2, 48.4, 45.7, 29.83, 29.77, 25.9, 21.9; IR (film): 3346, 2926, 2855, 1643, 1520, 1450 cm^{-1}; HRMS-APCI (*m/z*) $[M + H]^+$ calcd for $C_{15}H_{22}NO$, 232.1701; found 232.1690; $[\alpha]^{20.1}_D$ –85.90° (c = 1.00, $CHCl_3$).

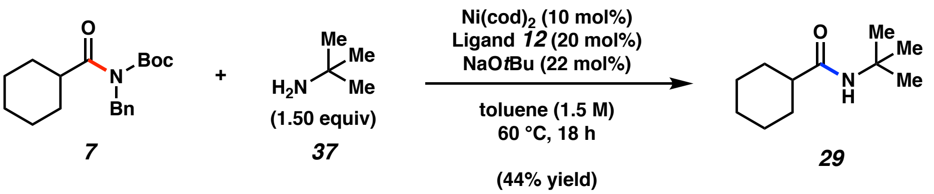

7 37 29

Amide 29 (Figure 4): Purification by preparative thin layer chromatography (5:1 Hexanes:EtOAc) generated amide **29** (44% yield, average of two experiments) as a white solid. Amide **29**: R_f 0.26 (5:1 Hexanes:EtOAc). Spectral data match those previously reported.[3]

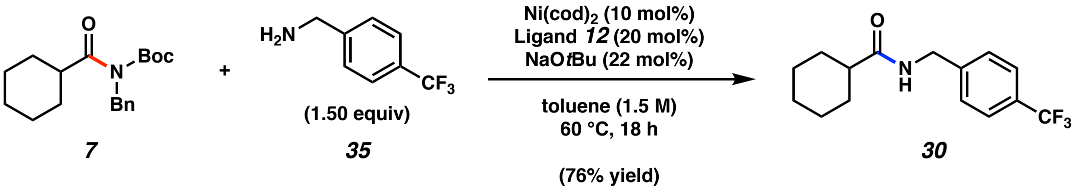

7 35 30

Amide 30 (Figure 4): Purification by flash chromatography (9:1 → 3:1 Hexanes:EtOAc) generated amide **30** (76% yield, average of two experiments) as a white solid. Amide **30**: mp: 135.9–137.4 °C; R_f 0.33 (2:1 Hexanes:EtOAc); ^1H NMR (500 MHz, $CDCl_3$): δ 7.58 (d, *J* = 8.1, 2H), 7.38 (d, *J* = 8.0, 2H), 5.78 (br s, 1H), 4.50 (d, *J* = 6.1, 2H), 2.13 (tt, *J* = 11.7, 3.5, 1H), 1.91–1.88 (m, 2H), 1.83–1.79 (m, 2H), 1.70–1.67 (m, 1H), 1.52–1.43 (m, 2H), 1.33–1.17 (m, 3H); ^{13}C NMR (125 MHz, $CDCl_3$, 10 of 11 carbons observed): δ 176.2, 142.9, 130.0, 129.7, 128.0, 125.8 (quartet, $J_{C–F}$ = 3.8), 45.7, 43.0, 29.9, 25.8; ^{19}F NMR (282 MHz, $CDCl_3$): δ –62.5; IR (film): 3291, 2931, 2856, 1643, 1549, 1328 cm^{-1}; HRMS-APCI (*m/z*) $[M + H]^+$ calcd for $C_{15}H_{19}F_3NO$, 286.1419; found 286.1405.

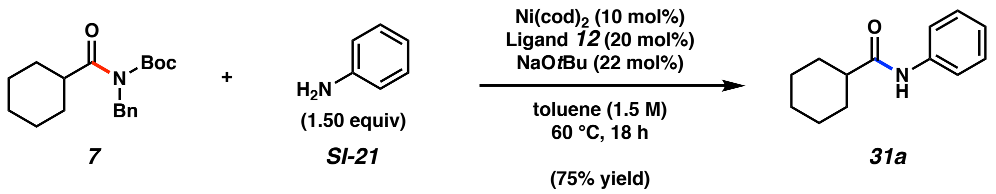

Amide 31a (Figure 4): Purification by flash chromatography (9:1 → 3:1 Hexanes:EtOAc) generated amide **31a** (75% yield, average of two experiments) as an off-white solid. Amide **31a**: R_f 0.25 (5:1 Hexanes:EtOAc). Spectral data match those previously reported.[4]

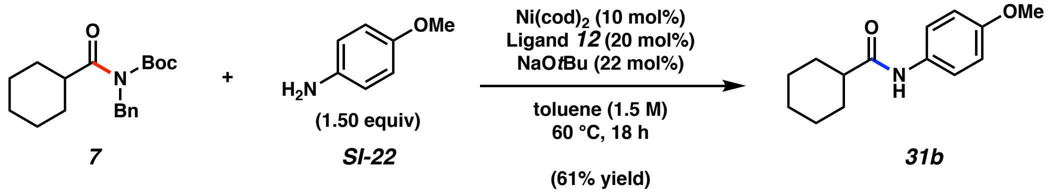

Amide 31b (Figure 4): Purification by flash chromatography (9:1 → 3:1 Hexanes:EtOAc) generated amide **31b** (61% yield, average of two experiments) as an off-white solid. Amide **31b**: R_f 0.23 (5:1 Hexanes:EtOAc). Spectral data match those previously reported.[5]

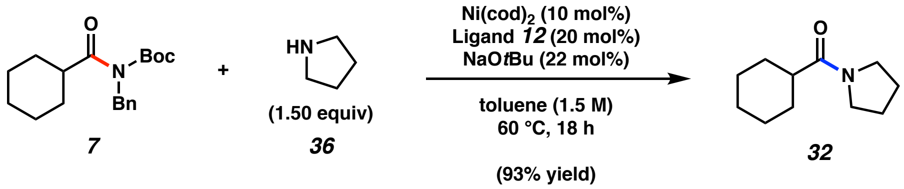

Amide 32 (Figure 4): Purification by preparative thin layer chromatography (2:1 Hexanes:EtOAc) generated amide **32** (93% yield, average of two experiments) as a white solid. Amide **32**: R_f 0.26 (5:1 Hexanes:EtOAc). Spectral data match those previously reported.[6]

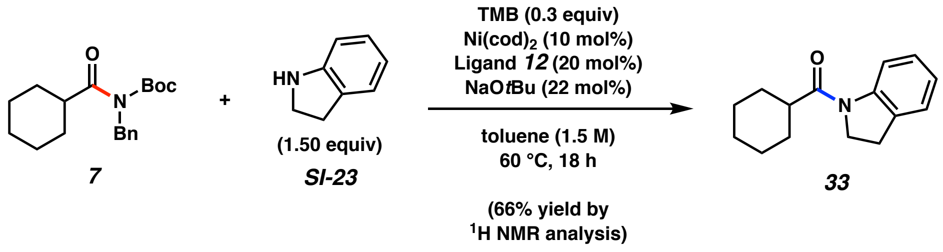

Amide 33 (Figure 4): Purification by preparative thin layer chromatography (2:1 Hexanes:EtOAc) generated amide **33** as an inseparable mixture with the HNBnBoc byproduct. To obtain an authentic sample of the product, to a flame dried scintillation vial containing the

mixture was added a stir bar and 0.100 mL TFA. The vial was sealed with a Teflon-lined screw cap and stirred at 23 °C for 3 h. The reaction was quenched by the slow addition of solid $NaHCO_3$ until bubbling ceased. The reaction mixture was diluted with EtOAc (25 mL) and deionized water (20 mL). The aqueous layer was extracted with EtOAc (3 x 25 mL). The combined organic layers were then dried over Na_2SO_4, filtered, and then concentrated under reduced pressure to yield amide **33** (66% crude ^{1}H NMR yield using TMB as an internal standard, average of two experiments) as a white solid. Amide **33**: mp: 133.6–135.2 °C; R_f 0.68 (2:1 Hexanes:EtOAc); ^{1}H NMR (500 MHz, $CDCl_3$): δ 8.26 (d, *J* = 8.0, 1H), 7.20–7.17 (m, 2H), 7.00 (dt, *J* = 7.3, 0.8 1H), 4.13 (t, *J* = 8.6, 2H), 3.19 (app t, *J* = 8.2, 2H), 2.46 (app t, *J* = 11.5, 1H), 1.92–1.80 (m, 4H), 1.77–1.68 (m, 1H), 1.67–1.55 (m, 2H), 1.42–1.23 (m, 3H); ^{13}C NMR (125 MHz, $CDCl_3$): δ 174.9, 143.4, 131.3, 127.7, 124.6, 123.6, 117.5, 47.9, 44.1, 29.2, 28.2, 26.0, 25.9; IR (film): 3075, 2920, 2851, 1649, 1598, 1482 cm^{-1}; HRMS-APCI (*m/z*) $[M + H]^+$ calcd for $C_{15}H_{20}NO$, 230.1545; found 230.1544.

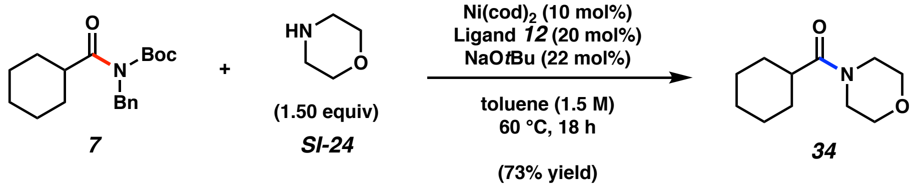

Amide 34 (Figure 4): Purification by preparative thin layer chromatography (9:1 → 3:1 → 1:1 Hexanes:EtOAc) generated amide **34** (73% yield, average of two experiments) as a white solid. Amide **34**: R_f 0.38 (2:1 Hexanes:EtOAc). Spectral data match those previously reported.[7]

E. Amine Nucleophile Competition Experiments

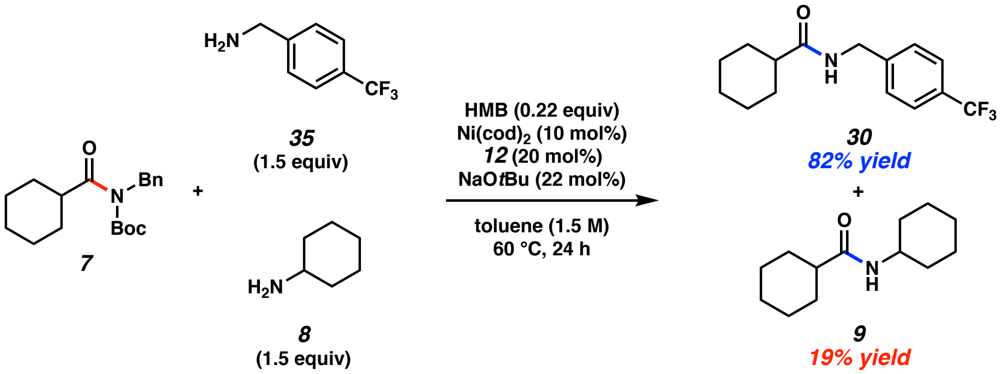

Unbranched vs α-Branched Primary Amine (Figure 5). A 1-dram vial containing a magnetic stir bar was flame-dried under reduced pressure, and then allowed to cool under N_2. The vial was charged with amide substrate **7** (63.4 mg, 0.200 mmol, 1.0 equiv) and the vial was flushed with N_2. The vial was then taken into a glove box and charged with 1,3-dicyclohexylbenzimidazolium chloride (**12**, 12.8 mg, 0.040 mmol, 20 mol%), Ni(cod)$_2$ (5.5 mg, 0.010 mmol, 10 mol%) and NaO*t*Bu (4.2 mg, 0.044 mmol, 22 mol%). Subsequently, cyclohexylamine (**8**, 31.4 μL, 0.30 mmol, 1.5 equiv), 4-(trifluoromethyl)benzylamine (**35**, 34.0 μL, 0.30 mmol, 1.5 equiv) and then toluene (0.133 mL, 1.5 M) were added. The vial was sealed with a Teflon-lined screw cap, removed from the glove box, and stirred at 60 °C for 24 h. After cooling to 23 °C, the mixture was diluted with hexanes (0.5 mL) and filtered over a plug of silica gel (10 mL of EtOAc eluent). The volatiles were removed under reduced pressure. Yields were determined by ^{1}H NMR analysis with hexamethylbenzene (HMB). Spectral data match those previously reported for amides **30** and **9** (see pages S20 and S14, respectively).

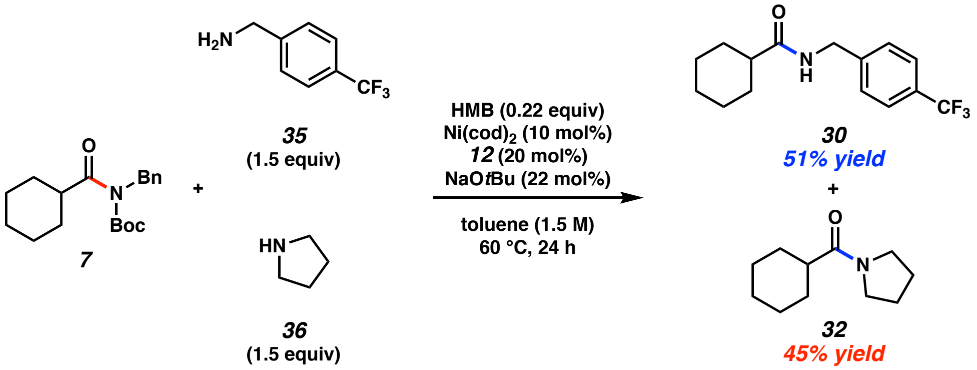

Unbranched Primary vs Secondary Amine (Figure 5). A 1-dram vial containing a magnetic stir bar was flame-dried under reduced pressure, and then allowed to cool under N_2. The vial was

charged with amide substrate **7** (63.4 mg, 0.200 mmol, 1.0 equiv) and the vial was flushed with N_2. The vial was then taken into a glove box and charged with 1,3-dicyclohexylbenzimidazolium chloride (**12**, 12.8 mg, 0.040 mmol, 20 mol%), $Ni(cod)_2$ (5.5 mg, 0.010 mmol, 10 mol%) and NaO*t*Bu (4.2 mg, 0.044 mmol, 22 mol%). Subsequently, 4-(trifluoromethyl)benzylamine (**35**, 34.0 μL, 0.30 mmol, 1.5 equiv), pyrrolidine (**36**, 25.0 μL, 0.30 mmol, 1.5 equiv) and then toluene (0.133 mL, 1.5 M) were added. The vial was sealed with a Teflon-lined screw cap, removed from the glove box, and stirred at 60 °C for 24 h. After cooling to 23 °C, the mixture was diluted with hexanes (0.5 mL) and filtered over a plug of silica gel (10 mL of EtOAc eluent). The volatiles were removed under reduced pressure. Yields were determined by ^{1}H NMR analysis with hexamethylbenzene (HMB). Spectral data match those previously reported for amides **30** and **32** (see pages S20 and S21 respectively).

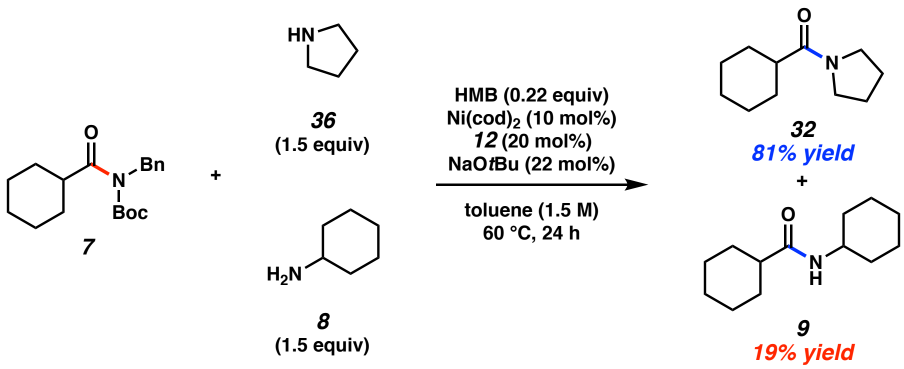

Secondary vs Primary α-Branched Amine (Figure 5). A 1-dram vial containing a magnetic stir bar was flame-dried under reduced pressure, and then allowed to cool under N_2. The vial was charged with amide substrate **7** (63.4 mg, 0.200 mmol, 1.0 equiv) and the vial was flushed with N_2. The vial was then taken into a glove box and charged with 1,3-dicyclohexylbenzimidazolium chloride (**12**, 12.8 mg, 0.040 mmol, 20 mol%), $Ni(cod)_2$ (5.5 mg, 0.010 mmol, 10 mol%) and NaO*t*Bu (4.2 mg, 0.044 mmol, 22 mol%). Subsequently, pyrrolidine (**36**, 25.0 μL, 0.30 mmol, 1.5 equiv), cyclohexylamine (**8,** 34.0 μL, 0.30 mmol, 1.5 equiv), and then toluene (0.133 mL, 1.5 M) were added. The vial was sealed with a Teflon-lined screw cap, removed from the glove box, and stirred at 60 °C for 24 h. After cooling to 23 °C, the mixture was diluted with hexanes (0.5 mL) and filtered over a plug of silica gel (10 mL of EtOAc eluent). The volatiles were removed under reduced pressure. Yields were determined by ^{1}H NMR analysis with hexamethylbenzene

(HMB). Spectral data match those previously reported for amides **32** and **9** (see pages S21 and S14 respectively).

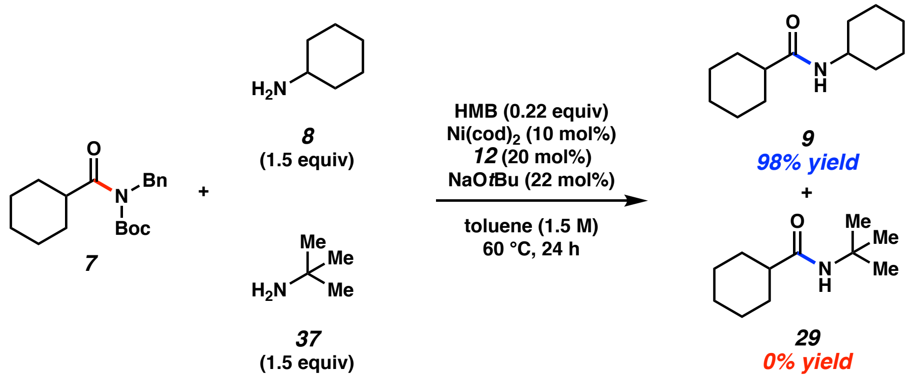

Cyclohexyl vs *t*-Butyl Amine (Figure 5). A 1-dram vial containing a magnetic stir bar was flame-dried under reduced pressure, and then allowed to cool under N_2. The vial was charged with amide substrate **7** (63.4 mg, 0.200 mmol, 1.0 equiv) and the vial was flushed with N_2. The vial was then taken into a glove box and charged with 1,3-dicyclohexylbenzimidazolium chloride (**12**, 12.8 mg, 0.040 mmol, 20 mol%), Ni(cod)$_2$ (5.5 mg, 0.010 mmol, 10 mol%) and NaO*t*Bu (4.2 mg, 0.044 mmol, 22 mol%). Subsequently, cyclohexylamine (**8**, 34.0 μL, 0.30 mmol, 1.5 equiv), *tert*-butylamine (**37**, 32.0 μL, 0.30 mmol, 1.5 equiv), and then toluene (0.133 mL, 1.5 M) were added. The vial was sealed with a Teflon-lined screw cap, removed from the glove box, and stirred at 60 °C for 24 h. After cooling to 23 °C, the mixture was diluted with hexanes (0.5 mL) and filtered over a plug of silica gel (10 mL of EtOAc eluent). The volatiles were removed under reduced pressure. Yields were determined by ^{1}H NMR analysis with hexamethylbenzene (HMB). Spectral data match those previously reported for amide **9** (see page S14).

F. Gram Scale Transamidation of Enantioenriched Aliphatic Amide 38

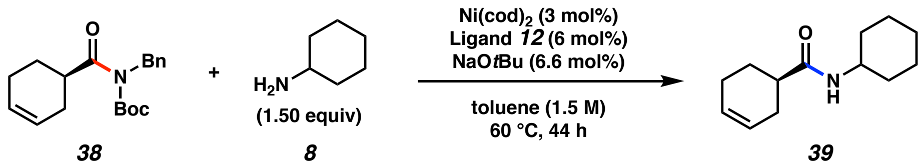

Amide 39 (Figure 6): A scintillation vial containing a magnetic stir bar was flame-dried under reduced pressure, and then allowed to cool under N_2. The vial was charged with amide substrate **38** (1.00 g, 3.18 mmol, 1.0 equiv) and the vial was flushed with N_2. The vial was then taken into a glove box and charged with 1,3-dicyclohexylbenzimidazolium chloride (**12**, 60.9 mg, 0.191 mmol, 6 mol%), Ni(cod)$_2$ (26.3 mg, 0.095 mmol, 3 mol%) and NaO*t*Bu (20.2 mg, 0.210 mmol, 6.6 mol%). Subsequently, cyclohexylamine (**8**, 547 μL, 4.77 mmol, 1.5 equiv) and then toluene (2.12 mL, 1.5 M) were added. The vial was sealed with a Teflon-lined screw cap, removed from the glove box, and stirred at 60 °C for 44 h. After cooling to 23 °C, the mixture was diluted with hexanes (0.50 mL) and filtered over a plug of silica gel (20 mL of EtOAc eluent). The volatiles were removed under reduced pressure and the crude residue was purified by flash chromatography (9:1 Hexanes:EtOAc) to generate amide **39** (78% yield) as a white solid. Amide **39**: mp: 160.5–162.3 °C; R_f 0.31 (2:1 Hexanes:EtOAc); ^{1}H NMR (500 MHz, $CDCl_3$): δ 5.74–5.65 (m, 2H), 5.35 (s, 1H), 3.78 (m, 1H), 2.35–2.02 (m, 5H), 1.96–1.86 (m, 3H), 1.77–1.65 (m, 3H), 1.65–1.58 (m, 1H), 1.42–1.32 (m, 2H), 1.22–1.06 (m, 3H); ^{13}C NMR (125 MHz, $CDCl_3$): δ 174.9, 127.0 125.6, 48.0, 41.6, 33.4, 28.3, 26.0, 25.7, 25.0, 24.8; IR (film): 3294, 2932, 1637, 1548, 1234 cm^{-1}; HRMS-APCI (*m/z*) $[M + H]^+$ calcd for $C_{13}H_{22}NO$, 208.1701; found 208.1686; $[\alpha]^{20.4}_D$ –42.40 ° (*c* = 1.00, $CHCl_3$).

G. Procedure for the Transamidation of 40 using Pre-Formed Catalyst Solution

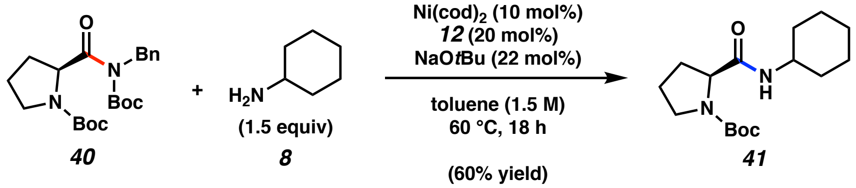

Amide 41 (Figure 6): Two 1-dram vials containing magnetic stir bars were flame-dried under reduced pressure, and then allowed to cool under N_2. One vial was charged with amide substrate **40** (80.9 mg, 0.200 mmol, 1.0 equiv) and then flushed with N_2. Both vials were taken into a glove box. In one of the vials, a stock solution of active catalyst was made by charging the empty 1-dram vial with 1,3-dicyclohexylbenzimidazolium chloride (**12**, 38.4 mg, 0.120 mmol, 60 mol%), Ni(cod)$_2$ (16.5 mg, 0.060 mmol, 30 mol%), NaO*t*Bu (12.6 mg, 0.132 mmol, 66 mol%), and toluene (0.399 mL, 0.5 M). The vial was sealed with a Teflon-lined screw cap, and stirred at ambient temperature in the glove box for 1 h. After 1 h, a portion of the catalyst stock solution (0.133 mL) and cyclohexylamine (**8**, 34.1 μL, 0.30 mmol, 1.5 equiv) were added to the vial containing substrate **40**. This vial was sealed with a Teflon-lined screw cap, removed from the glove box, and stirred at 60 °C for 18 h. After cooling to 23 °C, the mixture was diluted with hexanes (0.5 mL) and filtered over a plug of silica gel (10 mL of EtOAc eluent). The volatiles were removed under reduced pressure and the crude residue was purified by flash chromatography (10:1 → 3:1 Hexanes:EtOAc → 100% EtOAc) to generate amide **41** (60% yield, average of two experiments) as a white solid. Amide **41**: R_f 0.17 (2:1 Hexanes:EtOAc). Spectral data match those previously reported.[8]

H. Verification of Enantiopurity

a) Synthesis of Racemic Compound

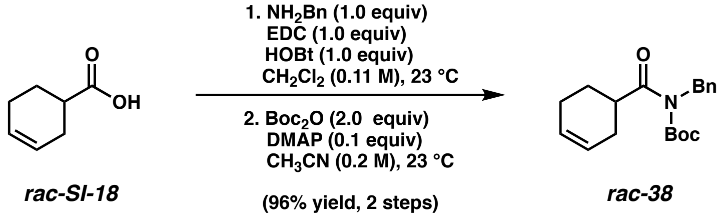

rac-SI-18 *rac-38*

Rac-38 Amide. To a mixture of carboxylic acid **rac-SI-18** (1.0 g, 7.9 mmol, 1.0 equiv), EDC (1.50 g, 7.9 mmol, 1.0 equiv), HOBt (1.1 g, 7.9 mmol, 1.0 equiv), and CH_2Cl_2 (79 mL, 0.1 M) was added benzylamine (0.86 mL, 7.9 mmol, 1.0 equiv). The resulting mixture was stirred at 23 °C for 16 h, and then diluted with CH_2Cl_2 (30 mL). The mixture was washed with 1 M HCl (30 mL), saturated aqueous $NaHCO_3$ (30 mL), and brine (30 mL). The combined organic layer was dried over Na_2SO_4, filtered, and then concentrated under reduced pressure. The resulting crude material was used in the subsequent step without further purification.

To a flask containing the crude material from the previous step was added DMAP (56.7 mg, 0.46 mmol, 0.1 equiv), followed by acetonitrile (23 mL, 0.2 M). Boc_2O (2.03 g, 9.28 mmol, 2.0 equiv) was added in one portion and the reaction vessel was flushed with N_2. The reaction mixture was allowed to stir at 23 °C for 12 h. The mixture was washed with 1.0 M HCl (5 mL) and brine (5 mL). After drying over Na_2SO_4, the organic layers were filtered and concentrated under reduced pressure. The resulting crude residue was purified by flash chromatography (10:1 Hexanes:EtOAc) to yield **rac-38** (1.40 g, 96% yield, over two steps) as a clear oil. Spectral data matched those previously reported for amide **38** (see page S11).

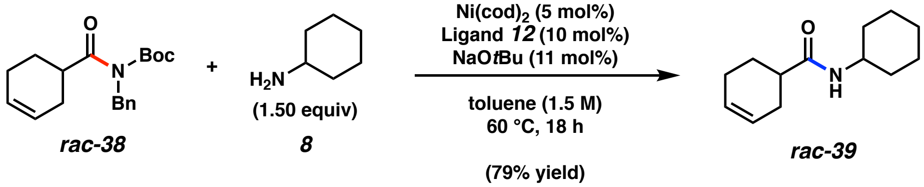

rac-38 *8* *rac-39*

Amide rac-39. A 1-dram vial containing a magnetic stir bar was flame-dried under reduced pressure, and then allowed to cool under N_2. The vial was charged with amide substrate **rac-38** (63.1 mg, 0.200 mmol, 1.0 equiv) and the vial was flushed with N_2. The vial was then taken into a glove box and charged with 1,3-dicyclohexylbenzimidazolium chloride (**12**, 6.4 mg, 0.020

mmol, 10 mol%), Ni(cod)$_2$ (2.8 mg, 0.010 mmol, 5 mol%) and NaO*t*Bu (2.1 mg, 0.022 mmol, 11 mol%). Subsequently, cyclohexylamine (**8**, 34.0 μL, 0.30 mmol, 1.5 equiv) and then toluene (0.133 mL, 1.5 M) were added. The vial was sealed with a Teflon-lined screw cap, removed from the glove box, and stirred at 60 °C for 18 h. After cooling to 23 °C, the mixture was diluted with hexanes (0.5 mL) and filtered over a plug of silica gel (10 mL of EtOAc eluent). The volatiles were removed under reduced pressure and the crude residue was purified by flash chromatography (9:1 → 3:1 Hexanes:EtOAc) to generate amide **rac-39** (79% yield, average of two experiments) as a white solid. Spectral data matched those previously reported for amide **39** (page S24).

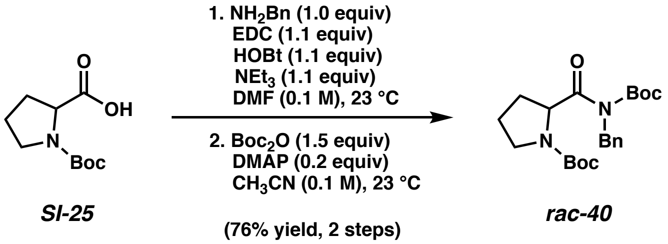

SI-25 *rac-40*

Amide rac-40 (Figure 6). rac-40 was prepared using the known procedure described for (–)-**40**,[1] but using racemic carboxylic acid **SI-25**. To a mixture of carboxylic acid **SI-25** (500 mg, 2.32 mmol, 1.0 equiv), EDC (490 mg, 2.55 mmol, 1.1 equiv), HOBt (344 mg, 2.55 mmol, 1.1 equiv), triethylamine (0.356 mL, 2.55 mmol, 1.1 equiv), and DMF (23 mL, 0.1 M) was added benzylamine (0.279 mL, 2.55 mmol, 1.1 equiv). The resulting mixture was stirred at 23 °C for 4 h, and then diluted with deionized water (30 mL) and EtOAc (30 mL). The layers were separated and the organic layer was washed successively with 1.0 M HCl (30 mL), saturated aqueous $NaHCO_3$ (30 mL), and brine (30 mL). The organic layer was dried over Na_2SO_4 and concentrated under reduced pressure. The resulting crude solid material was used in the subsequent step without further purification.

To a flask containing the crude material from the previous step was added DMAP (28 mg, 0.232 mmol, 0.2 equiv), followed by acetonitrile (23 mL, 0.1 M). Boc$_2$O (0.759 mg, 3.50 mmol, 1.5 equiv) was added in one portion and the reaction vessel was flushed with N_2. The reaction mixture was allowed to stir at 23 °C for 15 h. The reaction mixture was diluted with EtOAc (30 mL) and then washed sequentially with saturated aqueous $NaHCO_3$ (2 x 30 mL) and brine (30 mL). The organic layer was dried over Na_2SO_4 and concentrated under reduced pressure. The resulting crude residue was purified by flash chromatography (9:1 → 3:1

Hexanes:EtOAc) to yield amide **rac-40** (484 mg, 76% yield, over two steps) as a yellow oil. Spectral data matched those previously reported.[1]

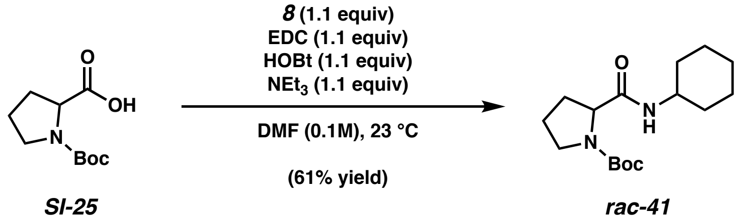

Amide rac-41. To a mixture of racemic carboxylic acid **SI-25** (250 mg, 1.16 mmol, 1.0 equiv), EDC (246 mg, 1.28 mmol, 1.1 equiv), HOBt (173 mg, 1.28 mmol, 1.1 equiv), triethylamine (0.178 mL, 1.28 mmol, 1.1 equiv), and DMF (12 mL, 0.1 M) was added cyclohexylamine (**8**) (0.147 mL, 1.28 mmol, 1.1 equiv). The resulting mixture was stirred at 23 °C for 3 h, and then diluted with deionized water (20 mL) and EtOAc (20 mL). The layers were separated and the organic layer was washed successively with 1.0 M HCl (20 mL), saturated aqueous $NaHCO_3$ (20 mL), and brine (20 mL). The organics were dried over Na_2SO_4 and concentrated under reduced pressure. The resulting crude residue was purified by flash chromatography (9:1 → 4:1 Hexanes:EtOAc → 100% EtOAc) to yield amide **rac-41** (206 mg, 61% yield) as a white solid. Spectral data matched those previously reported.[8]

b) Chiral SFC/HPLC Assays

Compound	Method Column/Temp.	Solvent	Method Flow Rate	Retention Times (min)	Enantiomeric Ratio (er)
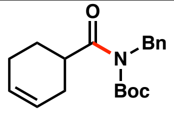 *rac-38*	Daicel ChiralPak OJ-H/23 °C	1% isopropanol in CO_2	1 mL/min	9.36/10.35	50:50
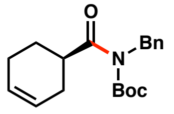 *38*	Daicel ChiralPak OJ-H/23 °C	1% isopropanol in CO_2	1 mL/min	9.16/10.33	99:1

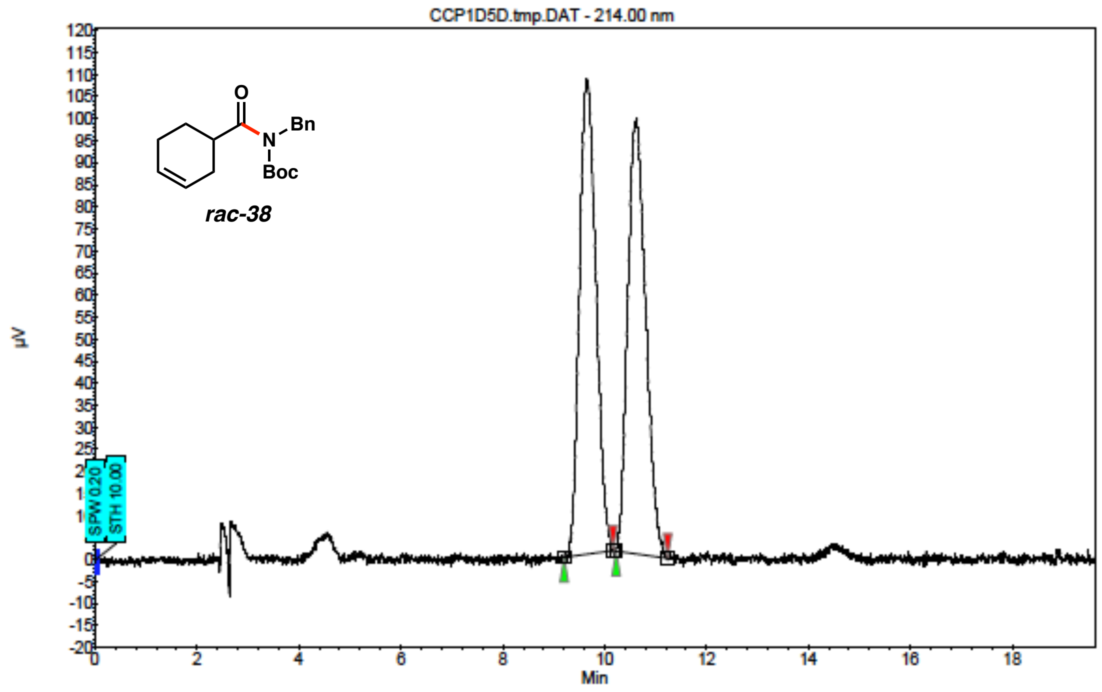

Index	Name	Start	Time	End	RT Offset	Quantity	Height	Area	Area
		[Min]	[Min]	[Min]	[Min]	[% Area]	[µV]	[µV.Min]	[%]
1	UNKNOWN	9.21	9.64	10.16	0.00	50.12	107.8	40.6	50.117
2	UNKNOWN	10.22	10.63	11.24	0.00	49.88	98.8	40.4	49.883
Total						100.00	206.6	81.0	100.000

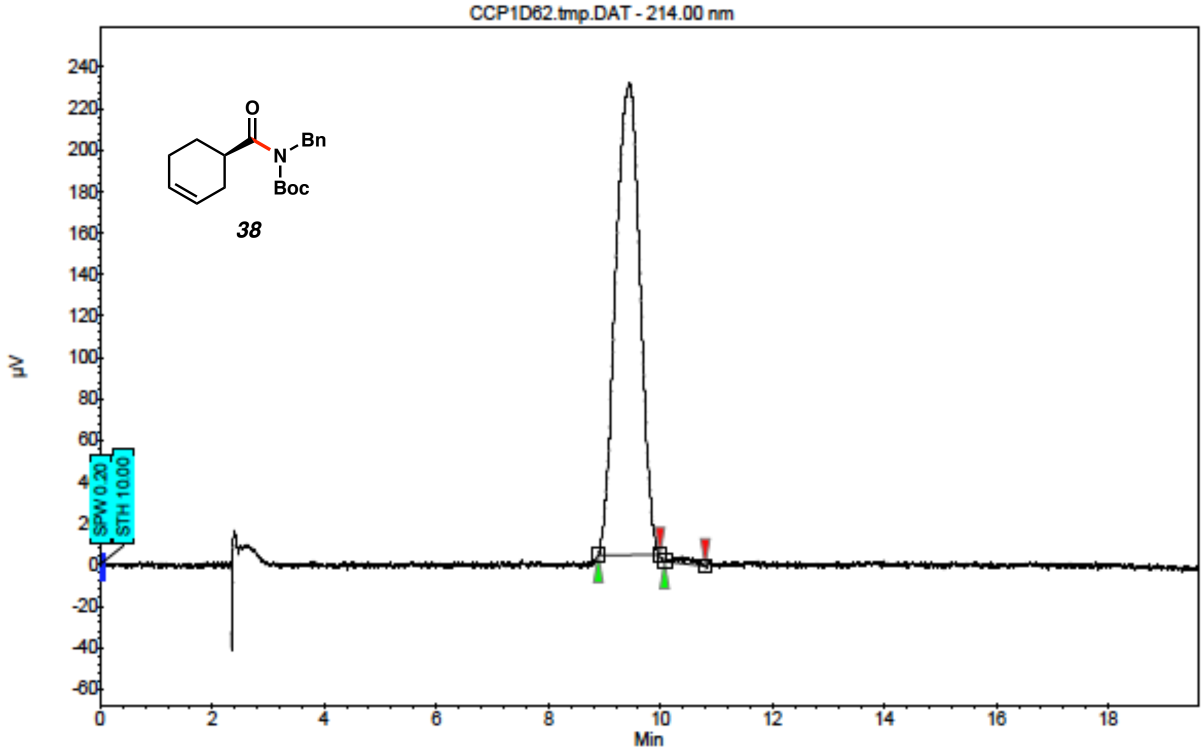

Index	Name	Start	Time	End	RT Offset	Quantity	Height	Area	Area
		[Min]	[Min]	[Min]	[Min]	[% Area]	[µV]	[µV.Min]	[%]
1	UNKNOWN	8.89	9.46	9.99	0.00	99.02	227.6	115.1	99.024
2	UNKNOWN	10.07	10.39	10.80	0.00	0.98	3.5	1.1	0.976
Total						100.00	231.2	116.2	100.000

Compound	Method Column/Temp.	Solvent	Method Flow Rate	Retention Times (min)	Enantiomeric Ratio (er)
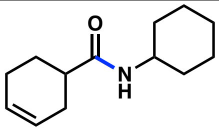 ***rac*-39**	Daicel ChiralPak IC-H/23 °C	10% isopropanol in hexanes	1 mL/min	11.4/12.7	50:50
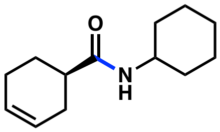 ***39***	Daicel ChiralPak IC-H/23 °C	10% isopropanol in hexanes	1 mL/min	11.5/12.7	95:5

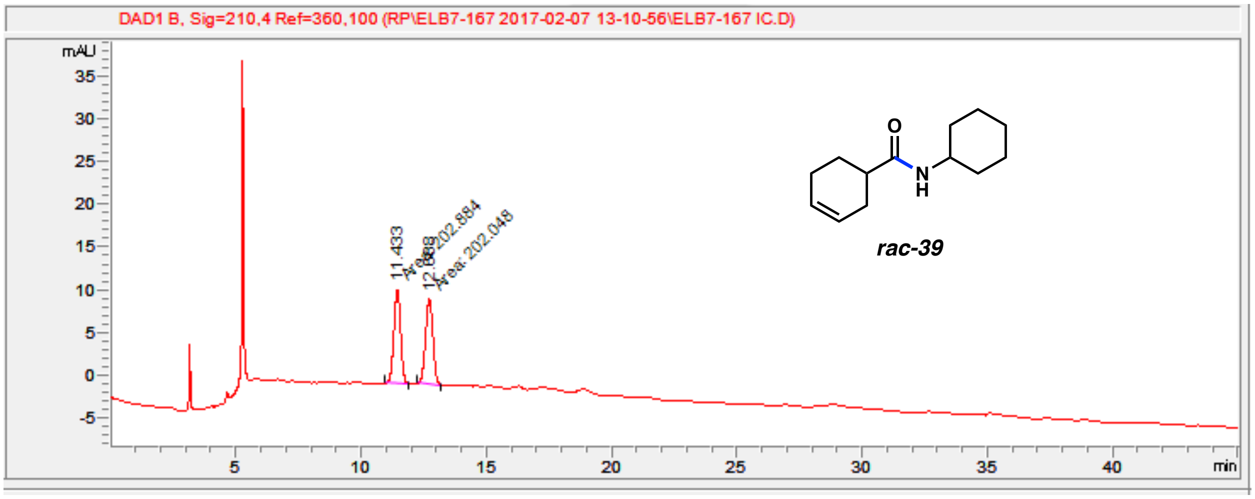

#	Time	Area	Height	Width	Area%	Symmetry
1	11.433	202.9	10.9	0.3097	50.103	1.042
2	12.688	202	10	0.3383	49.897	1.032

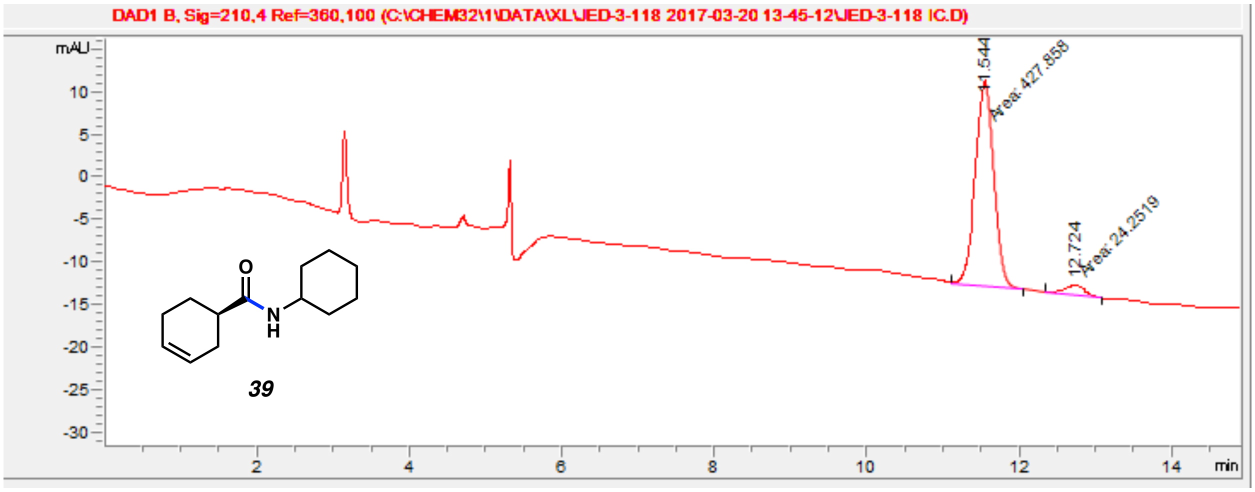

#	Time	Area	Height	Width	Area%	Symmetry
1	11.544	427.9	24.1	0.2957	94.636	0.997
2	12.724	24.3	1.2	0.3383	5.364	0.902

Compound	Method Column/Temp.	Solvent	Method Flow Rate	Retention Times (min)	Enantiomeric Ratio (er)
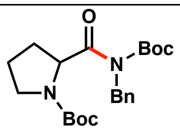 *rac-40*	Daicel ChiralPak IC-H/23 °C	10% isopropanol in hexanes	1 mL/min	13.3/15.6	50:50
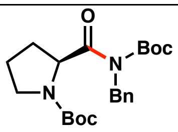 *40*	Daicel ChiralPak IC-H/23 °C	10% isopropanol in hexanes	1 mL/min	14.5	>99:1

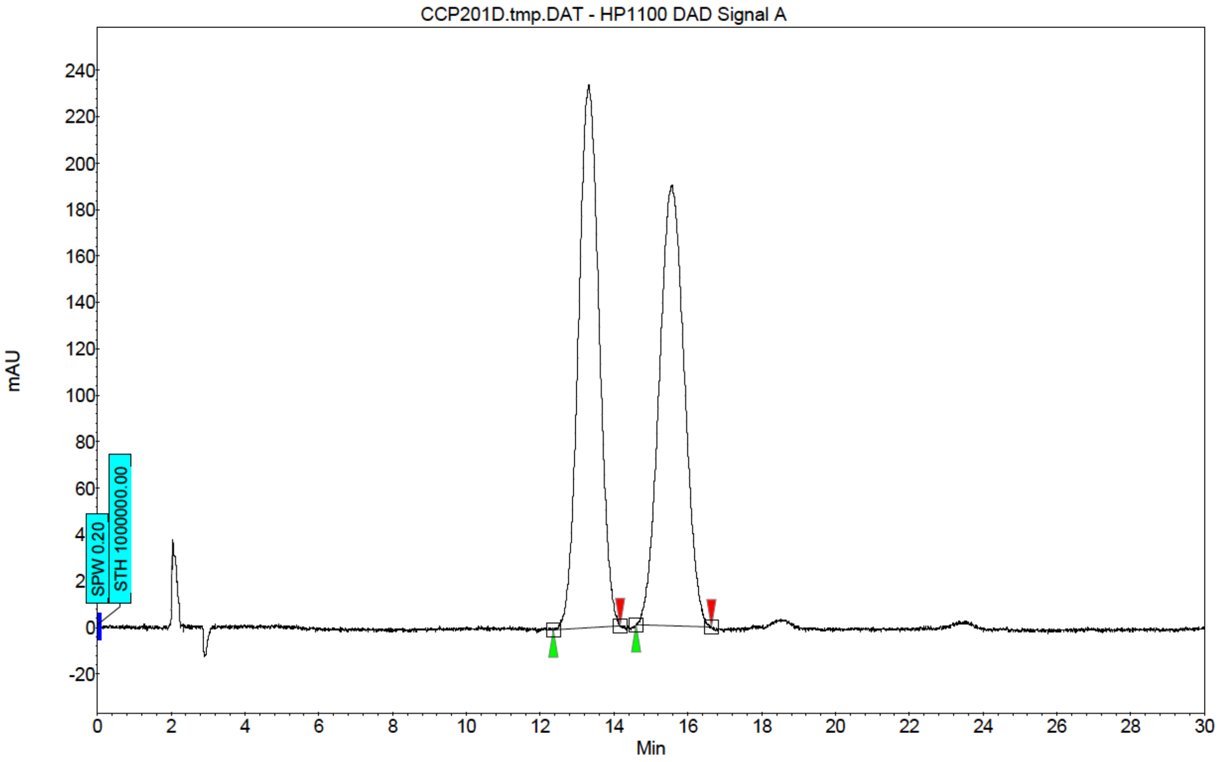

Index	Name	Start	Time	End	RT Offset	Quantity	Height	Area	Area
		[Min]	[Min]	[Min]	[Min]	[% Area]	[µV]	[µV.Min]	[%]
1	UNKNOWN	12.35	13.31	14.16	0.00	50.27	233.8	151.6	50.268
2	UNKNOWN	14.59	15.57	16.63	0.00	49.73	190.0	149.9	49.732
Total						100.00	423.8	301.5	100.000

Compound	Method Column/Temp.	Solvent	Method Flow Rate	Retention Times (min)	Enantiomeric Ratio (er)
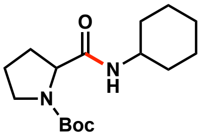 ***rac*-41**	Daicel ChiralPak AD-H/23 °C	10% isopropanol in hexanes	1 mL/min	3.9/5.5	50:50
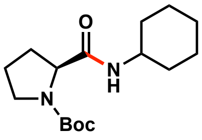 ***41***	Daicel ChiralPak AD-H/23 °C	10% isopropanol in hexanes	1 mL/min	5.7	>99:1

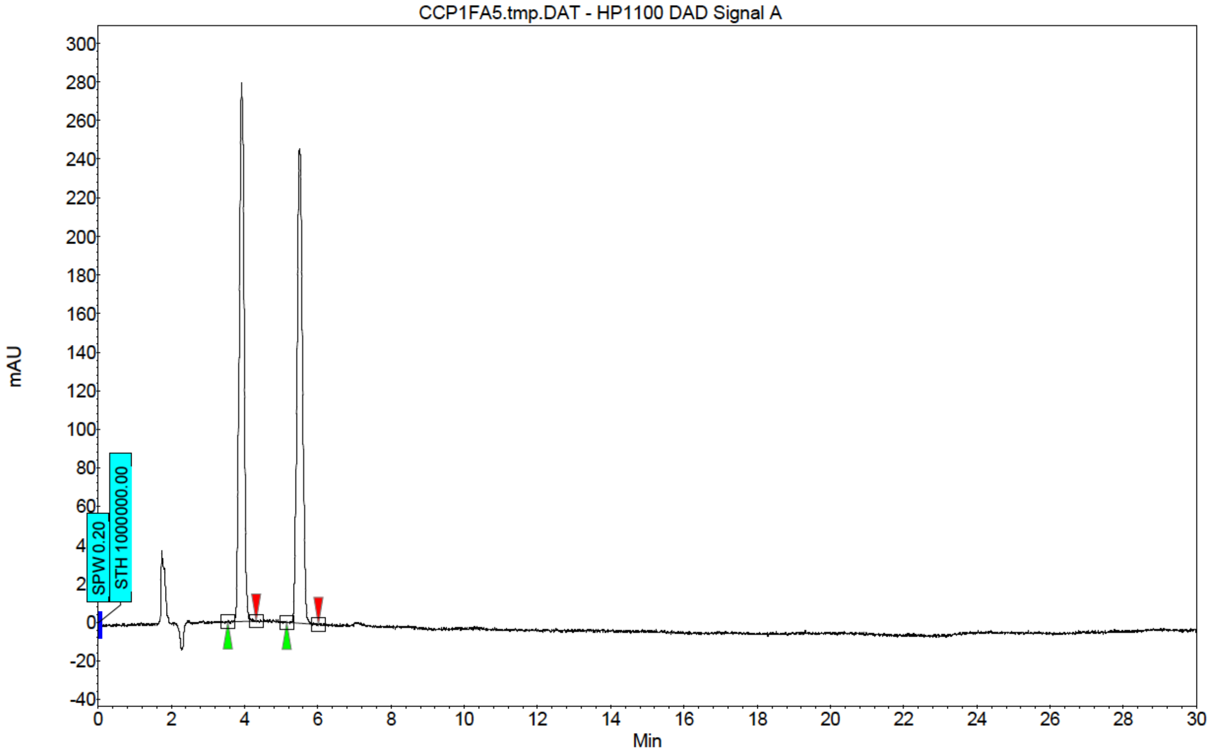

Index	Name	Start	Time	End	RT Offset	Quantity	Height	Area	Area
		[Min]	[Min]	[Min]	[Min]	[% Area]	[µV]	[µV.Min]	[%]
1	UNKNOWN	3.54	3.92	4.31	0.00	50.05	279.3	42.5	50.049
2	UNKNOWN	5.15	5.50	6.02	0.00	49.95	246.1	42.4	49.951
Total						100.00	525.3	85.0	100.000

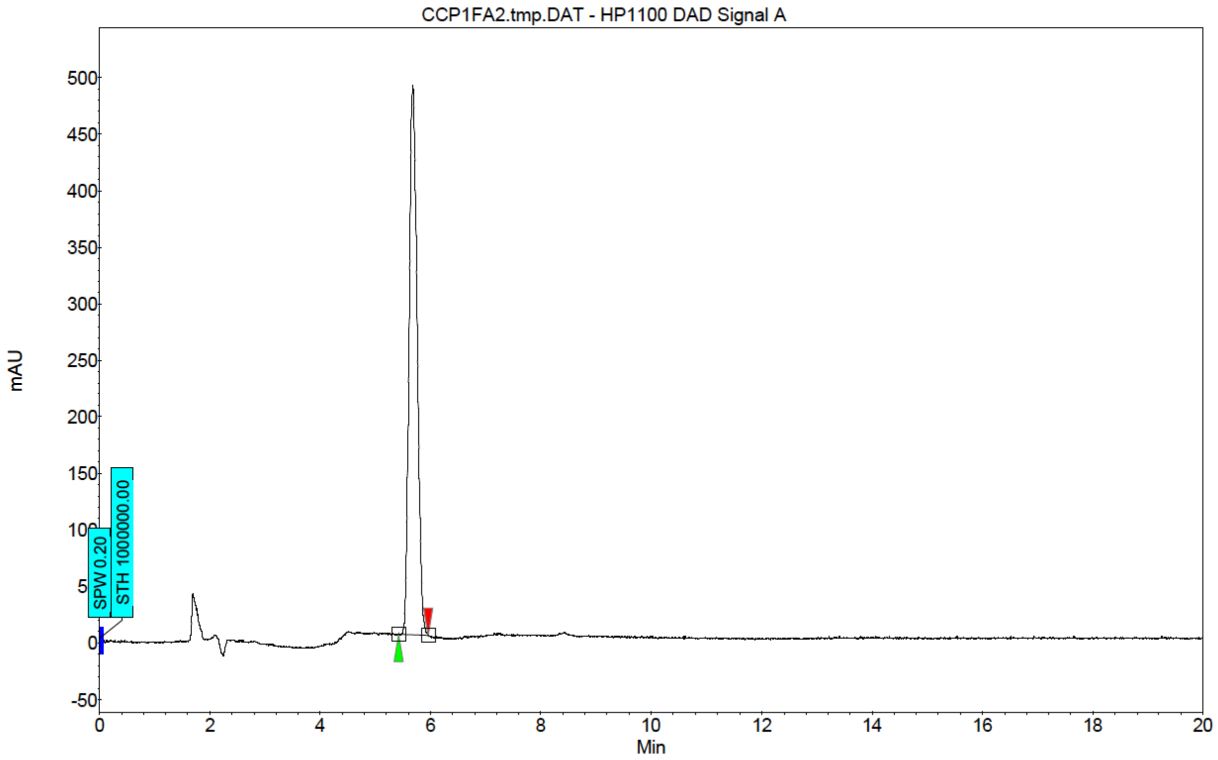

Index	Name	Start	Time	End	RT Offset	Quantity	Height	Area	Area
		[Min]	[Min]	[Min]	[Min]	[% Area]	[µV]	[µV.Min]	[%]
1	UNKNOWN	5.42	5.68	5.96	0.00	100.00	486.2	82.3	100.000
Total						100.00	486.2	82.3	100.000

I. Lewis Base-Catalyzed Transamidation Control Experiments

a) Representative procedure for Lewis base-catalyzed transamidation experiments (coupling of amide 38 and cyclohexylamine (8) is used as an example). A 1-dram vial containing a magnetic stir bar was flame-dried under reduced pressure, and then allowed to cool under N_2. The vial was charged with amide substrate **38** (63.0 mg, 0.2 mmol, 1.0 equiv) and the vial was flushed with N_2. To the vial were added dichloromethane (200 µL, 1.0 M), NEt_3 (84 µL, 0.6 mmol, 3.0 equiv), and cyclohexylamine (**8**, 69 µL, 0.6 mmol, 3.0 equiv) sequentially at 23 °C with vigorous stirring. The vial was then sealed with a Teflon-lined screw cap under a positive stream of N_2 and allowed to stir at 23 °C for 15 h. After this time, the reaction was diluted with CH_2Cl_2 (2 mL) and filtered over a celite plug. The mixture was then washed with 1 N HCl (1 mL) and brine (1 mL) sequentially. The organic layer was then dried over Na_2SO_4, subsequently

filtered, and the volatiles were evaporated under reduced pressure. The yield was determined by ^{1}H NMR analysis with hexamethylbenzene (HMB) as an external standard.

Any modifications of the conditions shown in the representative procedure above are specified in Table S2 below.

Table S2. Lewis Base-Catalyzed Transamidations of Aliphatic Amides.[a]

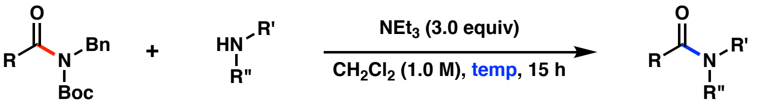

Entry	*Amide*	*Amine*	*Temp*	*Recovery of SM*	*Amide Product*
1	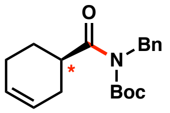 ***38***	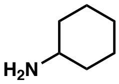 ***8***	23 °C	97% (98% e.e.)	0%
2	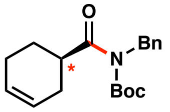 ***38***	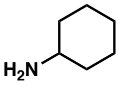 ***8***	60 °C	93% (99% e.e.)	trace
3	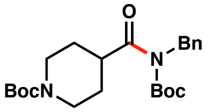 ***SI-8***	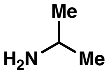 ***SI-15***	23 °C	95%	0%
4	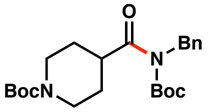 ***SI-8***	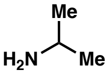 ***SI-15***	60 °C	97%	0%
5	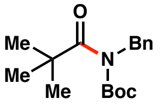 ***SI-2***	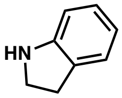 ***SI-23***	23 °C	Quant.	0%
6	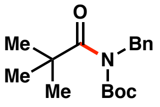 ***SI-2***	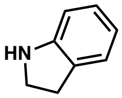 ***SI-23***	60 °C	Quant.	0%

[a] Yields were determined using ^{1}H NMR analysis with hexamethylbenzene (HMB) as an external standard.

b) Verification of enantiopurity for entries 1 and 2 in Table S2 (Chiral SFC/HPLC Assays)

Compound	Method Column/Temp.	Solvent	Method Flow Rate	Retention Times (min)	Enantiomeric Ratio (er)
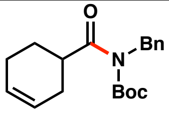 *rac-38*	Daicel ChiralPak OJ-H/23 °C	1% isopropanol in CO_2	1 mL/min	9.4/10.4	50:50
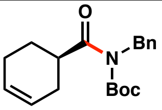 *38 (SM, entries 1–2, Table S2)*	Daicel ChiralPak OJ-H/23 °C	1% isopropanol in CO_2	1 mL/min	9.2/10.3	99:1
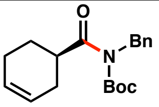 *38 (recovered, entry 1, Table S2)*	Daicel ChiralPak OJ-H/23 °C	1% isopropanol in CO_2	1 mL/min	9.2/10.3	99:1
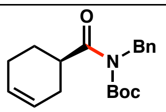 *38 (recovered, entry 2, Table S2)*	Daicel ChiralPak OJ-H/23 °C	1% isopropanol in CO_2	1 mL/min	9.2/10.3	>99:1

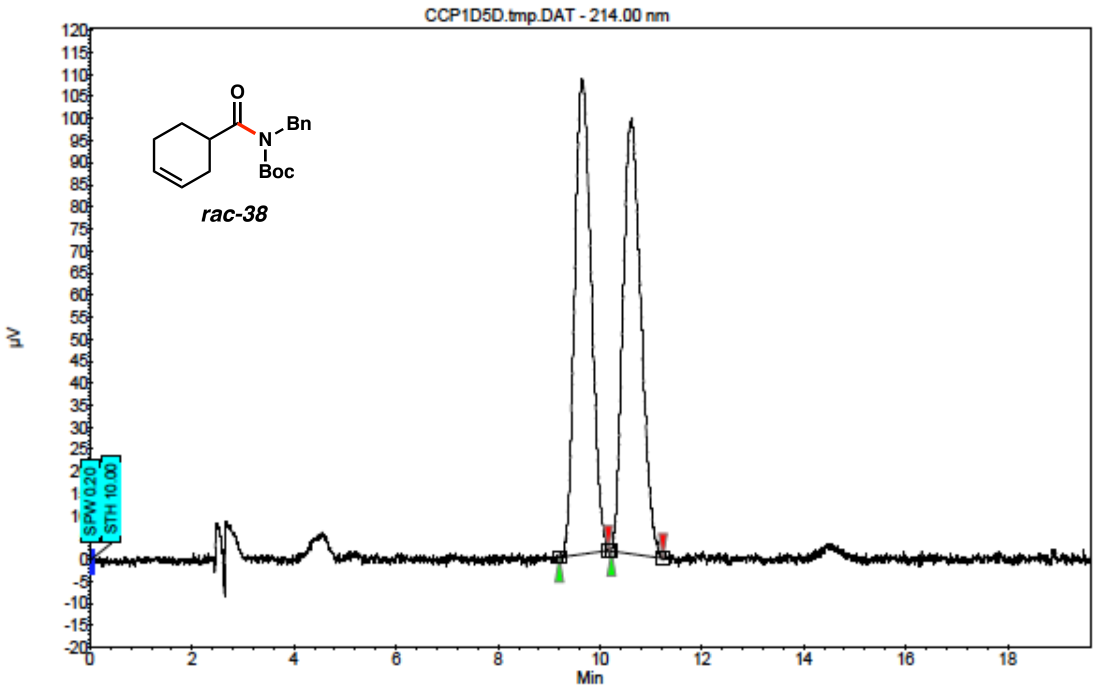

Index	Name	Start	Time	End	RT Offset	Quantity	Height	Area	Area
		[Min]	[Min]	[Min]	[Min]	[% Area]	[μV]	[μV.Min]	[%]
1	UNKNOWN	9.21	9.64	10.16	0.00	50.12	107.8	40.6	50.117
2	UNKNOWN	10.22	10.63	11.24	0.00	49.88	98.8	40.4	49.883
Total						100.00	206.6	81.0	100.000

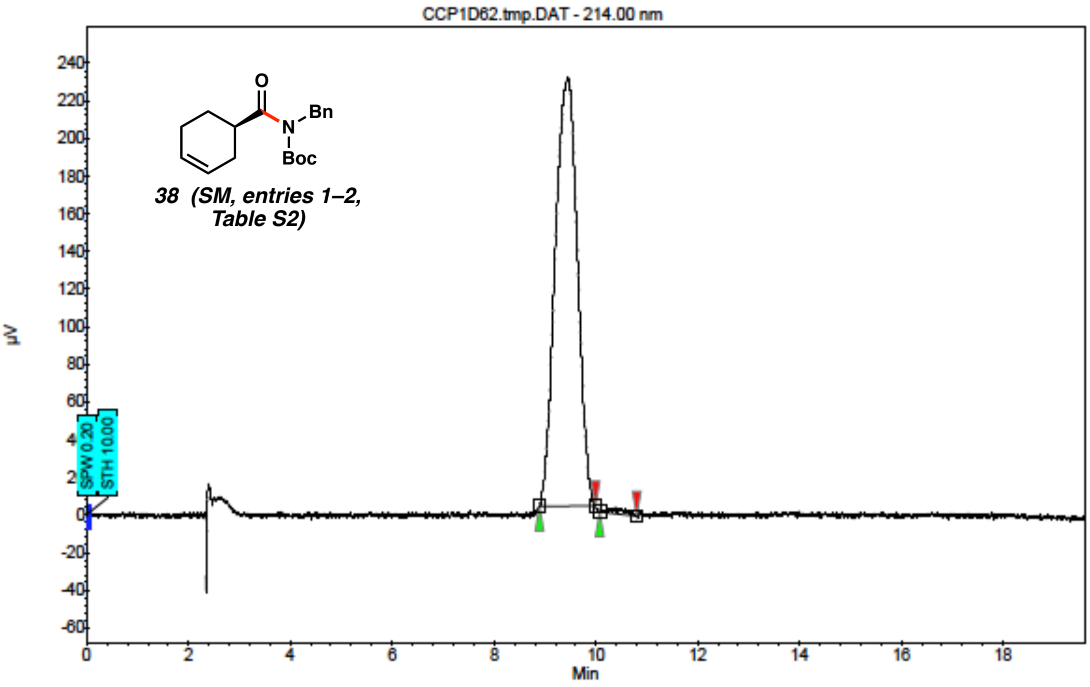

Index	Name	Start	Time	End	RT Offset	Quantity	Height	Area	Area
		[Min]	[Min]	[Min]	[Min]	[% Area]	[µV]	[µV.Min]	[%]
1	UNKNOWN	8.89	9.46	9.99	0.00	99.02	227.6	115.1	99.024
2	UNKNOWN	10.07	10.39	10.80	0.00	0.98	3.5	1.1	0.976
Total						100.00	231.2	116.2	100.000

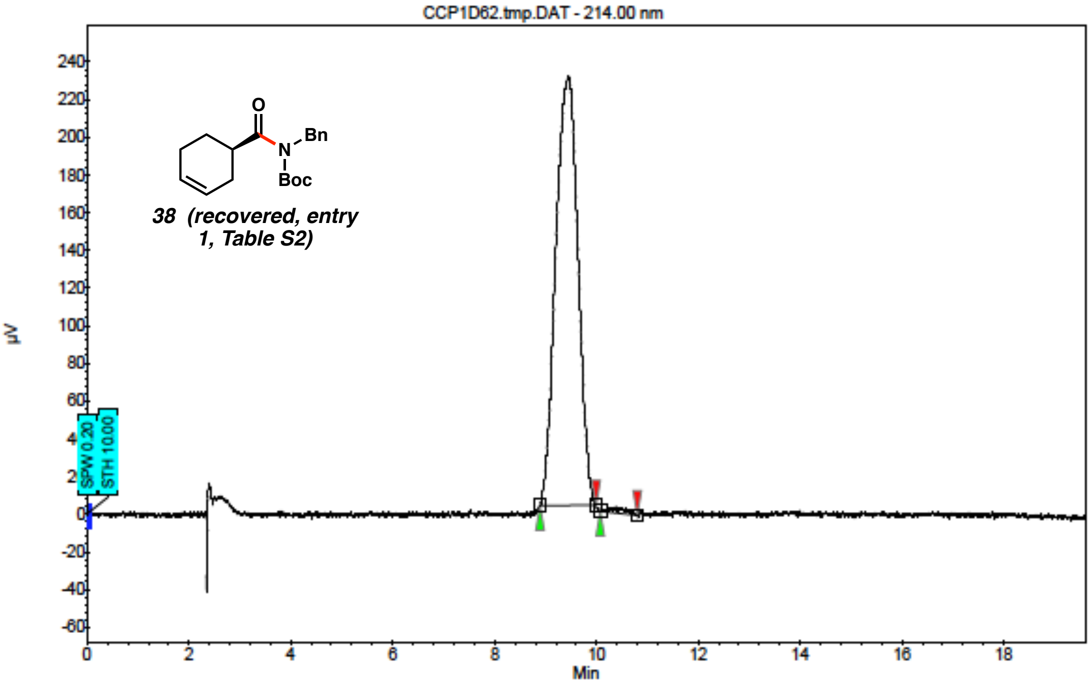

Index	Name	Start	Time	End	RT Offset	Quantity	Height	Area	Area
		[Min]	[Min]	[Min]	[Min]	[% Area]	[µV]	[µV.Min]	[%]
1	UNKNOWN	8.89	9.46	9.99	0.00	99.02	227.6	115.1	99.024
2	UNKNOWN	10.07	10.39	10.80	0.00	0.98	3.5	1.1	0.976
Total						100.00	231.2	116.2	100.000

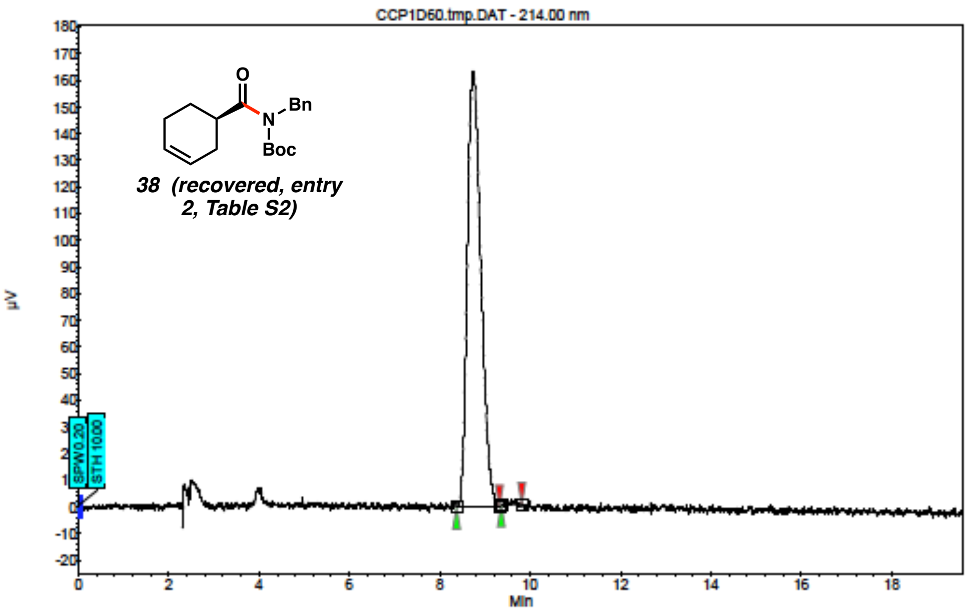

Index	Name	Start	Time	End	RT Offset	Quantity	Height	Area	Area
		[Min]	[Min]	[Min]	[Min]	[% Area]	[µV]	[µV.Min]	[%]
1	UNKNOWN	8.37	8.74	9.32	0.00	99.61	163.6	57.0	99.609
2	UNKNOWN	9.36	9.67	9.81	0.00	0.39	1.7	0.2	0.391
Total						100.00	165.3	57.2	100.000

References

[1] Hie, L.; Baker, E. L.; Anthony, S. M.; Desrosiers, J.-N.; Senanayake, C.; Garg, N. K *Angew. Chem., Int. Ed.* **2016**, *55*, 15129–15132.
[2] Lorenc, C.; Reeves, J. T.; Busacca, C. A.; Senanayake, C. H. *Tetrahedron Lett.* **2015**, *56*, 1280–1282.
[3] Serrano, E.; Martin, R. *Angew. Chem., Int. Ed.* **2016**, *55*, 11207–11211.
[4] Li, J.; Xu, F.; Zhang, Y.; Shen, Q. *J. Org. Chem.* **2009**, *74*, 2575–2577.
[5] Gu, L.; Wang, W.; Liu, J.; Yuan, M. *Green Chem.* **2016**, *18*, 2604–2608.
[6] Qian, C.; Zhang, X.; Zhang, Y.; Shen, Q. *J. Organomet. Chem.* **2010**, *695*, 747–752.
[7] Miyamura, H.; Min, H.; Soulé, J.-F.; Kobayashi, S. *Angew. Chem., Int. Ed.* **2015**, *54*, 7564–7567.
[8] McNulty, J.; Vemula, R.; Krishnamoorthy, V.; Robertson, A. *Angew. Chem., Int. Ed.* **2012**, *68*, 5415–5421.

^{1}H NMR Spectra

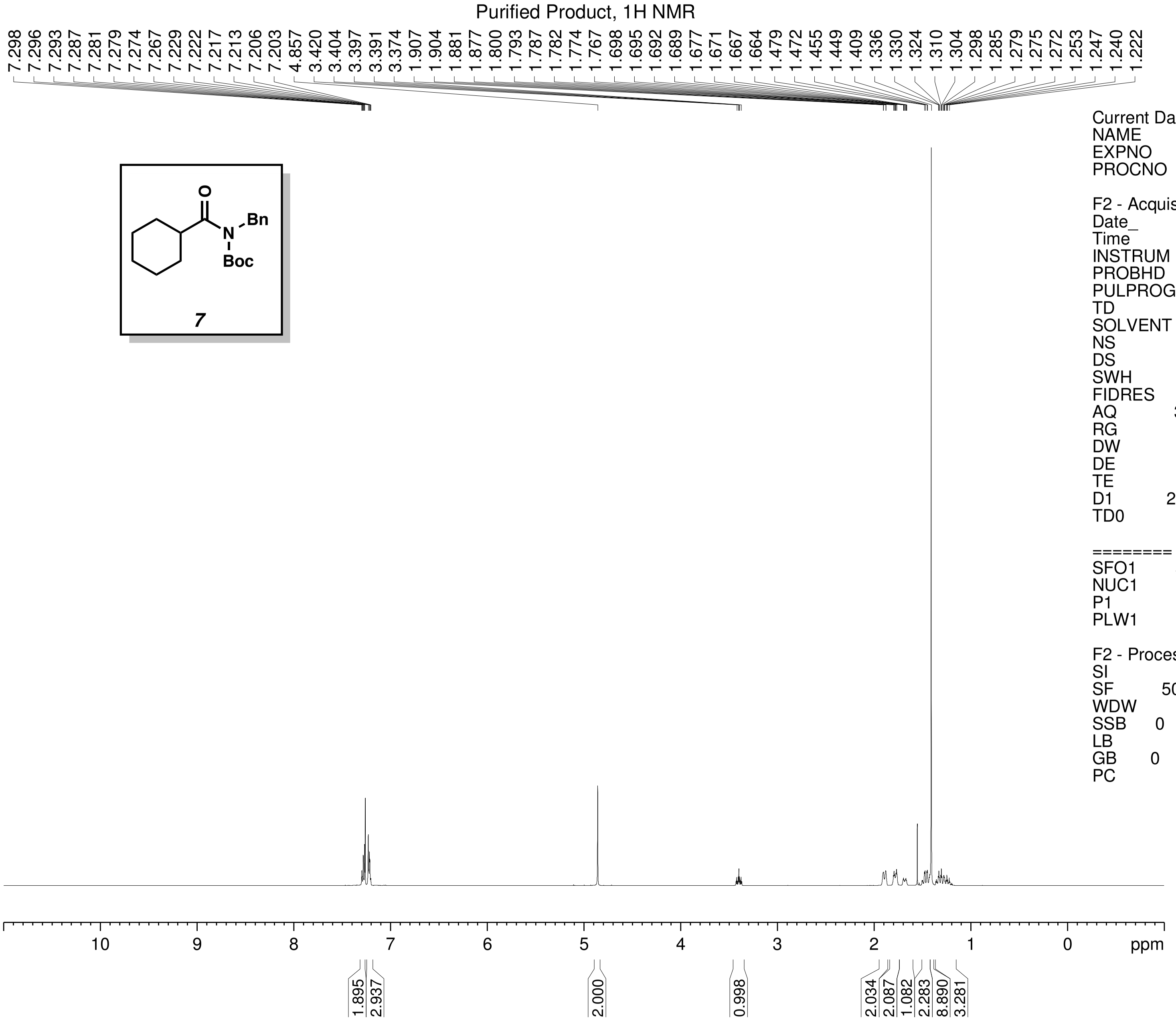

Current Data Parameters
NAME ELB-7-020p
EXPNO 2
PROCNO 1

F2 - Acquisition Parameters
Date_ 20161009
Time 9.24
INSTRUM av500
PROBHD 5 mm DCH 13C-1
PULPROG zg30
TD 65536
SOLVENT CDCl3
NS 8
DS 0
SWH 10000.000 Hz
FIDRES 0.152588 Hz
AQ 3.2767999 sec
RG 12.14
DW 50.000 usec
DE 10.00 usec
TE 298.0 K
D1 2.00000000 sec
TD0 1

======== CHANNEL f1 =====
SFO1 500.1330008 MHz
NUC1 1H
P1 10.00 usec
PLW1 13.50000000 W

F2 - Processing parameters
SI 65536
SF 500.1300122 MHz
WDW EM
SSB 0
LB 0.30 Hz
GB 0
PC 1.00

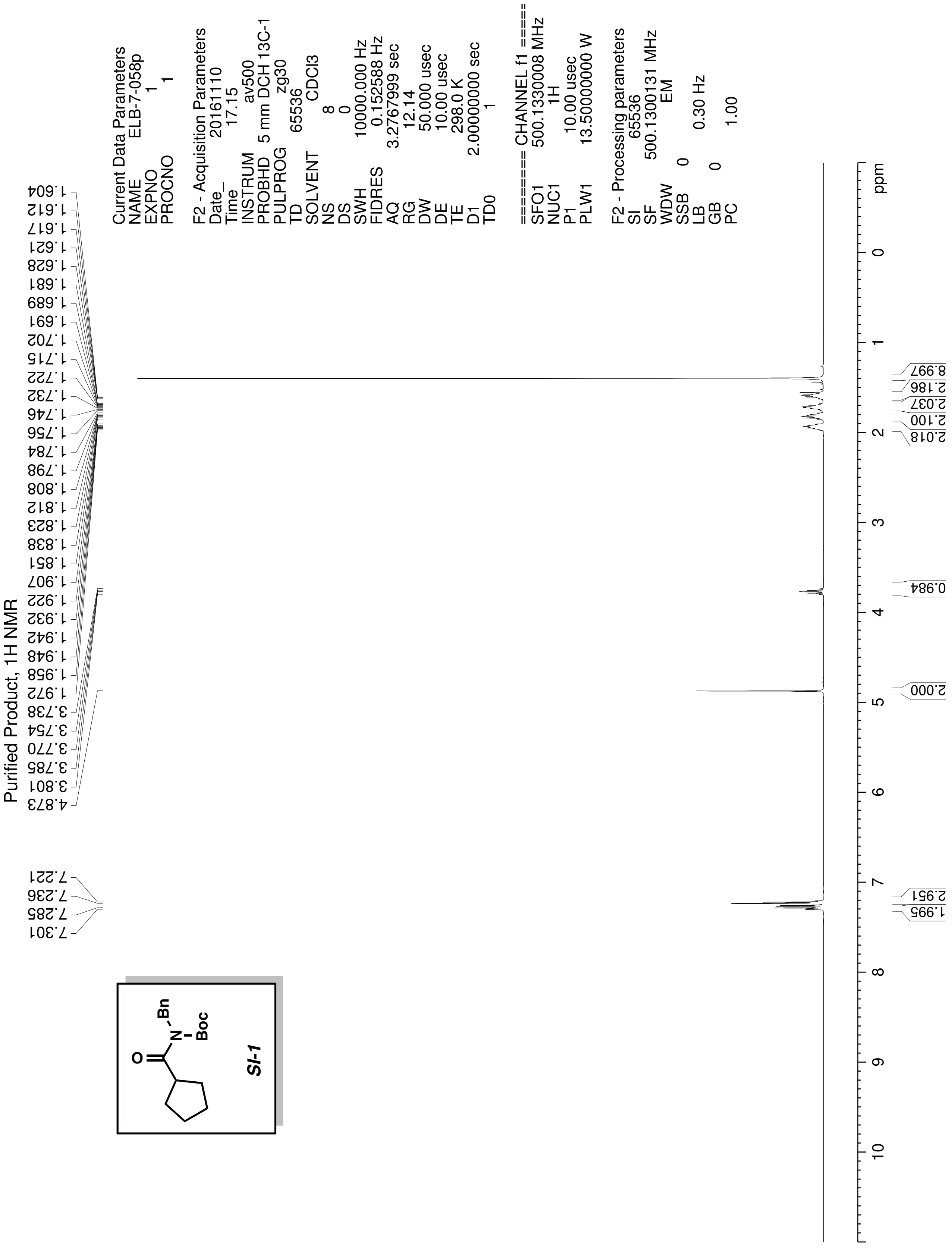
Purified Product, 1H NMR
7.301
7.285
7.236
7.221
4.873
3.801
3.785
3.770
3.754
3.738
1.972
1.958
1.948
1.942
1.932
1.922
1.907
1.851
1.838
1.823
1.812
1.808
1.798
1.784
1.756
1.746
1.732
1.722
1.715
1.702
1.691
1.689
1.681
1.628
1.621
1.617
1.612
1.604
O
N
Bn
Boc
SI-1
Current Data Parameters
NAME ELB-7-058p
EXPNO 1
PROCNO 1
F2 - Acquisition Parameters
Date_ 20161110
Time 17.15
INSTRUM av500
PROBHD 5 mm DCH 13C-1
PULPROG zg30
TD 65536
SOLVENT CDCl3
NS 8
DS 0
SWH 10000.000 Hz
FIDRES 0.152588 Hz
AQ 3.2767999 sec
RG 12.14
DW 50.000 usec
DE 10.00 usec
TE 298.0 K
D1 2.00000000 sec
TD0 1
======== CHANNEL f1 ========
SFO1 500.1330008 MHz
NUC1 1H
P1 10.00 usec
PLW1 13.50000000 W
F2 - Processing parameters
SI 65536
SF 500.1300131 MHz
WDW EM
SSB 0
LB 0.30 Hz
GB 0
PC 1.00
1.995
2.951
2.000
0.984
2.018
2.100
2.037
2.186
8.997
10
9
8
7
6
5
4
3
2
1
0
ppm

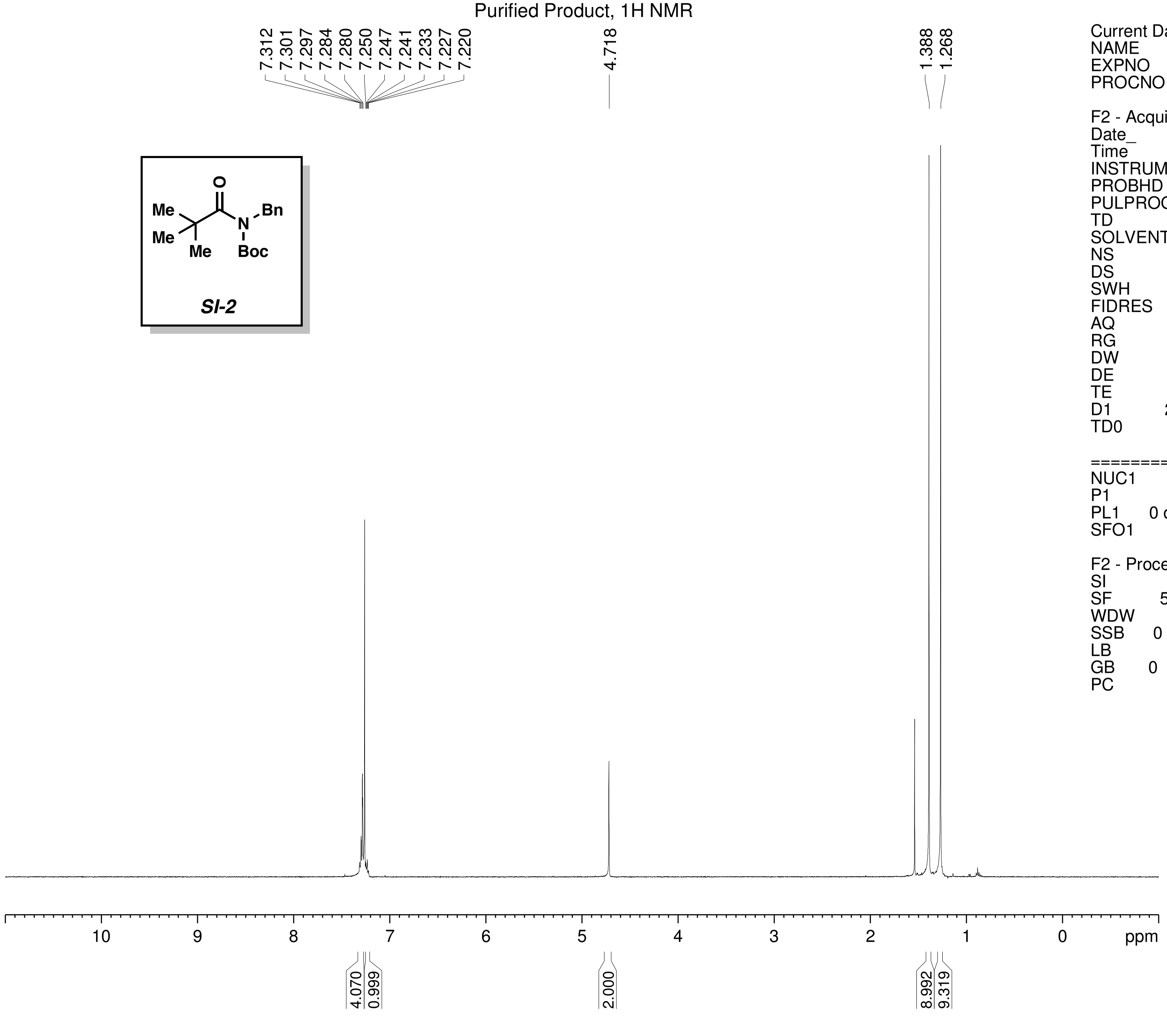

Current Data Parameters
NAME TBB-1-086p
EXPNO 1
PROCNO 1

F2 - Acquisition Parameters
Date_ 20160819
Time 9.43
INSTRUM drx500
PROBHD 5 mm bb-Z Z800
PULPROG zg30
TD 65536
SOLVENT CDCl3
NS 8
DS 0
SWH 10000.000 Hz
FIDRES 0.152588 Hz
AQ 3.2767999 sec
RG 203.2
DW 50.000 usec
DE 6.00 usec
TE 297.2 K
D1 2.00000000 sec
TD0 1

======== CHANNEL f1 =====
NUC1 1H
P1 12.25 usec
PL1 0 dB
SFO1 500.3330020 MHz

F2 - Processing parameters
SI 32768
SF 500.3300222 MHz
WDW EM
SSB 0
LB 0.30 Hz
GB 0
PC 1.00

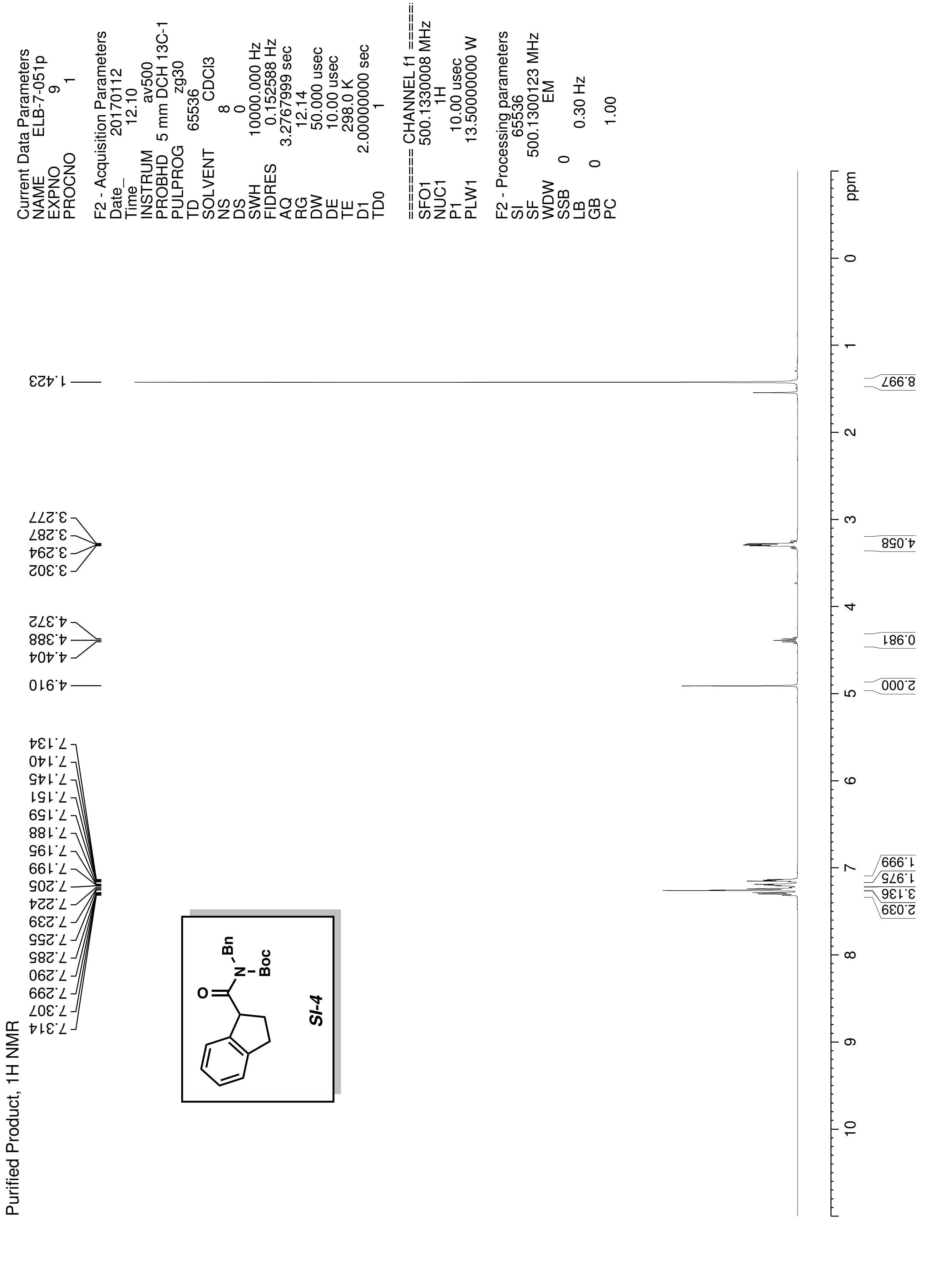
Purified Product, 1H NMR
7.314
7.307
7.299
7.290
7.285
7.255
7.239
7.224
7.205
7.199
7.195
7.188
7.159
7.151
7.145
7.140
7.134
4.910
4.404
4.388
4.372
3.302
3.294
3.287
3.277
1.423
O
N
Bn
Boc
SI-4
Current Data Parameters
NAME ELB-7-051p
EXPNO 9
PROCNO 1
F2 - Acquisition Parameters
Date_ 20170112
Time_ 12.10
INSTRUM av500
PROBHD 5 mm DCH 13C-1
PULPROG zg30
TD 65536
SOLVENT CDCl3
NS 8
DS 0
SWH 10000.000 Hz
FIDRES 0.152588 Hz
AQ 3.2767999 sec
RG 12.14
DW 50.000 usec
DE 10.00 usec
TE 298.0 K
D1 2.00000000 sec
TD0 1
======== CHANNEL f1 ========
SFO1 500.1330008 MHz
NUC1 1H
P1 10.00 usec
PLW1 13.50000000 W
F2 - Processing parameters
SI 65536
SF 500.1300123 MHz
WDW EM
SSB 0
LB 0.30 Hz
GB 0
PC 1.00
2.039
3.136
1.975
1.999
2.000
0.981
4.058
8.997
10
9
8
7
6
5
4
3
2
1
0
ppm

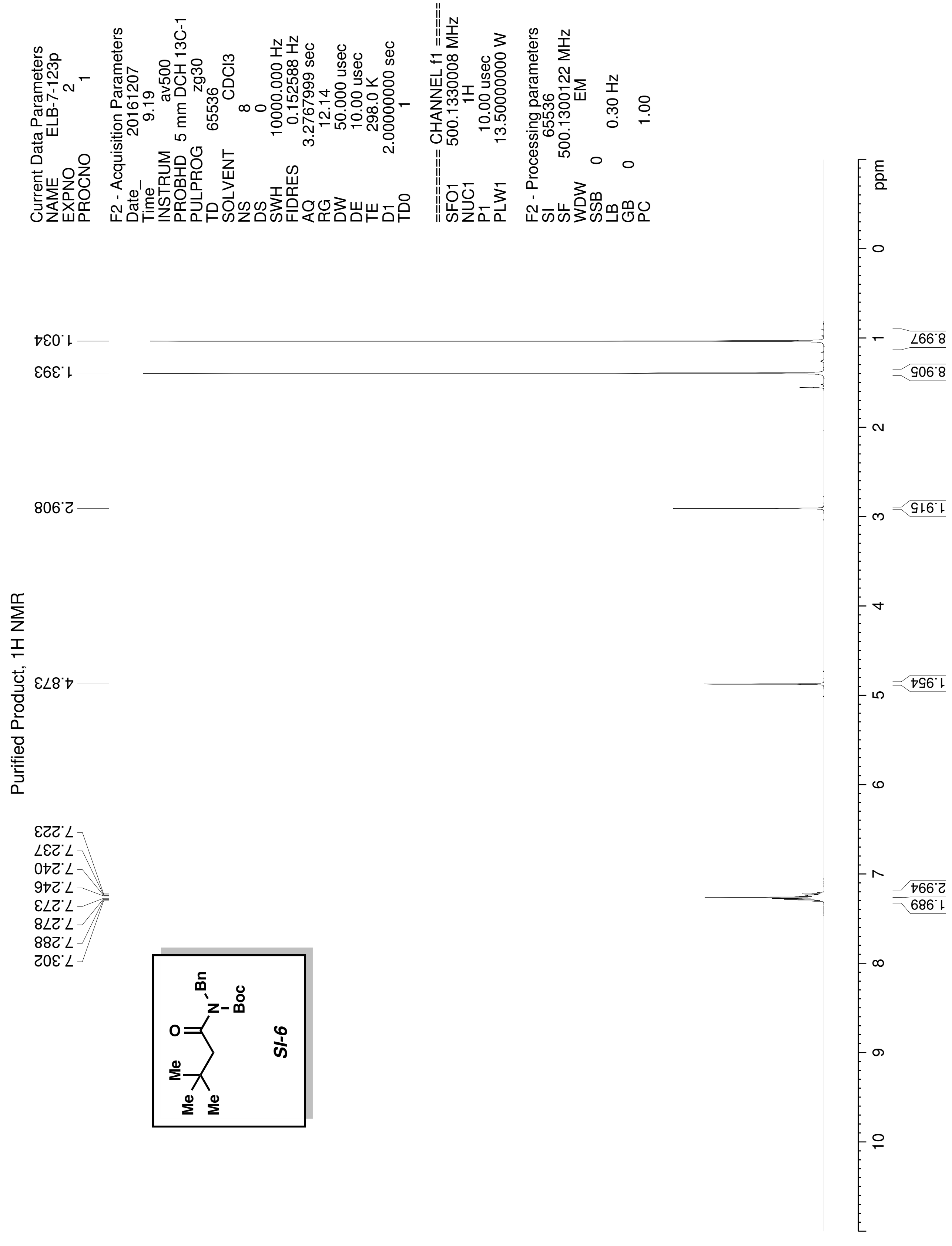
Purified Product, 1H NMR
7.302
7.288
7.278
7.273
7.246
7.240
7.237
7.223
4.873
2.908
1.393
1.034
Me
Me
Me
O
N
Bn
Boc
SI-6
Current Data Parameters
NAME ELB-7-123p
EXPNO 2
PROCNO 1
F2 - Acquisition Parameters
Date_ 20161207
Time 9.19
INSTRUM av500
PROBHD 5 mm DCH 13C-1
PULPROG zg30
TD 65536
SOLVENT CDCl3
NS 8
DS 0
SWH 10000.000 Hz
FIDRES 0.152588 Hz
AQ 3.2767999 sec
RG 12.14
DW 50.000 usec
DE 10.00 usec
TE 298.0 K
D1 2.00000000 sec
TD0 1
======== CHANNEL f1 ======
SFO1 500.1330008 MHz
NUC1 1H
P1 10.00 usec
PLW1 13.50000000 W
F2 - Processing parameters
SI 65536
SF 500.1300122 MHz
WDW EM
SSB 0
LB 0.30 Hz
GB 0
PC 1.00
8.997
8.905
1.915
1.954
2.994
1.989
ppm
0
1
2
3
4
5
6
7
8
9
10

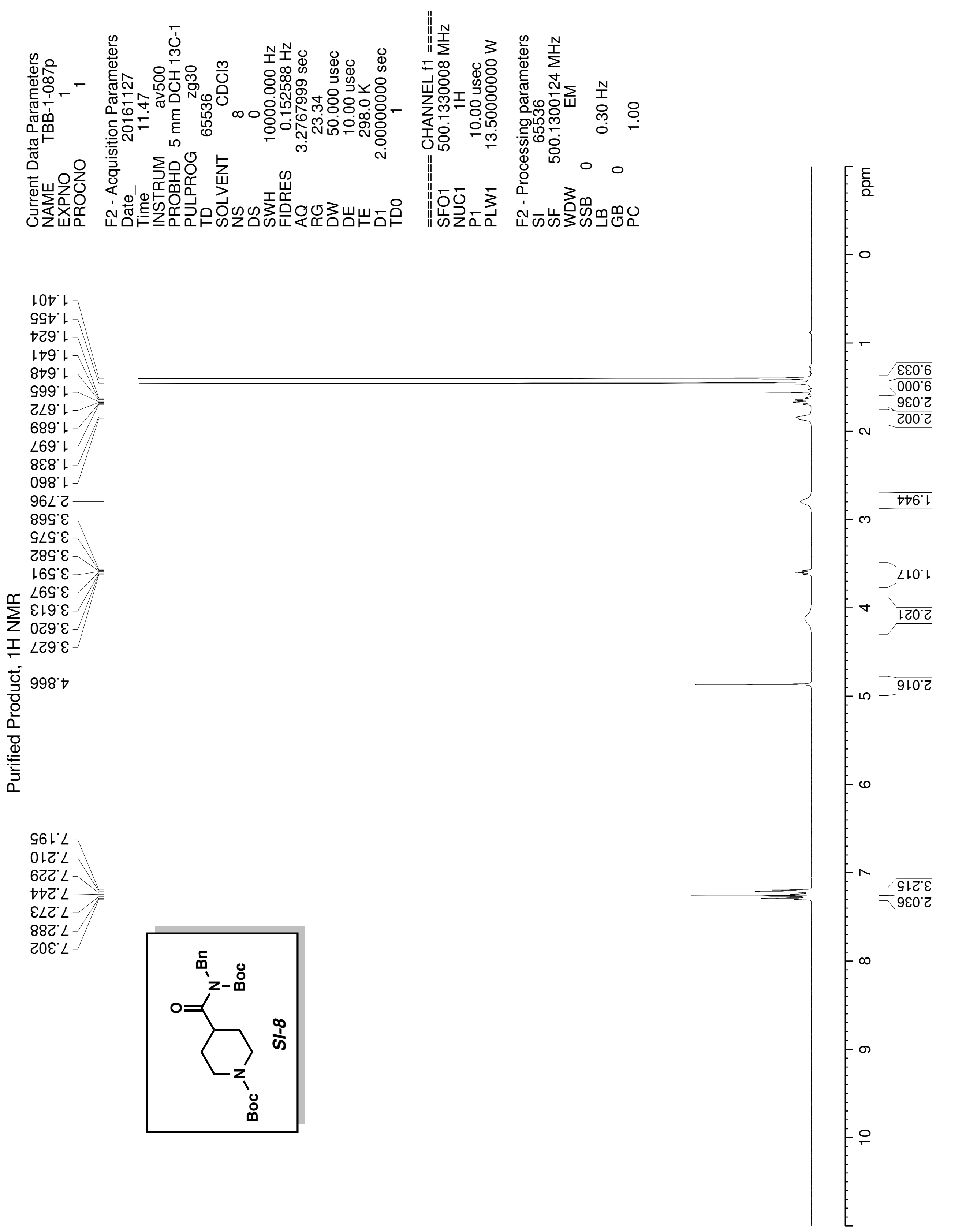
Purified Product, 1H NMR
SI-8
Boc
N
O
N
Bn
Boc
7.302
7.288
7.273
7.244
7.229
7.210
7.195
4.866
3.627
3.620
3.613
3.597
3.591
3.582
3.575
3.568
2.796
1.860
1.838
1.697
1.689
1.672
1.665
1.648
1.641
1.624
1.455
1.401
Current Data Parameters
NAME TBB-1-087p
EXPNO 1
PROCNO 1
F2 - Acquisition Parameters
Date_ 20161127
Time 11.47
INSTRUM av500
PROBHD 5 mm DCH 13C-1
PULPROG zg30
TD 65536
SOLVENT CDCl3
NS 8
DS 0
SWH 10000.000 Hz
FIDRES 0.152588 Hz
AQ 3.2767999 sec
RG 23.34
DW 50.000 usec
DE 10.00 usec
TE 298.0 K
D1 2.00000000 sec
TD0 1
======== CHANNEL f1 ========
SFO1 500.1330008 MHz
NUC1 1H
P1 10.00 usec
PLW1 13.50000000 W
F2 - Processing parameters
SI 65536
SF 500.1300124 MHz
WDW EM
SSB 0
LB 0.30 Hz
GB 0
PC 1.00
2.036
3.215
2.016
2.021
1.017
1.944
2.002
2.036
9.000
9.033
10
9
8
7
6
5
4
3
2
1
0
ppm

Purified Product, 1H NMR

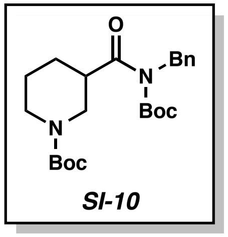

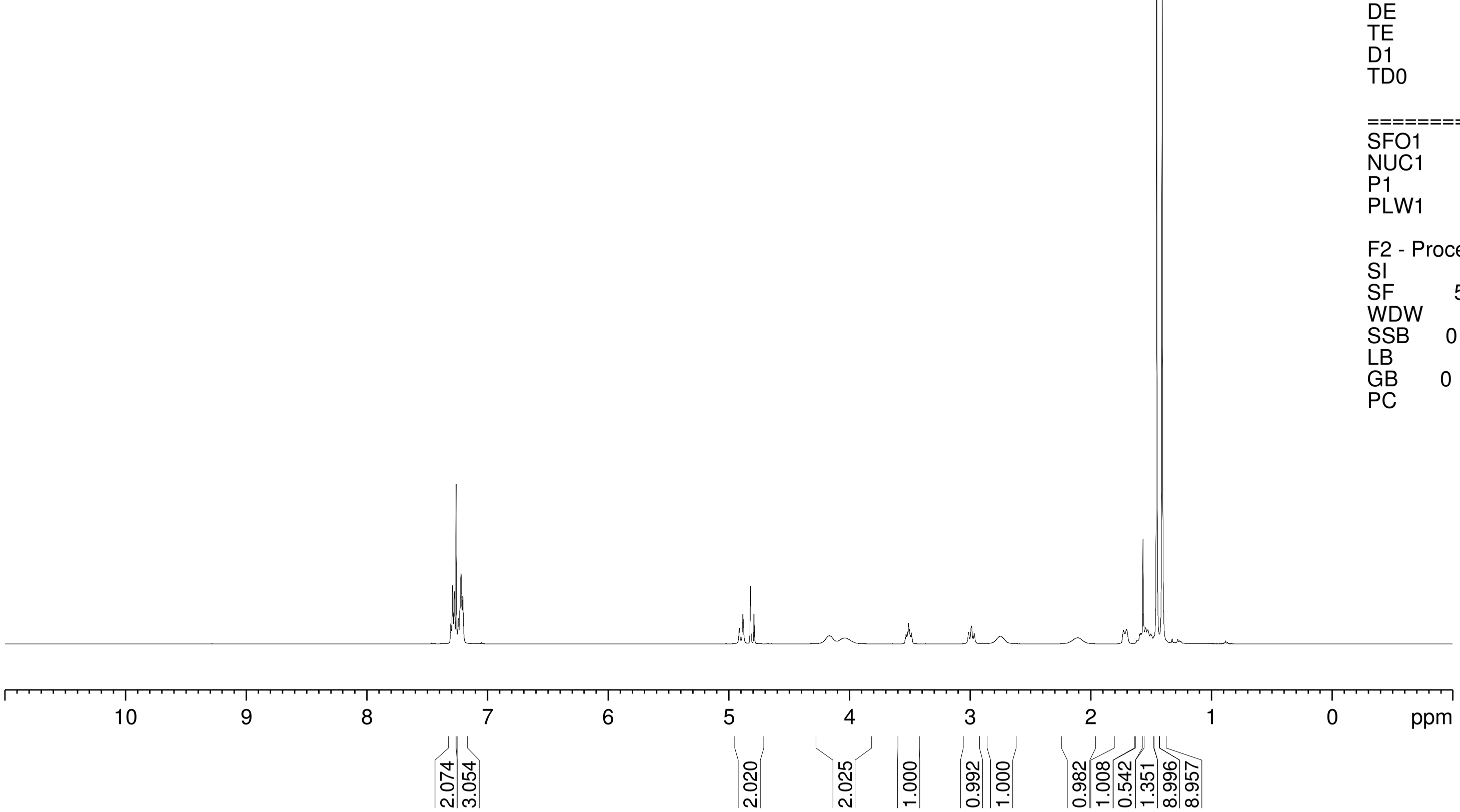

Current Data Parameters
NAME TBB-1-082p
EXPNO 1
PROCNO 1

F2 - Acquisition Parameters
Date_ 20161127
Time 12.13
INSTRUM av500
PROBHD 5 mm DCH 13C-1
PULPROG zg30
TD 65536
SOLVENT CDCl3
NS 8
DS 0
SWH 10000.000 Hz
FIDRES 0.152588 Hz
AQ 3.2767999 sec
RG 23.34
DW 50.000 usec
DE 10.00 usec
TE 298.0 K
D1 2.00000000 sec
TD0 1

======== CHANNEL f1 =====
SFO1 500.1330008 MHz
NUC1 1H
P1 10.00 usec
PLW1 13.50000000 W

F2 - Processing parameters
SI 65536
SF 500.1300123 MHz
WDW EM
SSB 0
LB 0.30 Hz
GB 0
PC 1.00

Purified Product, 1H NMR

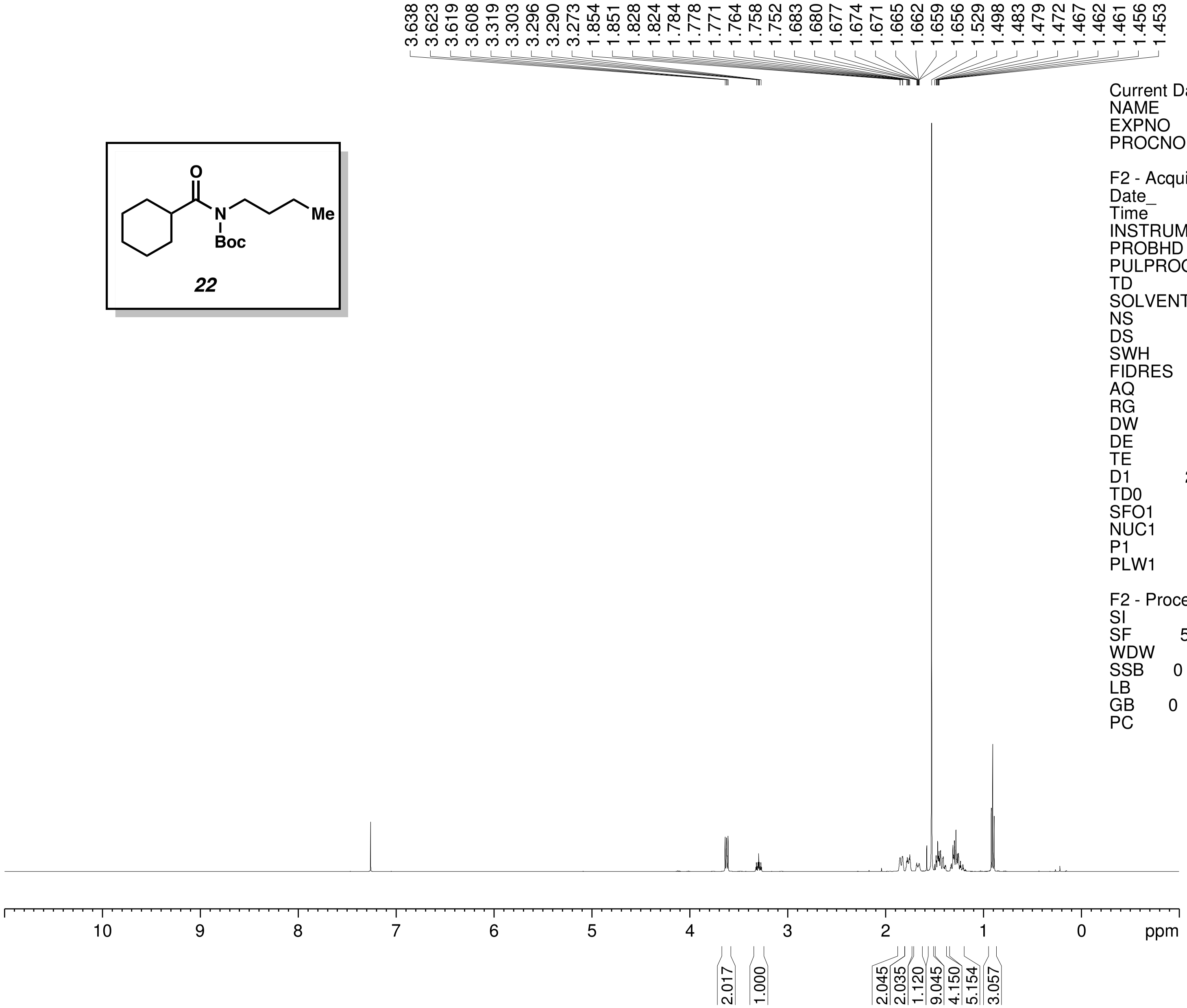

Current Data Parameters
NAME ELB-7-298p
EXPNO 9
PROCNO 1

F2 - Acquisition Parameters
Date_ 20170420
Time 13.10 h
INSTRUM av500
PROBHD Z119248_0002 (
PULPROG zg30
TD 65536
SOLVENT CDCl3
NS 8
DS 0
SWH 10000.000 Hz
FIDRES 0.305176 Hz
AQ 3.2767999 sec
RG 12.14
DW 50.000 usec
DE 10.00 usec
TE 298.0 K
D1 2.00000000 sec
TD0 1
SFO1 500.1330008 MHz
NUC1 1H
P1 10.00 usec
PLW1 13.50000000 W

F2 - Processing parameters
SI 65536
SF 500.1300122 MHz
WDW EM
SSB 0
LB 0.30 Hz
GB 0
PC 1.00

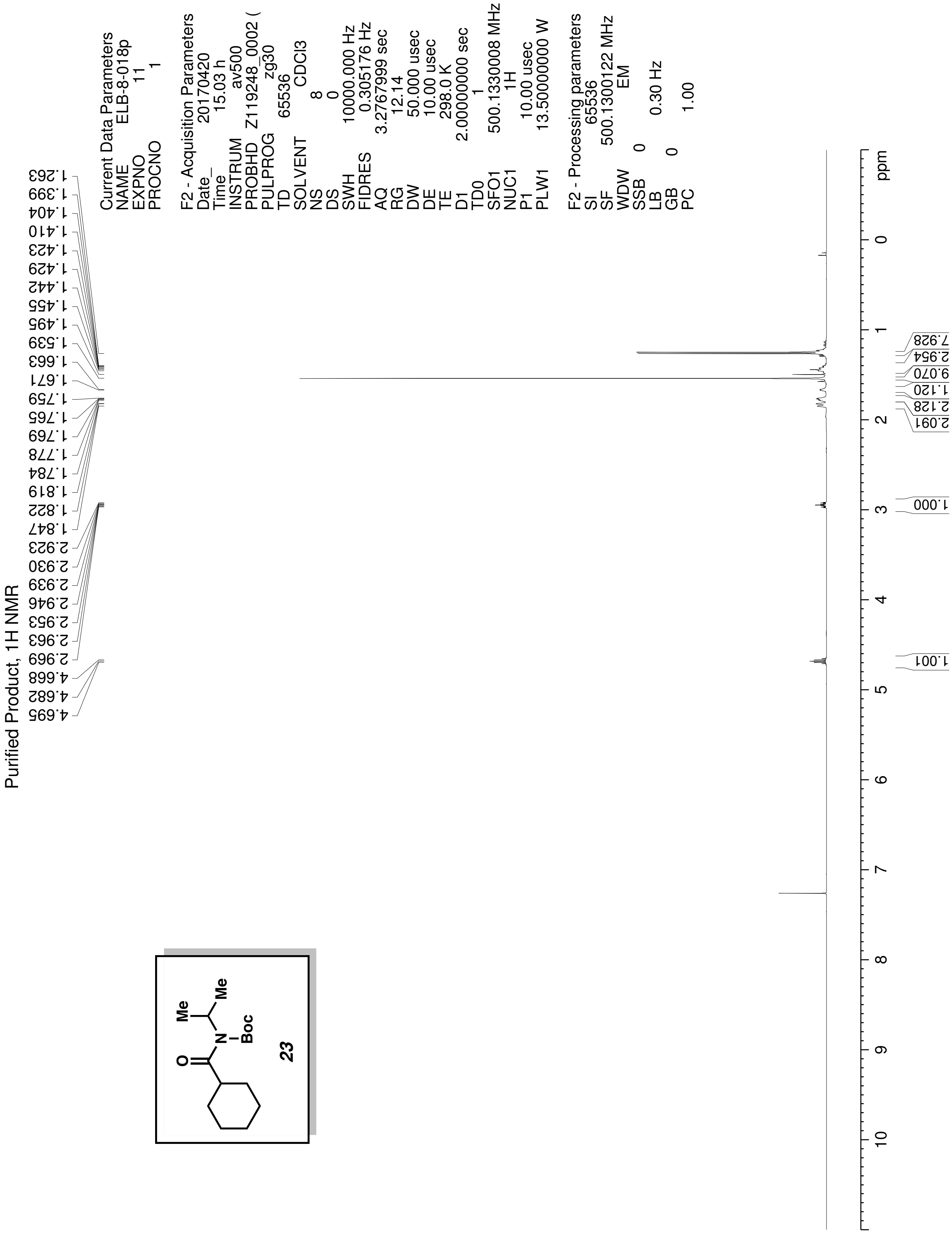
Purified Product, 1H NMR
1.263
1.399
1.404
1.410
1.423
1.429
1.442
1.455
1.495
1.539
1.663
1.671
1.759
1.765
1.769
1.778
1.784
1.819
1.822
1.847
2.923
2.930
2.939
2.946
2.953
2.963
2.969
4.668
4.682
4.695
Current Data Parameters
NAME ELB-8-018p
EXPNO 11
PROCNO 1
F2 - Acquisition Parameters
Date_ 20170420
Time 15.03 h
INSTRUM av500
PROBHD Z119248_0002 (
PULPROG zg30
TD 65536
SOLVENT CDCl3
NS 8
DS 0
SWH 10000.000 Hz
FIDRES 0.305176 Hz
AQ 3.2767999 sec
RG 12.14
DW 50.000 usec
DE 10.00 usec
TE 298.0 K
D1 2.00000000 sec
TD0 1
SFO1 500.1330008 MHz
NUC1 1H
P1 10.00 usec
PLW1 13.50000000 W
F2 - Processing parameters
SI 65536
SF 500.1300122 MHz
WDW EM
SSB 0
LB 0.30 Hz
GB 0
PC 1.00
Me
Me
O
N
Boc
23
7.928
2.954
9.070
1.120
2.128
2.091
1.000
1.001
ppm
0
1
2
3
4
5
6
7
8
9
10

Purified Product, 1H NMR

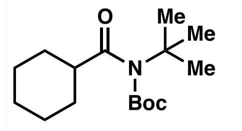

24

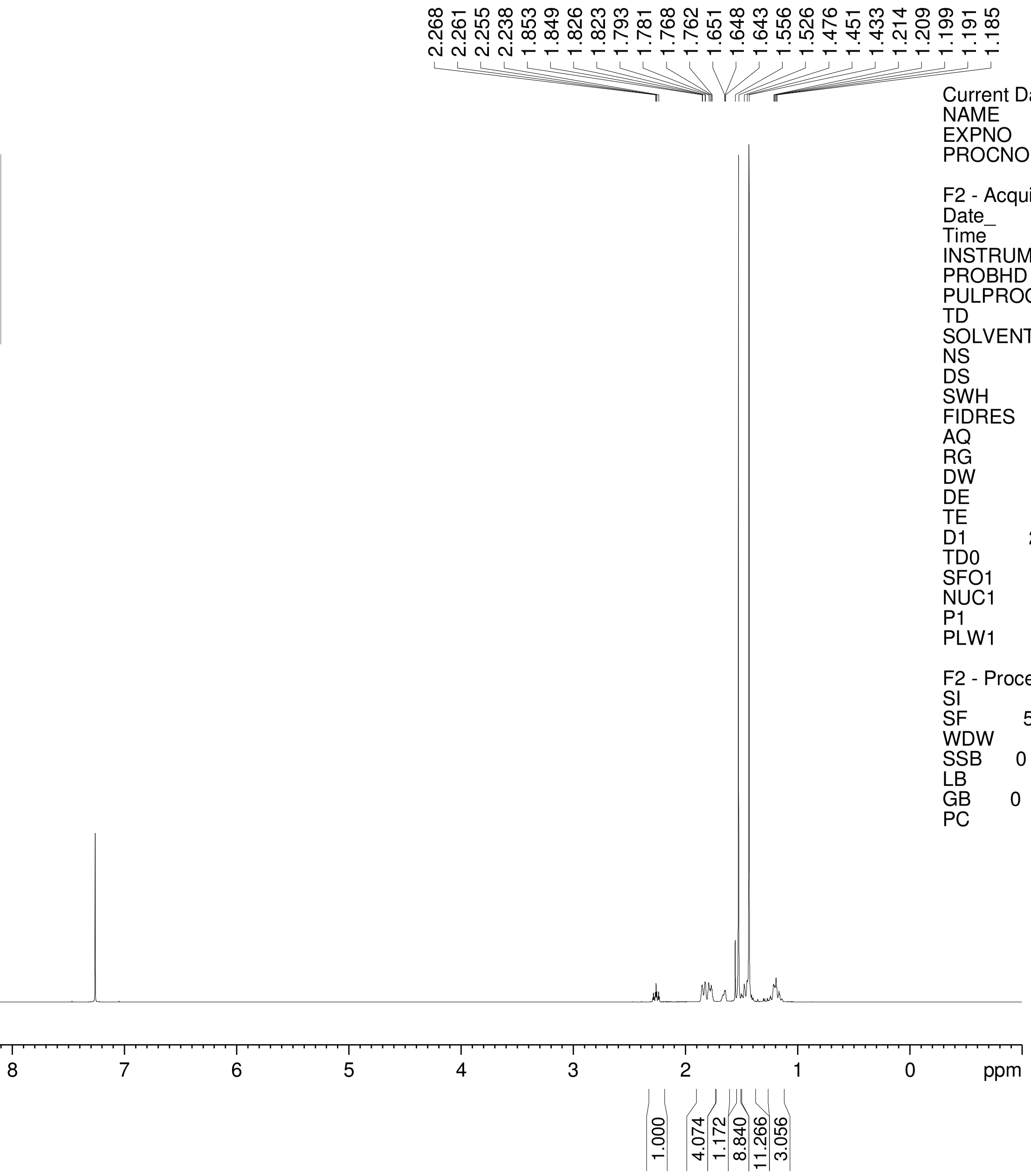

Current Data Parameters
NAME JED-3-152p
EXPNO 1
PROCNO 1

F2 - Acquisition Parameters
Date_ 20170501
Time 12.53 h
INSTRUM av500
PROBHD Z119248_0002 (
PULPROG zg30
TD 65536
SOLVENT CDCl3
NS 8
DS 0
SWH 10000.000 Hz
FIDRES 0.305176 Hz
AQ 3.2767999 sec
RG 12.14
DW 50.000 usec
DE 10.00 usec
TE 298.0 K
D1 2.00000000 sec
TD0 1
SFO1 500.1330008 MHz
NUC1 1H
P1 10.00 usec
PLW1 13.50000000 W

F2 - Processing parameters
SI 65536
SF 500.1300122 MHz
WDW EM
SSB 0
LB 0.30 Hz
GB 0
PC 1.00

Purified Product, 1H NMR

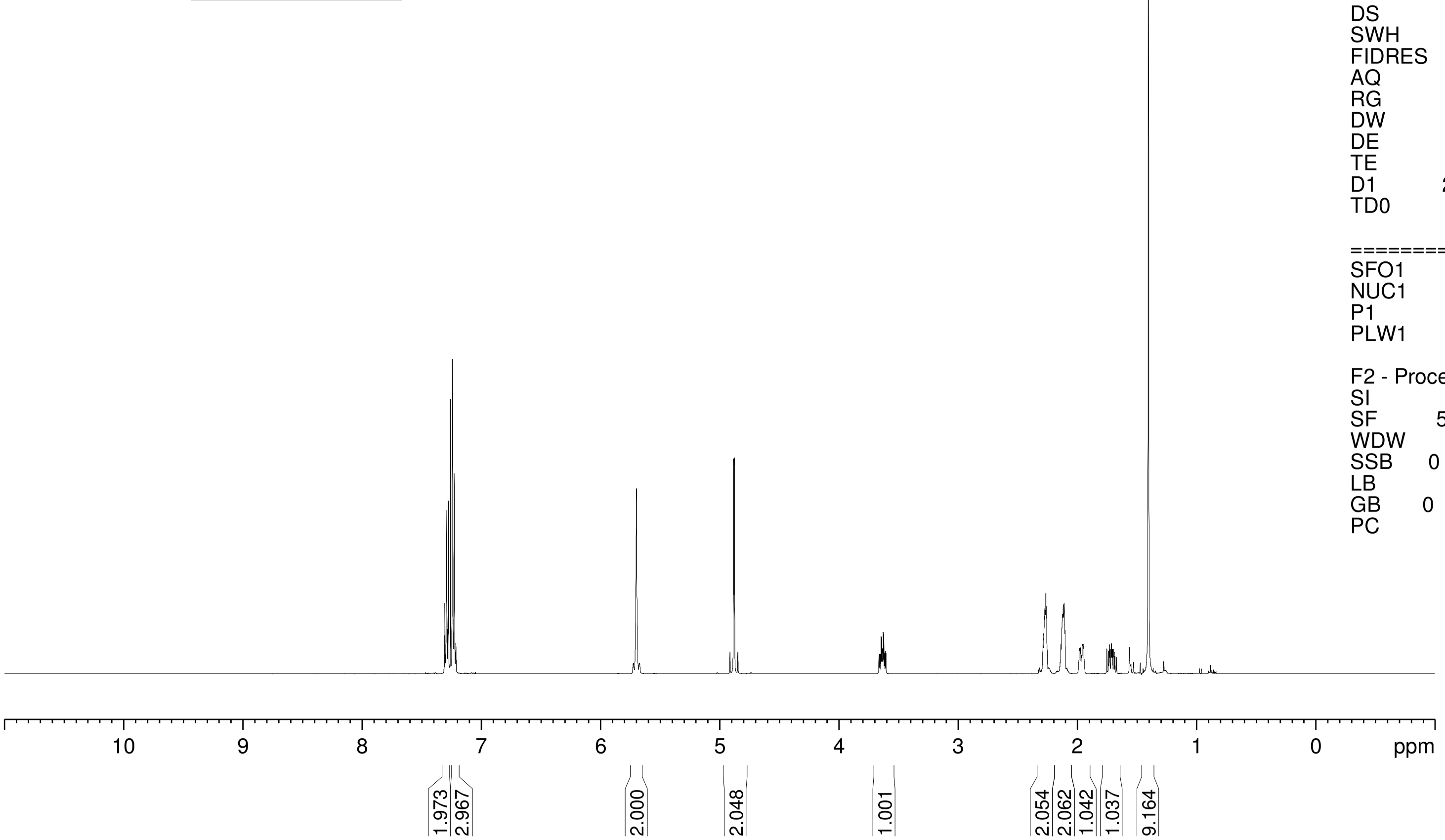

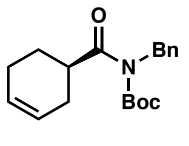

38

Current Data Parameters	
NAME	TBB-1-168p
EXPNO	1
PROCNO	1
F2 - Acquisition Parameters	
Date_	20161128
Time	13.22
INSTRUM	av500
PROBHD	5 mm DCH 13C-1
PULPROG	zg30
TD	65536
SOLVENT	CDCl3
NS	8
DS	0
SWH	10000.000 Hz
FIDRES	0.152588 Hz
AQ	3.2767999 sec
RG	12.14
DW	50.000 usec
DE	10.00 usec
TE	298.0 K
D1	2.00000000 sec
TD0	1
======== CHANNEL f1 =====	
SFO1	500.1330008 MHz
NUC1	1H
P1	10.00 usec
PLW1	13.50000000 W
F2 - Processing parameters	
SI	65536
SF	500.1300125 MHz
WDW	EM
SSB	0
LB	0.30 Hz
GB	0
PC	1.00

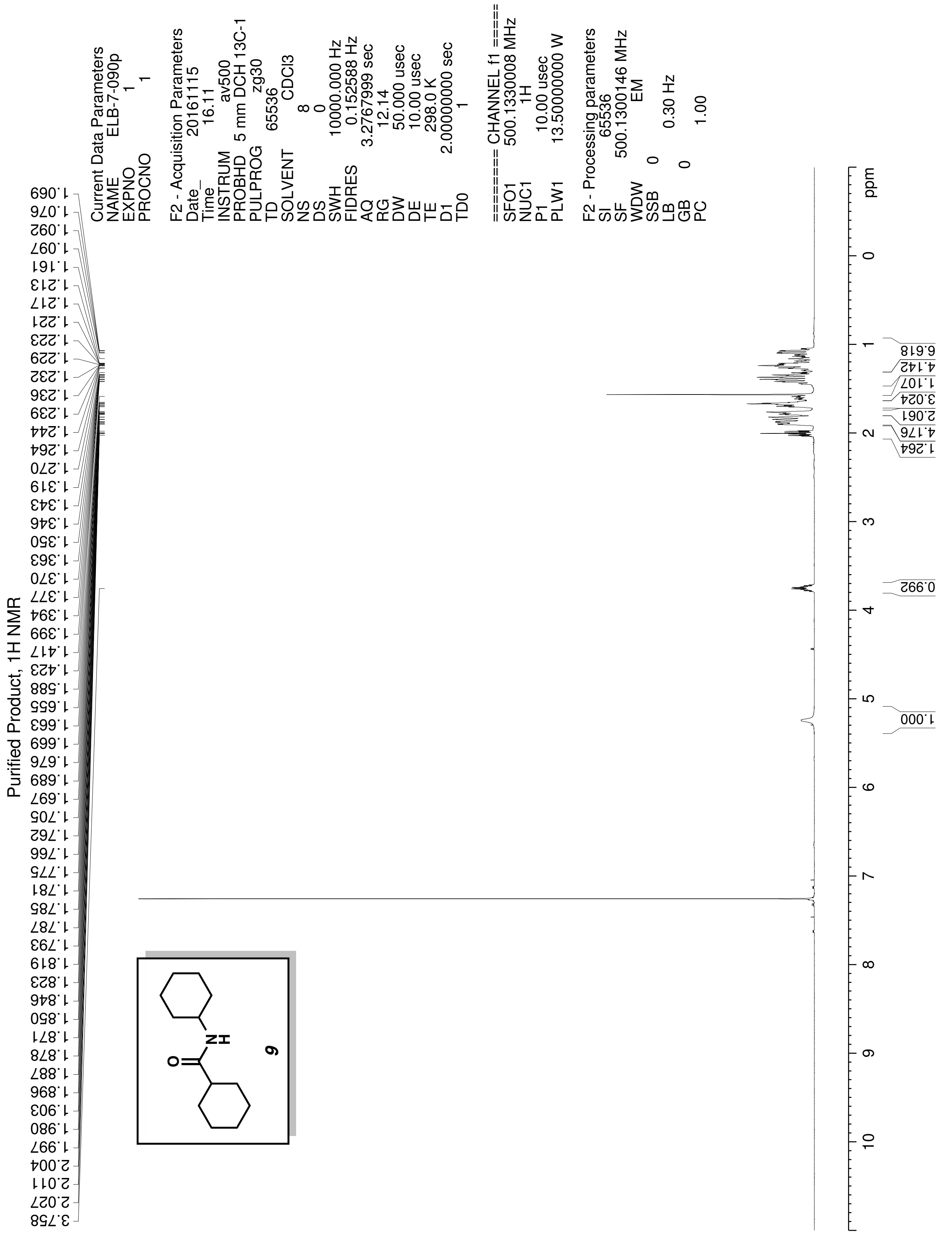
Purified Product, 1H NMR
1.069
1.076
1.092
1.097
1.161
1.213
1.217
1.221
1.223
1.229
1.232
1.236
1.239
1.244
1.264
1.270
1.319
1.343
1.346
1.350
1.363
1.370
1.377
1.394
1.399
1.417
1.423
1.588
1.655
1.663
1.669
1.676
1.689
1.697
1.705
1.762
1.766
1.775
1.781
1.785
1.787
1.793
1.819
1.823
1.846
1.850
1.871
1.878
1.887
1.896
1.903
1.980
1.997
2.004
2.011
2.027
3.758
Current Data Parameters
NAME ELB-7-090p
EXPNO 1
PROCNO 1
F2 - Acquisition Parameters
Date_ 20161115
Time_ 16.11
INSTRUM av500
PROBHD 5 mm DCH 13C-1
PULPROG zg30
TD 65536
SOLVENT CDCl3
NS 8
DS 0
SWH 10000.000 Hz
FIDRES 0.152588 Hz
AQ 3.2767999 sec
RG 12.14
DW 50.000 usec
DE 10.00 usec
TE 298.0 K
D1 2.00000000 sec
TD0 1
======== CHANNEL f1 ========
SFO1 500.1330008 MHz
NUC1 1H
P1 10.00 usec
PLW1 13.50000000 W
F2 - Processing parameters
SI 65536
SF 500.1300146 MHz
WDW EM
SSB 0
LB 0.30 Hz
GB 0
PC 1.00
N
H
O
9
6.618
4.142
1.107
3.024
2.061
4.176
1.264
0.992
1.000
ppm
0
1
2
3
4
5
6
7
8
9
10

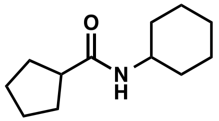

15

Purified Product, 1H NMR

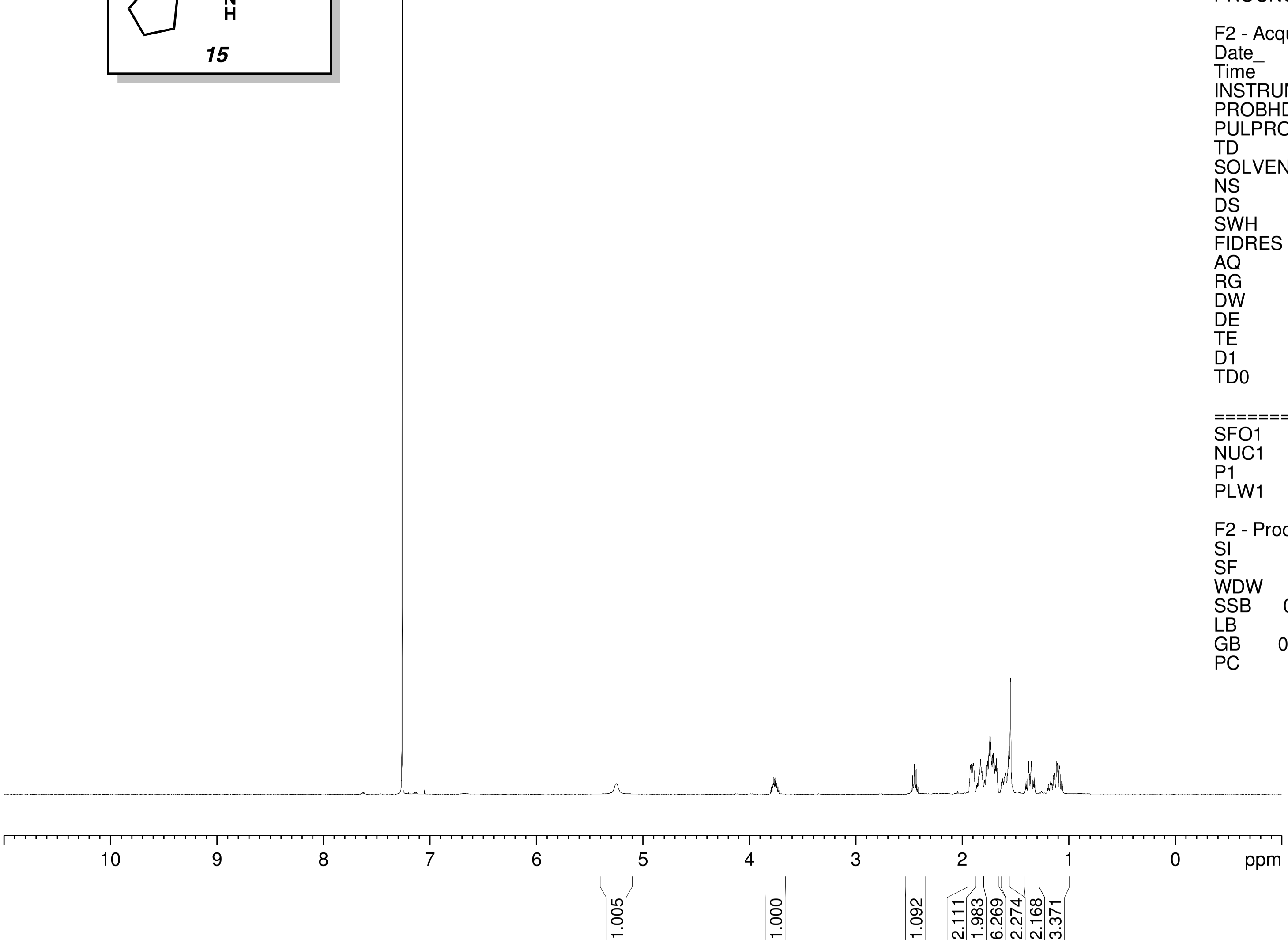

Current Data Parameters
NAME ELB-7-079p
EXPNO 1
PROCNO 1

F2 - Acquisition Parameters
Date_ 20161111
Time 10.17
INSTRUM av500
PROBHD 5 mm DCH 13C-1
PULPROG zg30
TD 65536
SOLVENT CDCl3
NS 8
DS 0
SWH 10000.000 Hz
FIDRES 0.152588 Hz
AQ 3.2767999 sec
RG 12.14
DW 50.000 usec
DE 10.00 usec
TE 298.0 K
D1 2.00000000 sec
TD0 1

======== CHANNEL f1 =====
SFO1 500.1330008 MHz
NUC1 1H
P1 10.00 usec
PLW1 13.50000000 W

F2 - Processing parameters
SI 65536
SF 500.1300122 MHz
WDW EM
SSB 0
LB 0.30 Hz
GB 0
PC 1.00

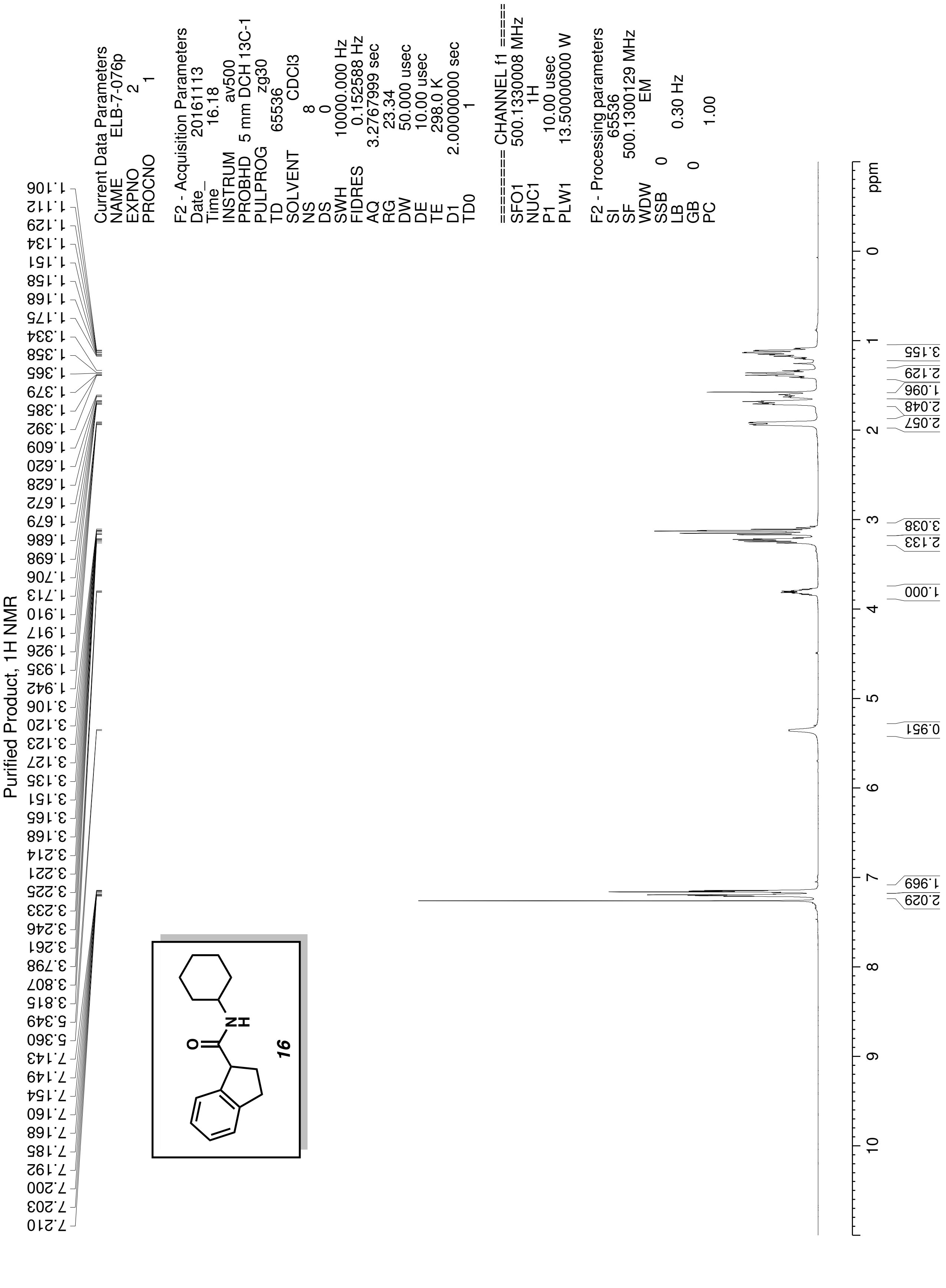
Purified Product, 1H NMR
1.106 1.112 1.129 1.134 1.151 1.158 1.168 1.175 1.334 1.358 1.365 1.379 1.385 1.392 1.609 1.620 1.628 1.672 1.679 1.686 1.698 1.706 1.713 1.910 1.917 1.926 1.935 1.942 3.106 3.120 3.123 3.127 3.135 3.151 3.165 3.168 3.214 3.221 3.225 3.233 3.246 3.261 3.798 3.807 3.815 5.349 5.360 7.143 7.149 7.154 7.160 7.168 7.185 7.192 7.200 7.203 7.210
Current Data Parameters
NAME ELB-7-076p
EXPNO 2
PROCNO 1
F2 - Acquisition Parameters
Date_ 20161113
Time 16.18
INSTRUM av500
PROBHD 5 mm DCH 13C-1
PULPROG zg30
TD 65536
SOLVENT CDCl3
NS 8
DS 0
SWH 10000.000 Hz
FIDRES 0.152588 Hz
AQ 3.2767999 sec
RG 23.34
DW 50.000 usec
DE 10.00 usec
TE 298.0 K
D1 2.00000000 sec
TD0 1
======== CHANNEL f1 ======
SFO1 500.1330008 MHz
NUC1 1H
P1 10.00 usec
PLW1 13.50000000 W
F2 - Processing parameters
SI 65536
SF 500.1300129 MHz
WDW EM
SSB 0
LB 0.30 Hz
GB 0
PC 1.00
O
N
H
16
3.155 2.129 1.096 2.048 2.057 3.038 2.133 1.000 0.951 1.969 2.029
0 1 2 3 4 5 6 7 8 9 10
ppm

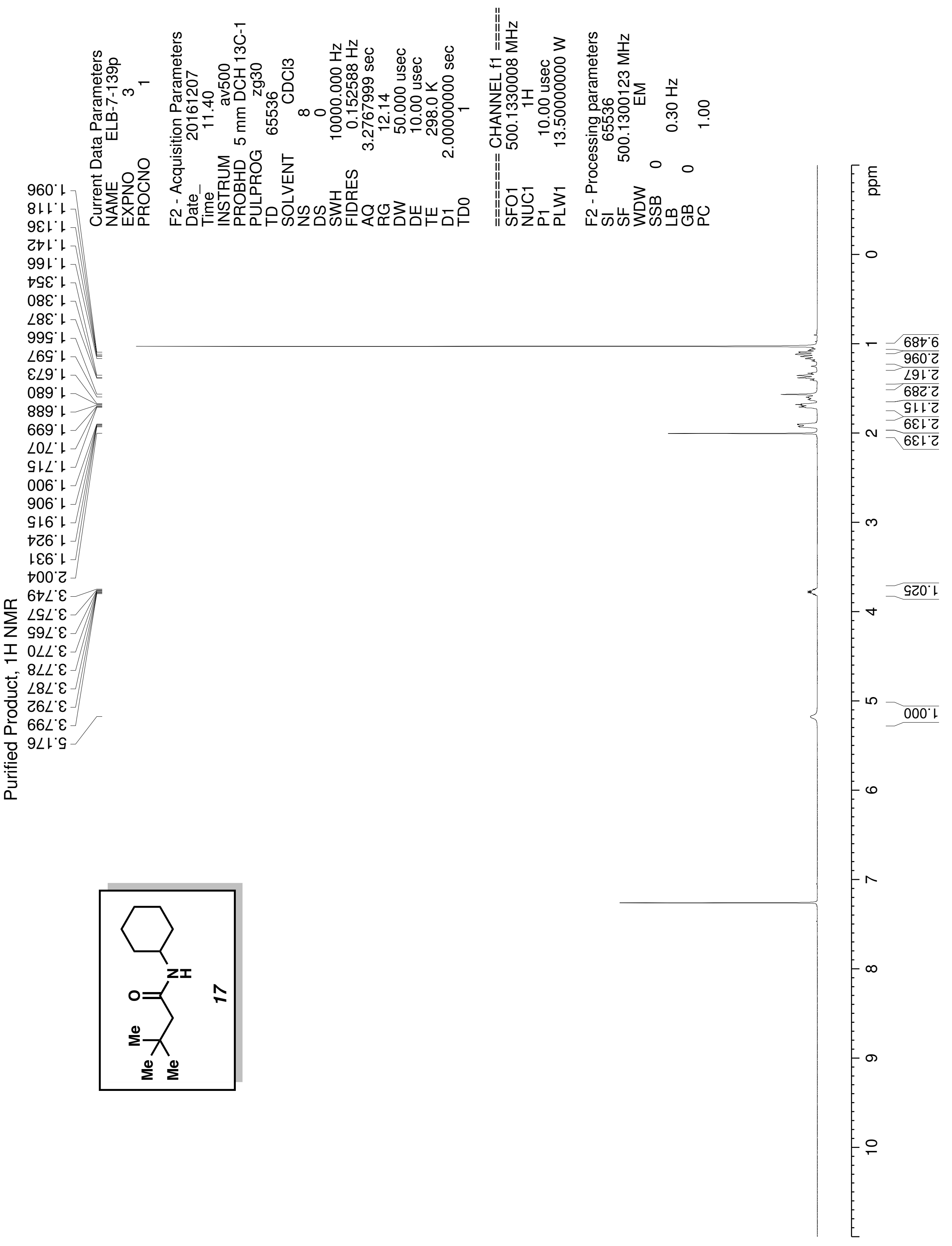
Purified Product, 1H NMR
5.176
3.799
3.792
3.787
3.778
3.770
3.765
3.757
3.749
2.004
1.931
1.924
1.915
1.906
1.900
1.715
1.707
1.699
1.688
1.680
1.673
1.597
1.566
1.387
1.380
1.354
1.166
1.142
1.136
1.118
1.096
Current Data Parameters
NAME ELB-7-139p
EXPNO 3
PROCNO 1
F2 - Acquisition Parameters
Date_ 20161207
Time 11.40
INSTRUM av500
PROBHD 5 mm DCH 13C-1
PULPROG zg30
TD 65536
SOLVENT CDCl3
NS 8
DS 0
SWH 10000.000 Hz
FIDRES 0.152588 Hz
AQ 3.2767999 sec
RG 12.14
DW 50.000 usec
DE 10.00 usec
TE 298.0 K
D1 2.00000000 sec
TD0 1
======== CHANNEL f1 ========
SFO1 500.1330008 MHz
NUC1 1H
P1 10.00 usec
PLW1 13.50000000 W
F2 - Processing parameters
SI 65536
SF 500.1300123 MHz
WDW EM
SSB 0
LB 0.30 Hz
GB 0
PC 1.00
Me
Me
Me
O
N
H
17
9.489
2.096
2.167
2.289
2.115
2.139
2.139
1.025
1.000
10
9
8
7
6
5
4
3
2
1
0
ppm

Purified Product, 1H NMR

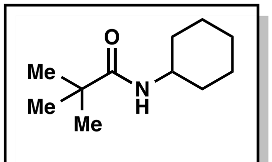

18

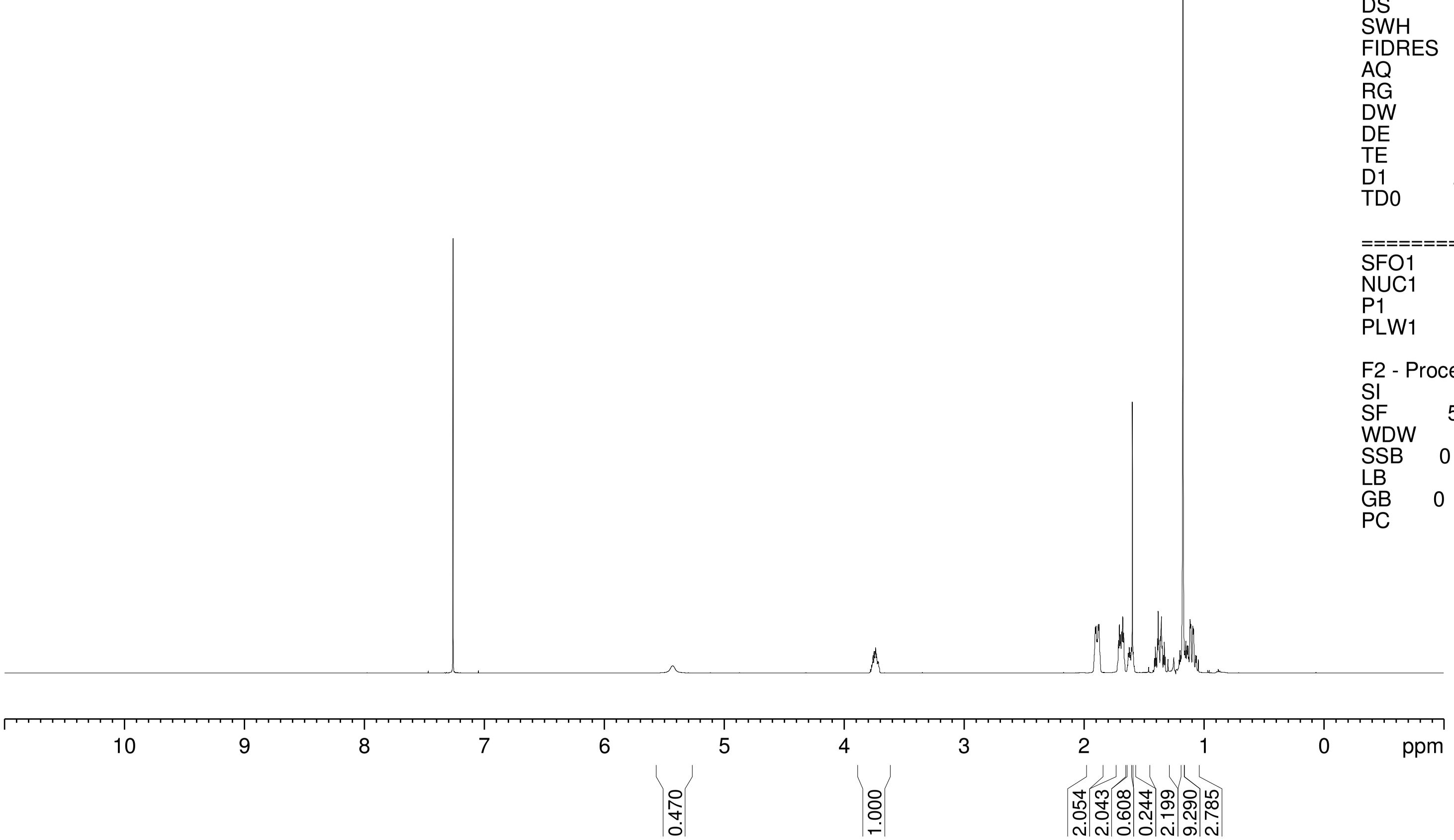

Current Data Parameters
NAME ELB-7-070p
EXPNO 1
PROCNO 1

F2 - Acquisition Parameters
Date_ 20161109
Time 16.18
INSTRUM av500
PROBHD 5 mm DCH 13C-1
PULPROG zg30
TD 65536
SOLVENT CDCl3
NS 8
DS 0
SWH 10000.000 Hz
FIDRES 0.152588 Hz
AQ 3.2767999 sec
RG 12.14
DW 50.000 usec
DE 10.00 usec
TE 298.0 K
D1 2.00000000 sec
TD0 1

======== CHANNEL f1 =====
SFO1 500.1330008 MHz
NUC1 1H
P1 10.00 usec
PLW1 13.50000000 W

F2 - Processing parameters
SI 65536
SF 500.1300122 MHz
WDW EM
SSB 0
LB 0.30 Hz
GB 0
PC 1.00

Purified Product, 1H NMR

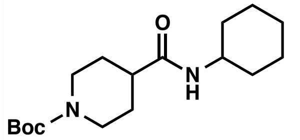

19

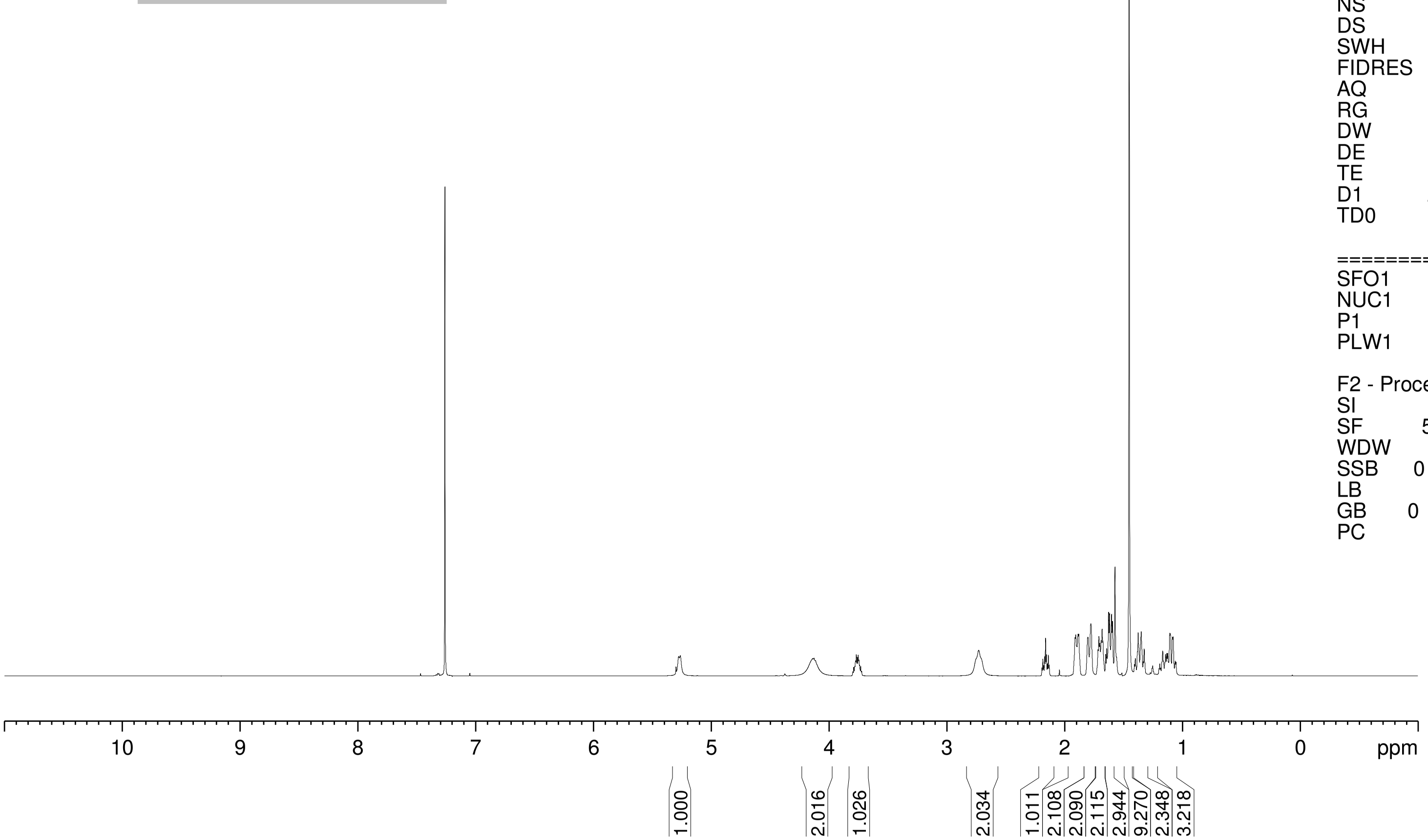

Current Data Parameters
NAME ELB-7-091p
EXPNO 1
PROCNO 1

F2 - Acquisition Parameters
Date_ 20161127
Time 12.01
INSTRUM av500
PROBHD 5 mm DCH 13C-1
PULPROG zg30
TD 65536
SOLVENT CDCl3
NS 8
DS 0
SWH 10000.000 Hz
FIDRES 0.152588 Hz
AQ 3.2767999 sec
RG 26.59
DW 50.000 usec
DE 10.00 usec
TE 298.0 K
D1 2.00000000 sec
TD0 1

======== CHANNEL f1 =====
SFO1 500.1330008 MHz
NUC1 1H
P1 10.00 usec
PLW1 13.50000000 W

F2 - Processing parameters
SI 65536
SF 500.1300122 MHz
WDW EM
SSB 0
LB 0.30 Hz
GB 0
PC 1.00

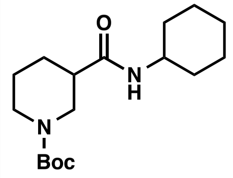

20

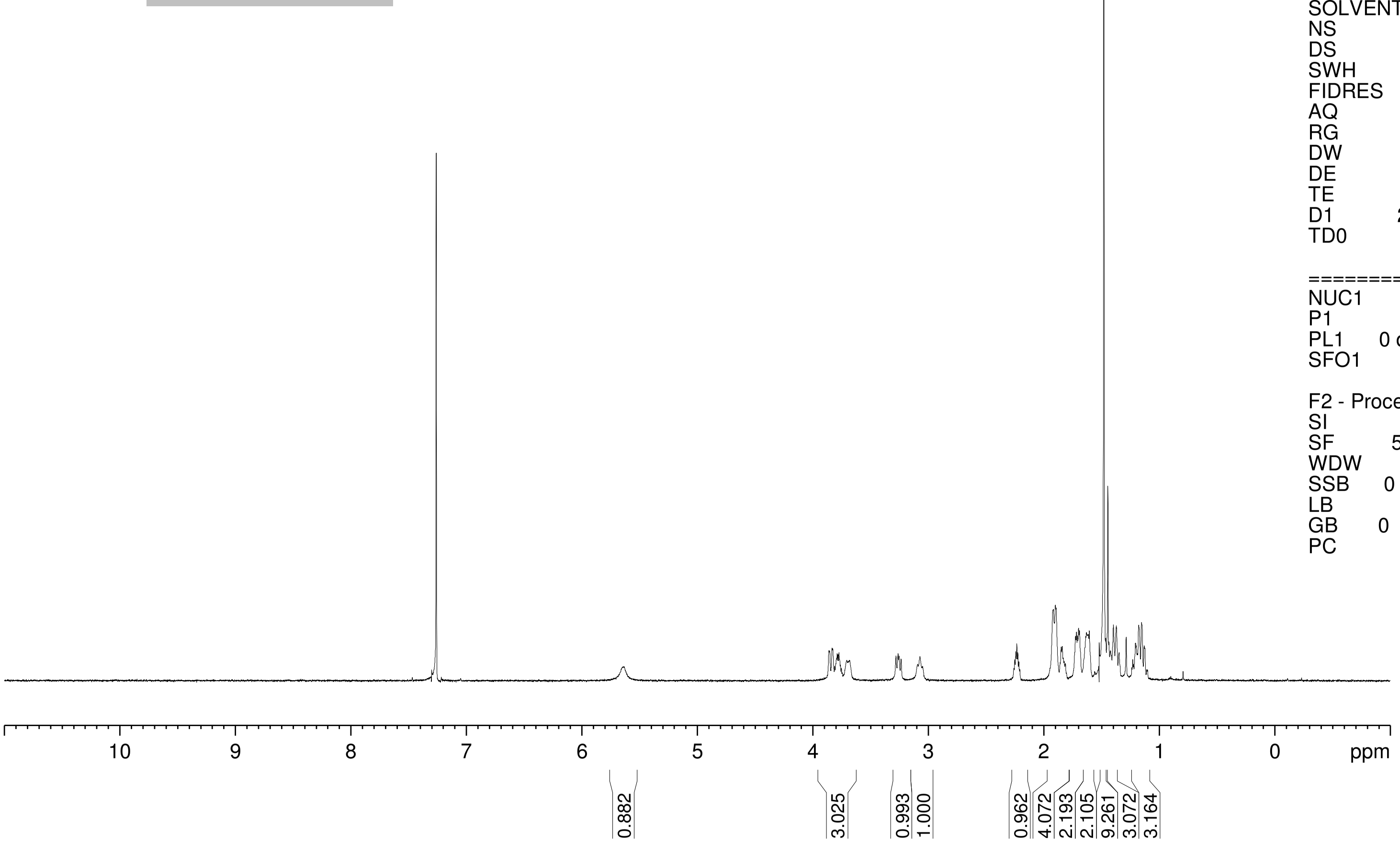

Current Data Parameters
NAME ELB-7-043p
EXPNO 14
PROCNO 1

F2 - Acquisition Parameters
Date_ 20170317
Time 13.50
INSTRUM drx500
PROBHD 5 mm bb-Z Z800
PULPROG zg30
TD 65536
SOLVENT CDCl3
NS 8
DS 0
SWH 10000.000 Hz
FIDRES 0.152588 Hz
AQ 3.2767999 sec
RG 203.2
DW 50.000 usec
DE 6.00 usec
TE 333.0 K
D1 2.00000000 sec
TD0 1

======== CHANNEL f1 =====
NUC1 1H
P1 13.30 usec
PL1 0 dB
SFO1 500.3330020 MHz

F2 - Processing parameters
SI 32768
SF 500.3300205 MHz
WDW EM
SSB 0
LB 0.30 Hz
GB 0
PC 1.00

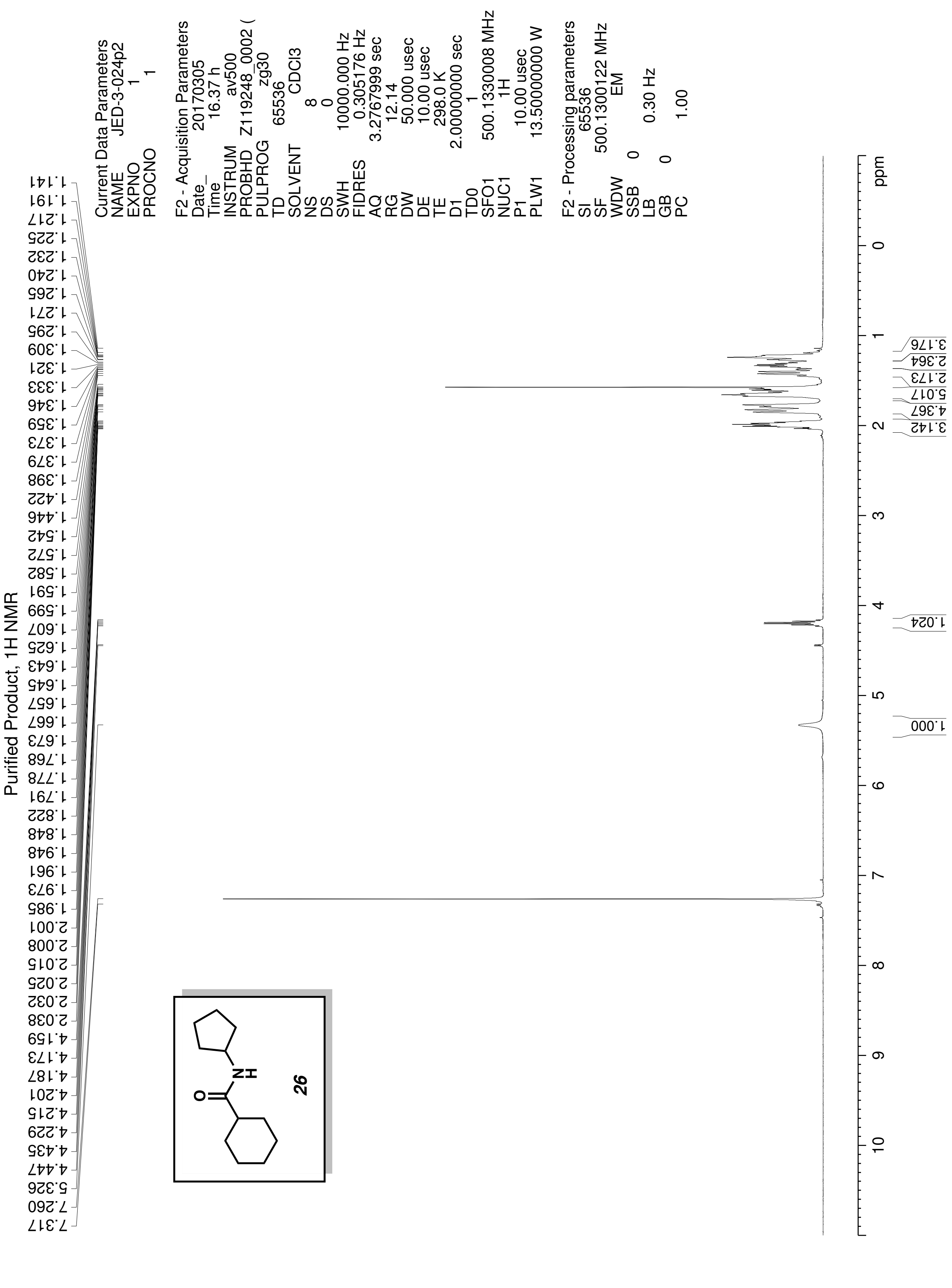
Purified Product, 1H NMR
1.141
1.191
1.217
1.225
1.232
1.240
1.265
1.271
1.295
1.309
1.321
1.333
1.346
1.359
1.373
1.379
1.398
1.422
1.446
1.542
1.572
1.582
1.591
1.599
1.607
1.625
1.643
1.645
1.657
1.667
1.673
1.768
1.778
1.791
1.822
1.848
1.948
1.961
1.973
1.985
2.001
2.008
2.015
2.025
2.032
2.038
4.159
4.173
4.187
4.201
4.215
4.229
4.435
4.447
5.326
7.260
7.317
Current Data Parameters
NAME JED-3-024p2
EXPNO 1
PROCNO 1
F2 - Acquisition Parameters
Date_ 20170305
Time_ 16.37 h
INSTRUM av500
PROBHD Z119248_0002 (
PULPROG zg30
TD 65536
SOLVENT CDCl3
NS 8
DS 0
SWH 10000.000 Hz
FIDRES 0.305176 Hz
AQ 3.2767999 sec
RG 12.14
DW 50.000 usec
DE 10.00 usec
TE 298.0 K
D1 2.00000000 sec
TD0 1
SFO1 500.1330008 MHz
NUC1 1H
P1 10.00 usec
PLW1 13.50000000 W
F2 - Processing parameters
SI 65536
SF 500.1300122 MHz
WDW EM
SSB 0
LB 0.30 Hz
GB 0
PC 1.00
O
N
H
26
3.176
2.364
2.173
5.017
4.367
3.142
1.024
1.000
ppm
0
1
2
3
4
5
6
7
8
9
10

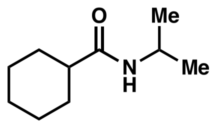

27

Purified Product, 1H NMR

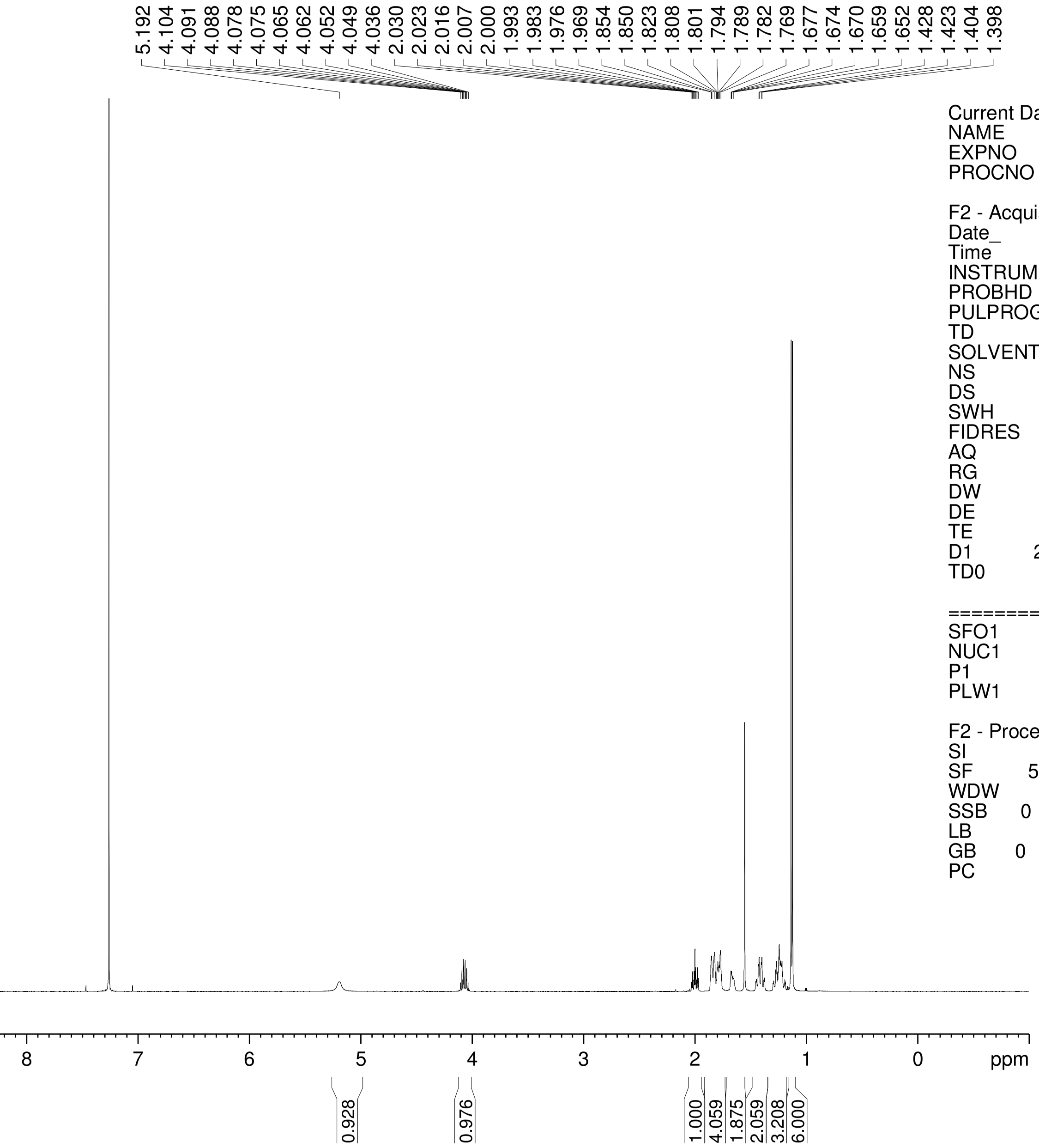

Current Data Parameters
NAME ELB-7-166p
EXPNO 1
PROCNO 1

F2 - Acquisition Parameters
Date_ 20170105
Time 11.17
INSTRUM av500
PROBHD 5 mm DCH 13C-1
PULPROG zg30
TD 65536
SOLVENT CDCl3
NS 8
DS 0
SWH 10000.000 Hz
FIDRES 0.152588 Hz
AQ 3.2767999 sec
RG 12.14
DW 50.000 usec
DE 10.00 usec
TE 298.0 K
D1 2.00000000 sec
TD0 1

======== CHANNEL f1 =====
SFO1 500.1330008 MHz
NUC1 1H
P1 10.00 usec
PLW1 13.50000000 W

F2 - Processing parameters
SI 65536
SF 500.1300123 MHz
WDW EM
SSB 0
LB 0.30 Hz
GB 0
PC 1.00

Purified Product, 1H NMR

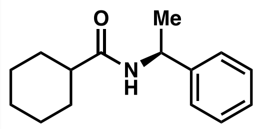

28

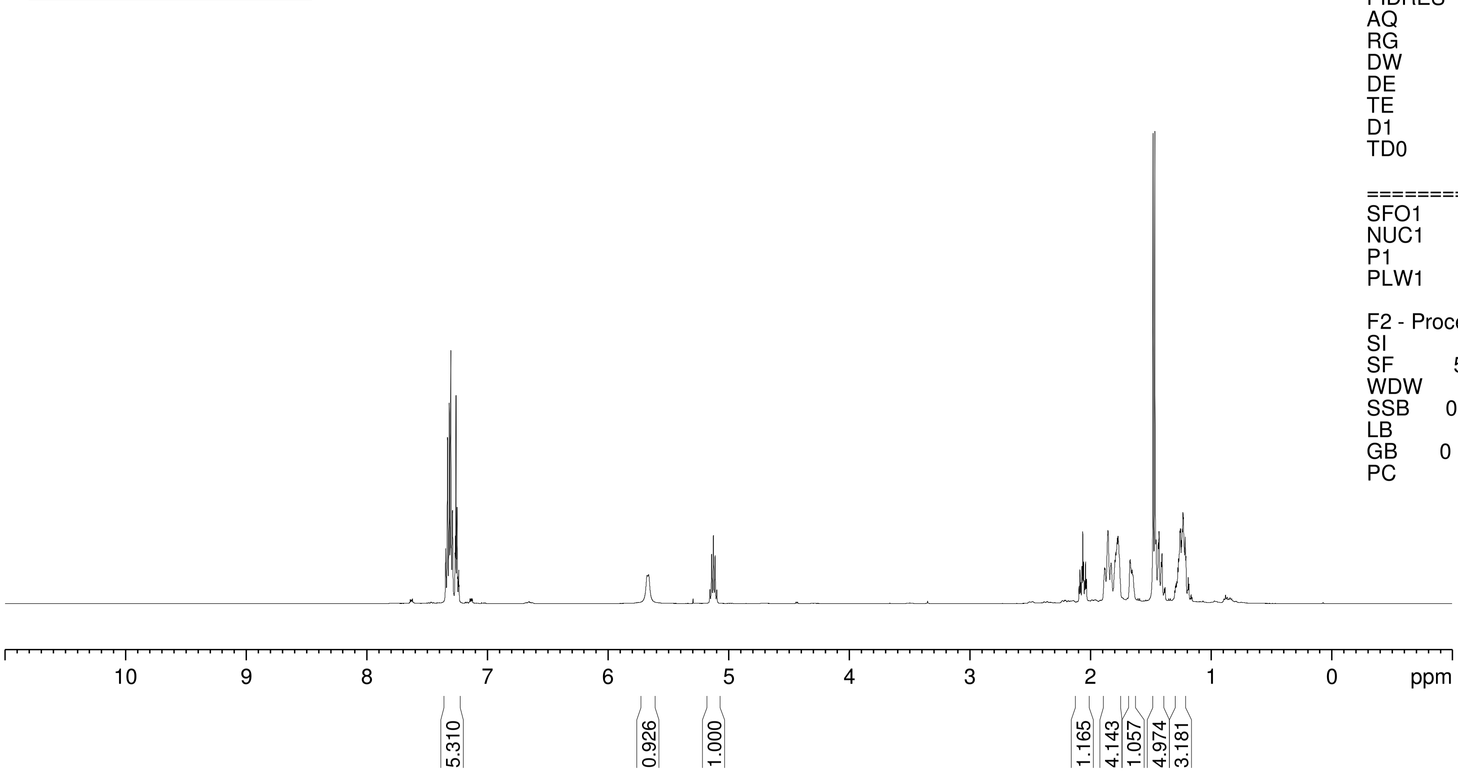

Current Data Parameters
NAME JED-3-064p
EXPNO 1
PROCNO 1

F2 - Acquisition Parameters
Date_ 20170116
Time 16.20
INSTRUM av500
PROBHD 5 mm DCH 13C-1
PULPROG zg30
TD 65536
SOLVENT CDCl3
NS 8
DS 0
SWH 10000.000 Hz
FIDRES 0.152588 Hz
AQ 3.2767999 sec
RG 12.14
DW 50.000 usec
DE 10.00 usec
TE 298.0 K
D1 2.00000000 sec
TD0 1

======== CHANNEL f1 =====
SFO1 500.1330008 MHz
NUC1 1H
P1 10.00 usec
PLW1 13.50000000 W

F2 - Processing parameters
SI 65536
SF 500.1300121 MHz
WDW EM
SSB 0
LB 0.30 Hz
GB 0
PC 1.00

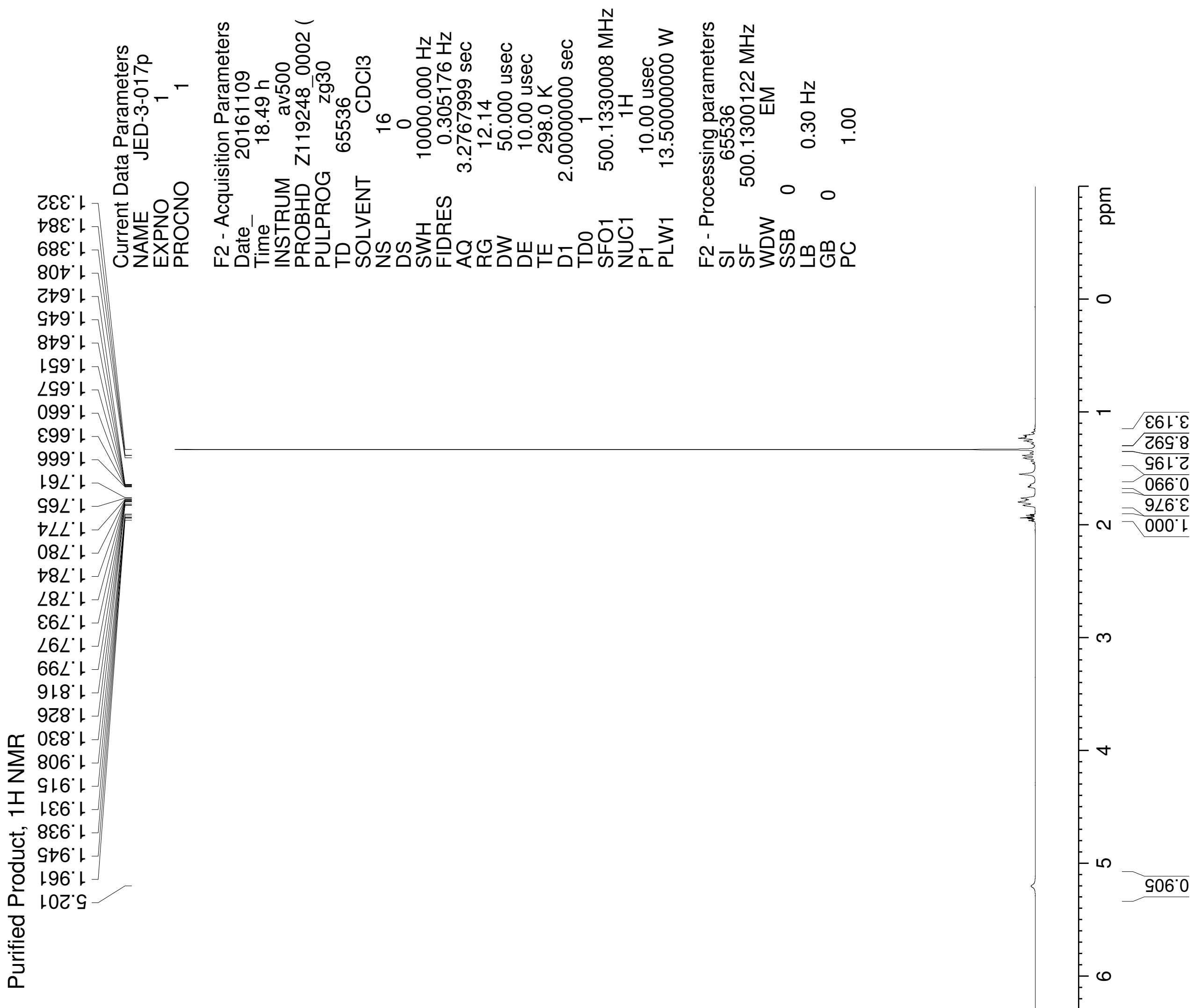
Purified Product, 1H NMR
5.201
1.961
1.945
1.938
1.931
1.915
1.908
1.830
1.826
1.816
1.799
1.797
1.793
1.787
1.784
1.780
1.774
1.765
1.761
1.666
1.663
1.660
1.657
1.651
1.648
1.645
1.642
1.408
1.389
1.384
1.332
Current Data Parameters
NAME JED-3-017p
EXPNO 1
PROCNO 1
F2 - Acquisition Parameters
Date_ 20161109
Time_ 18.49 h
INSTRUM av500
PROBHD Z119248_0002 (
PULPROG zg30
TD 65536
SOLVENT CDCl3
NS 16
DS 0
SWH 10000.000 Hz
FIDRES 0.305176 Hz
AQ 3.2767999 sec
RG 12.14
DW 50.000 usec
DE 10.00 usec
TE 298.0 K
D1 2.00000000 sec
TD0 1
SFO1 500.1330008 MHz
NUC1 1H
P1 10.00 usec
PLW1 13.50000000 W
F2 - Processing parameters
SI 65536
SF 500.1300122 MHz
WDW EM
SSB 0
LB 0.30 Hz
GB 0
PC 1.00
0.905
1.000
3.976
0.990
2.195
8.592
3.193
ppm

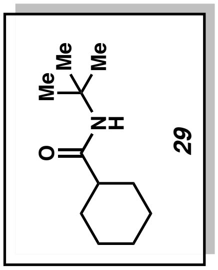
O
N
H
Me
Me
Me
29

Purified Product, 1H NMR

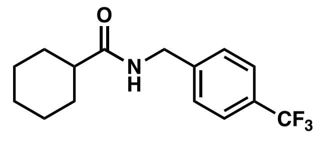

30

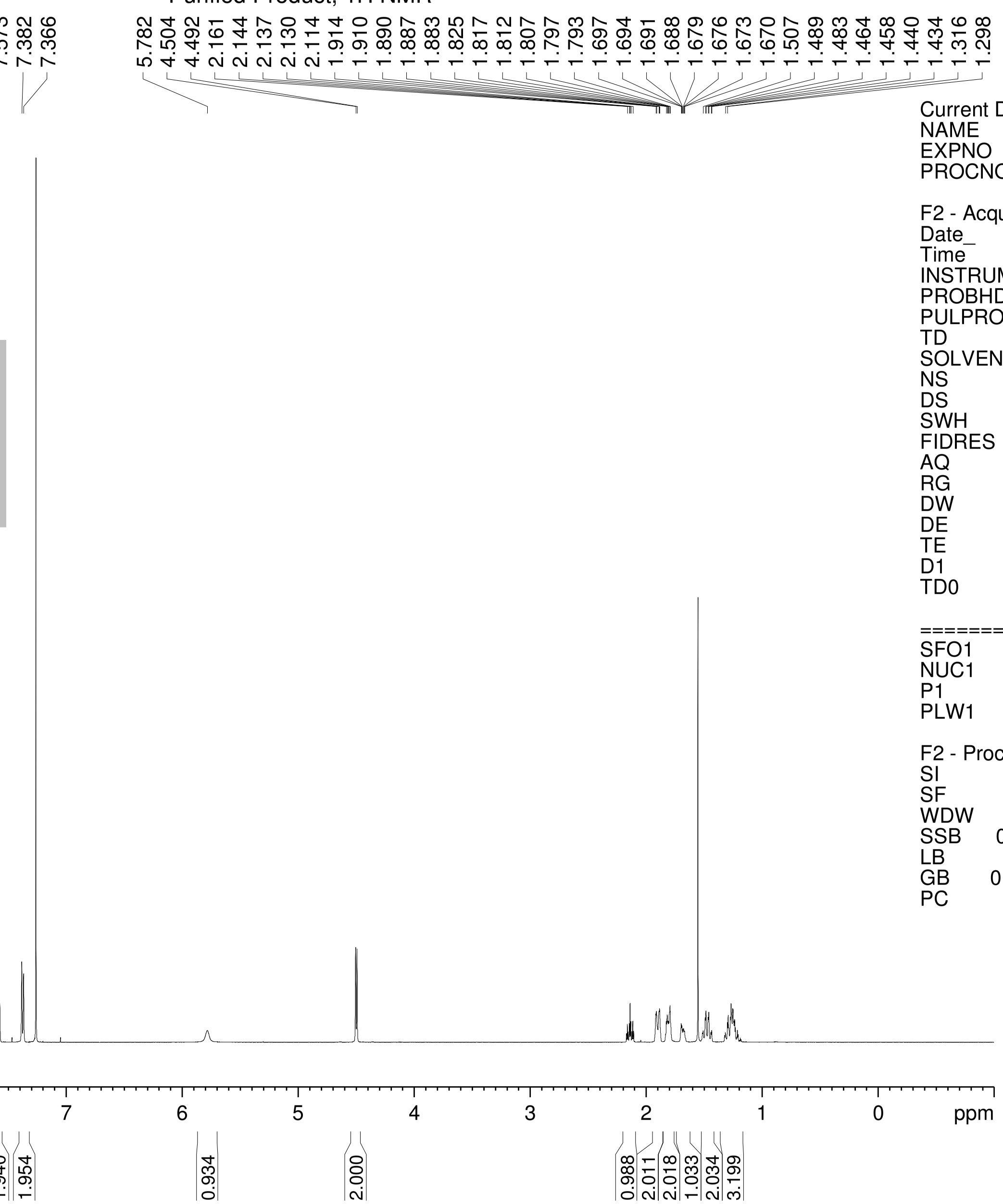

Current Data Parameters
NAME ELB-7-182p
EXPNO 1
PROCNO 1

F2 - Acquisition Parameters
Date_ 20170111
Time 16.52
INSTRUM av500
PROBHD 5 mm DCH 13C-1
PULPROG zg30
TD 65536
SOLVENT CDCl3
NS 8
DS 0
SWH 10000.000 Hz
FIDRES 0.152588 Hz
AQ 3.2767999 sec
RG 12.14
DW 50.000 usec
DE 10.00 usec
TE 298.0 K
D1 2.00000000 sec
TD0 1

======== CHANNEL f1 =====
SFO1 500.1330008 MHz
NUC1 1H
P1 10.00 usec
PLW1 13.50000000 W

F2 - Processing parameters
SI 65536
SF 500.1300122 MHz
WDW EM
SSB 0
LB 0.30 Hz
GB 0
PC 1.00

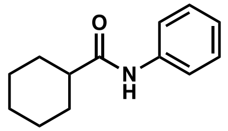

31a

Purified Product, 1H NMR

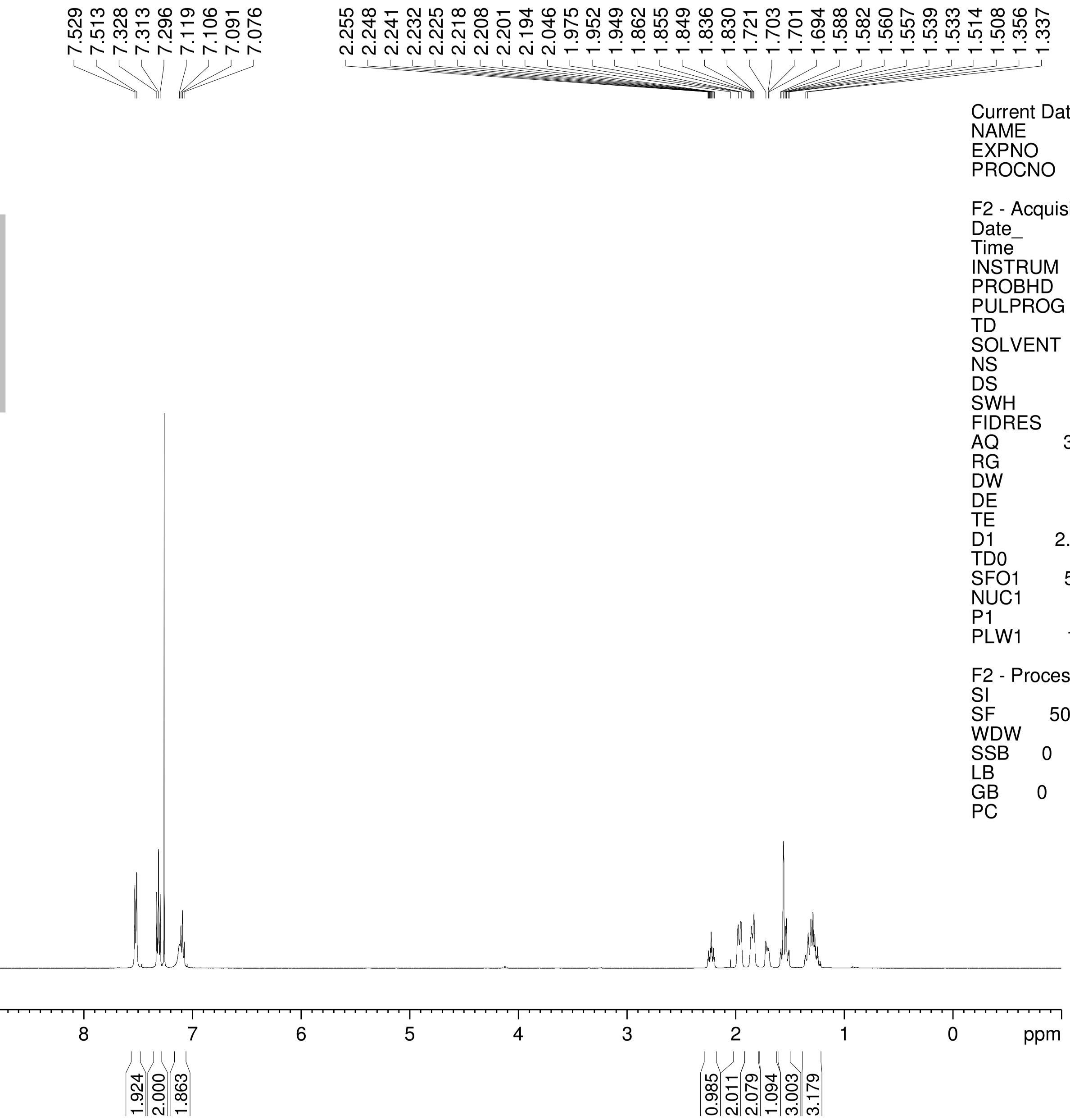

Current Data Parameters
NAME ELB-8-059p
EXPNO 2
PROCNO 1

F2 - Acquisition Parameters
Date_ 20170619
Time 14.09 h
INSTRUM av500
PROBHD Z119248_0002 (
PULPROG zg30
TD 65536
SOLVENT CDCl3
NS 8
DS 0
SWH 10000.000 Hz
FIDRES 0.305176 Hz
AQ 3.2767999 sec
RG 26.59
DW 50.000 usec
DE 10.00 usec
TE 298.0 K
D1 2.00000000 sec
TD0 1
SFO1 500.1330008 MHz
NUC1 1H
P1 10.00 usec
PLW1 13.50000000 W

F2 - Processing parameters
SI 65536
SF 500.1300121 MHz
WDW EM
SSB 0
LB 0.30 Hz
GB 0
PC 1.00

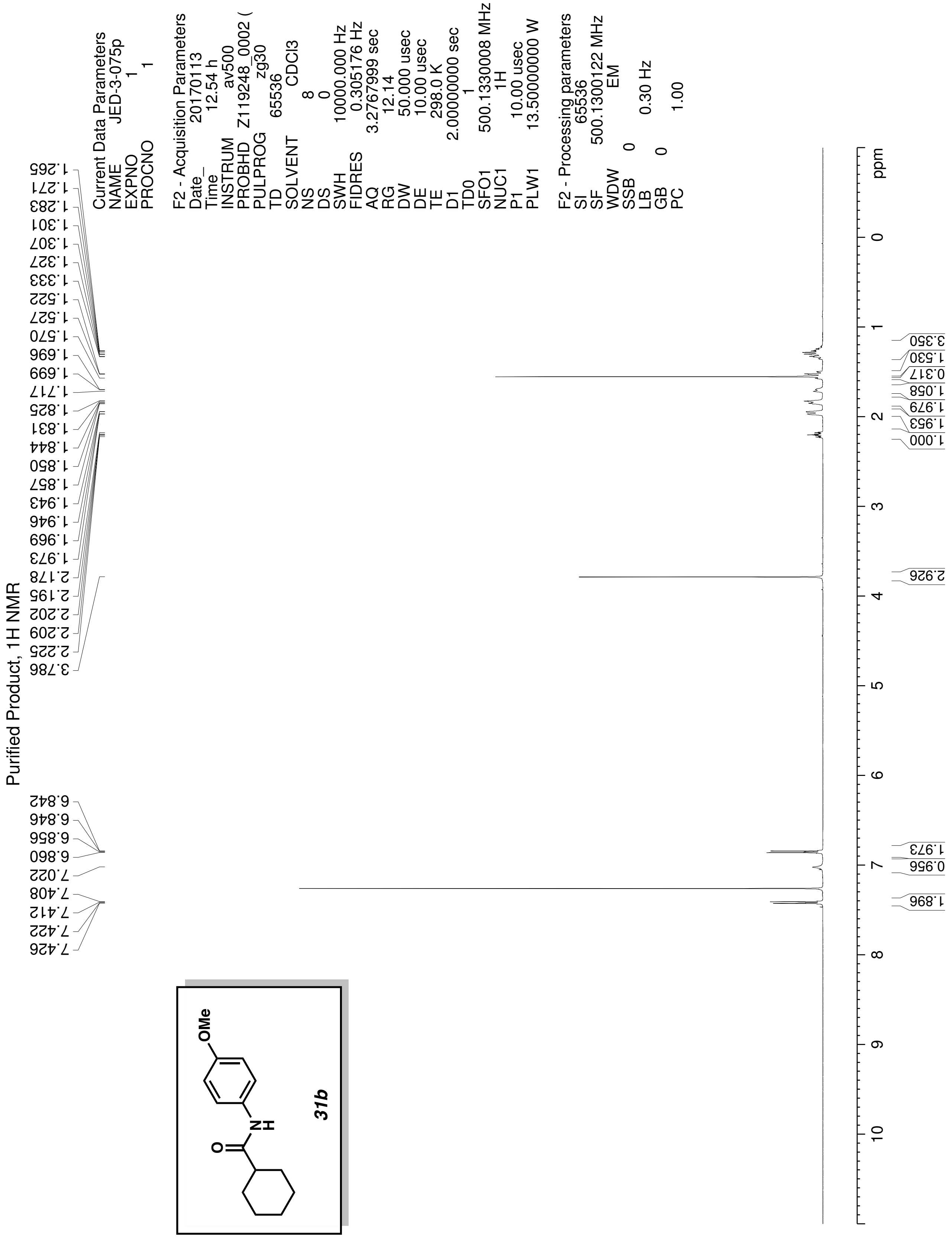
Purified Product, 1H NMR
7.426
7.422
7.412
7.408
7.022
6.860
6.856
6.846
6.842
3.786
2.225
2.209
2.202
2.195
2.178
1.973
1.969
1.946
1.943
1.857
1.850
1.844
1.831
1.825
1.717
1.699
1.696
1.570
1.527
1.522
1.333
1.327
1.307
1.301
1.283
1.271
1.265
Current Data Parameters
NAME JED-3-075p
EXPNO 1
PROCNO 1
F2 - Acquisition Parameters
Date_ 20170113
Time 12.54 h
INSTRUM av500
PROBHD Z119248_0002 (
PULPROG zg30
TD 65536
SOLVENT CDCl3
NS 8
DS 0
SWH 10000.000 Hz
FIDRES 0.305176 Hz
AQ 3.2767999 sec
RG 12.14
DW 50.000 usec
DE 10.00 usec
TE 298.0 K
D1 2.00000000 sec
TD0 1
SFO1 500.1330008 MHz
NUC1 1H
P1 10.00 usec
PLW1 13.50000000 W
F2 - Processing parameters
SI 65536
SF 500.1300122 MHz
WDW EM
SSB 0
LB 0.30 Hz
GB 0
PC 1.00
OMe
H
N
O
31b
1.896
0.956
1.973
2.926
1.000
1.953
1.979
1.058
0.317
1.530
3.350
ppm
10
9
8
7
6
5
4
3
2
1
0

Purified Product, 1H NMR

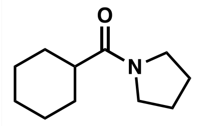

32

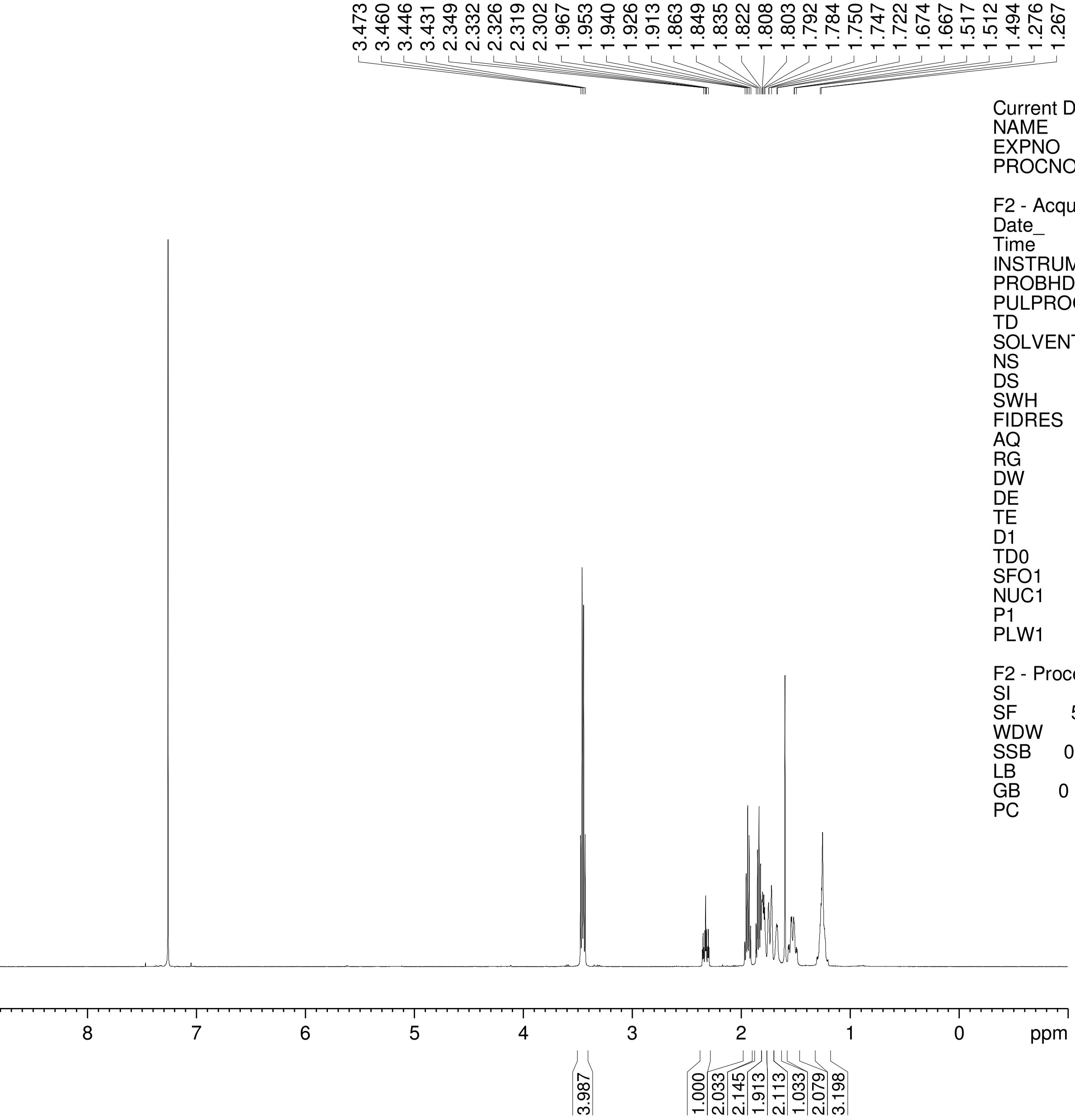

Current Data Parameters
NAME JED-3-033p
EXPNO 4
PROCNO 1

F2 - Acquisition Parameters
Date_ 20161129
Time 9.06 h
INSTRUM av500
PROBHD Z119248_0002 (
PULPROG zg30
TD 65536
SOLVENT CDCl3
NS 16
DS 0
SWH 10000.000 Hz
FIDRES 0.305176 Hz
AQ 3.2767999 sec
RG 12.14
DW 50.000 usec
DE 10.00 usec
TE 298.0 K
D1 2.00000000 sec
TD0 1
SFO1 500.1330008 MHz
NUC1 1H
P1 10.00 usec
PLW1 13.50000000 W

F2 - Processing parameters
SI 65536
SF 500.1300125 MHz
WDW EM
SSB 0
LB 0.30 Hz
GB 0
PC 1.00

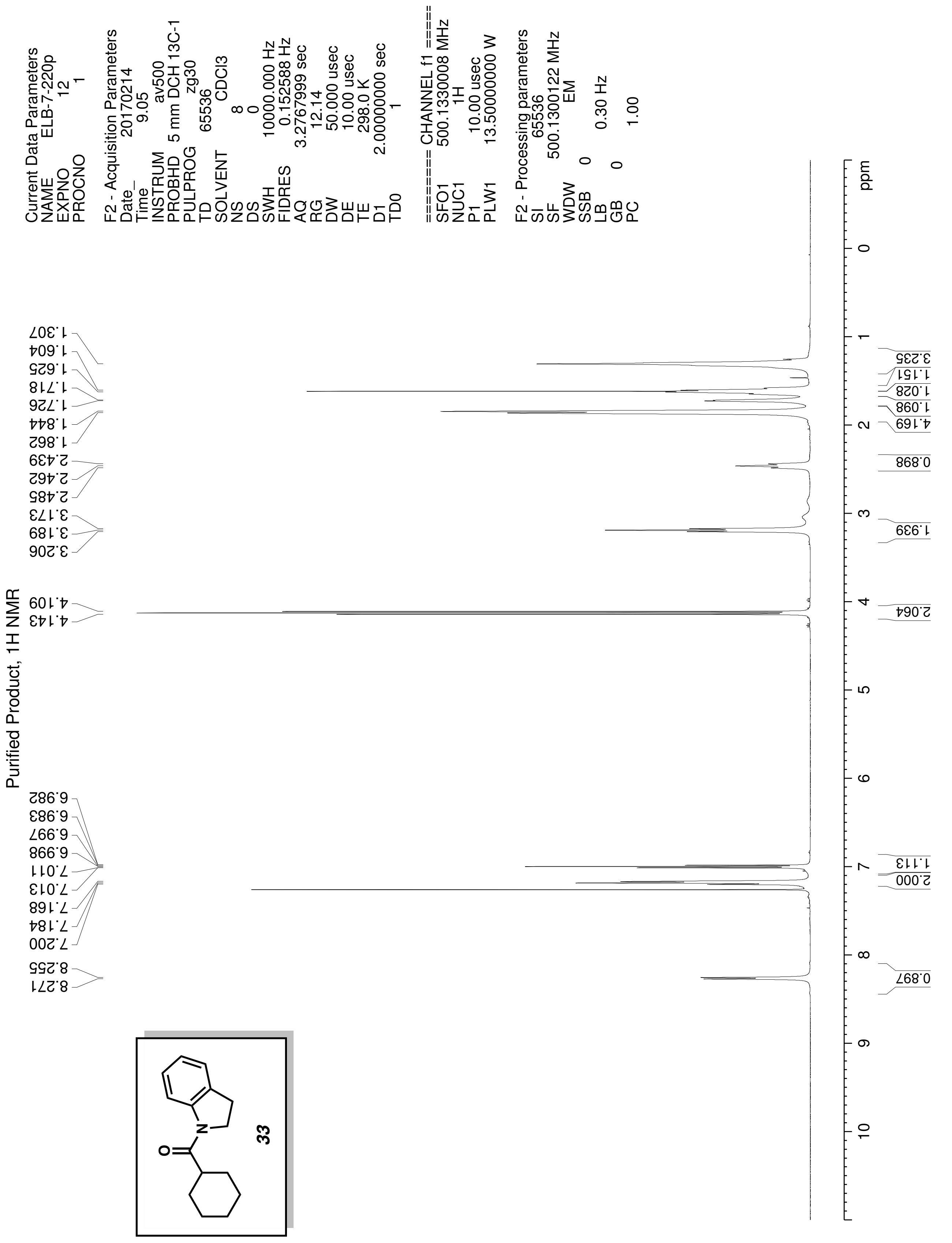
Purified Product, 1H NMR
8.271
8.255
7.200
7.184
7.168
7.013
7.011
6.998
6.997
6.983
6.982
4.143
4.109
3.206
3.189
3.173
2.485
2.462
2.439
1.862
1.844
1.726
1.718
1.625
1.604
1.307
Current Data Parameters
NAME ELB-7-220p
EXPNO 12
PROCNO 1
F2 - Acquisition Parameters
Date_ 20170214
Time 9.05
INSTRUM av500
PROBHD 5 mm DCH 13C-1
PULPROG zg30
TD 65536
SOLVENT CDCl3
NS 8
DS 0
SWH 10000.000 Hz
FIDRES 0.152588 Hz
AQ 3.2767999 sec
RG 12.14
DW 50.000 usec
DE 10.00 usec
TE 298.0 K
D1 2.00000000 sec
TD0 1
======== CHANNEL f1 =====
SFO1 500.1330008 MHz
NUC1 1H
P1 10.00 usec
PLW1 13.50000000 W
F2 - Processing parameters
SI 65536
SF 500.1300122 MHz
WDW EM
SSB 0
LB 0.30 Hz
GB 0
PC 1.00
0.897
2.000
1.113
2.064
1.939
0.898
4.169
1.098
1.028
1.151
3.235
10
9
8
7
6
5
4
3
2
1
0
ppm
33

Purified Product, 1H NMR

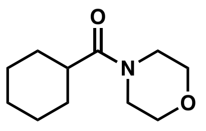

34

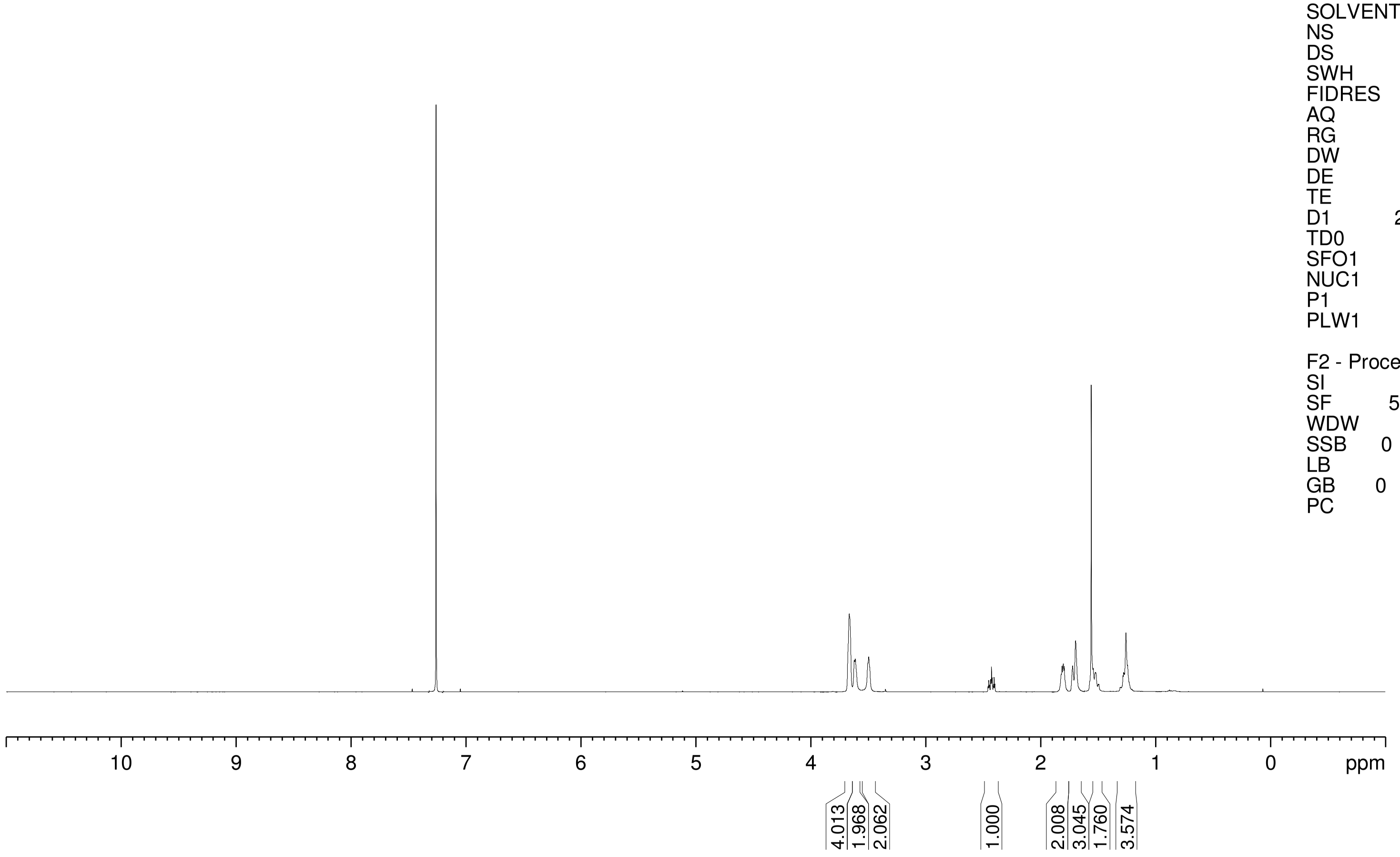

Current Data Parameters
NAME JED-3-073p
EXPNO 1
PROCNO 1

F2 - Acquisition Parameters
Date_ 20170113
Time 12.49 h
INSTRUM av500
PROBHD Z119248_0002 (
PULPROG zg30
TD 65536
SOLVENT CDCl3
NS 8
DS 0
SWH 10000.000 Hz
FIDRES 0.305176 Hz
AQ 3.2767999 sec
RG 12.14
DW 50.000 usec
DE 10.00 usec
TE 298.0 K
D1 2.00000000 sec
TD0 1
SFO1 500.1330008 MHz
NUC1 1H
P1 10.00 usec
PLW1 13.50000000 W

F2 - Processing parameters
SI 65536
SF 500.1300125 MHz
WDW EM
SSB 0
LB 0.30 Hz
GB 0
PC 1.00

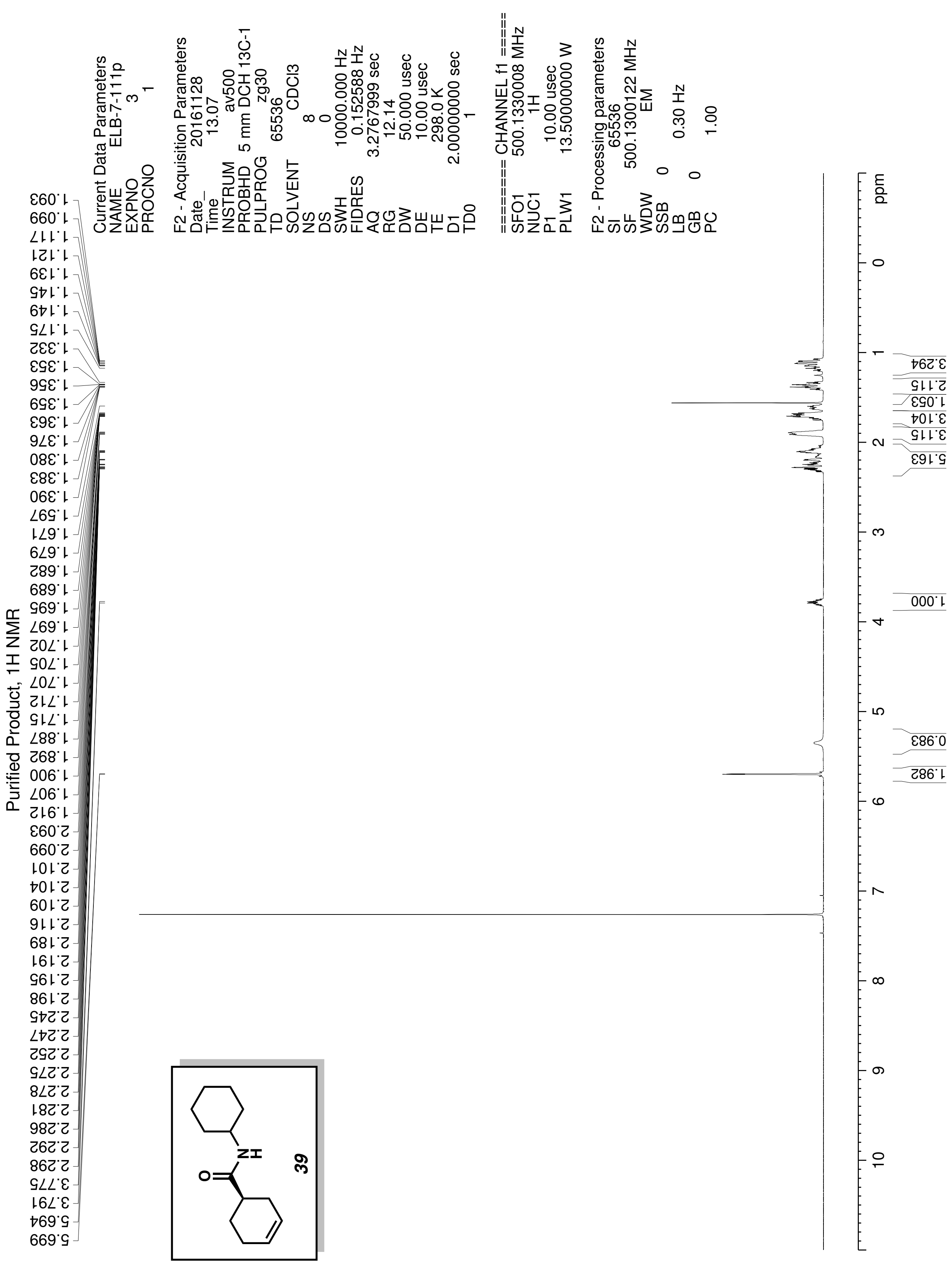
Purified Product, 1H NMR
5.699
5.694
3.791
3.775
2.298
2.292
2.286
2.281
2.278
2.275
2.252
2.247
2.245
2.198
2.195
2.191
2.189
2.116
2.109
2.104
2.101
2.099
2.093
1.912
1.907
1.900
1.892
1.887
1.715
1.712
1.707
1.705
1.702
1.697
1.695
1.689
1.682
1.679
1.671
1.597
1.390
1.383
1.380
1.376
1.363
1.359
1.356
1.353
1.332
1.175
1.149
1.145
1.139
1.121
1.117
1.099
1.093
Current Data Parameters
NAME ELB-7-111p
EXPNO 3
PROCNO 1
F2 - Acquisition Parameters
Date_ 20161128
Time 13.07
INSTRUM av500
PROBHD 5 mm DCH 13C-1
PULPROG zg30
TD 65536
SOLVENT CDCl3
NS 8
DS 0
SWH 10000.000 Hz
FIDRES 0.152588 Hz
AQ 3.2767999 sec
RG 12.14
DW 50.000 usec
DE 10.00 usec
TE 298.0 K
D1 2.00000000 sec
TD0 1
======== CHANNEL f1 ========
SFO1 500.1330008 MHz
NUC1 1H
P1 10.00 usec
PLW1 13.50000000 W
F2 - Processing parameters
SI 65536
SF 500.1300122 MHz
WDW EM
SSB 0
LB 0.30 Hz
GB 0
PC 1.00
1.982
0.983
1.000
5.163
3.115
3.104
1.053
2.115
3.294
10
9
8
7
6
5
4
3
2
1
0
ppm
O
N
H
39

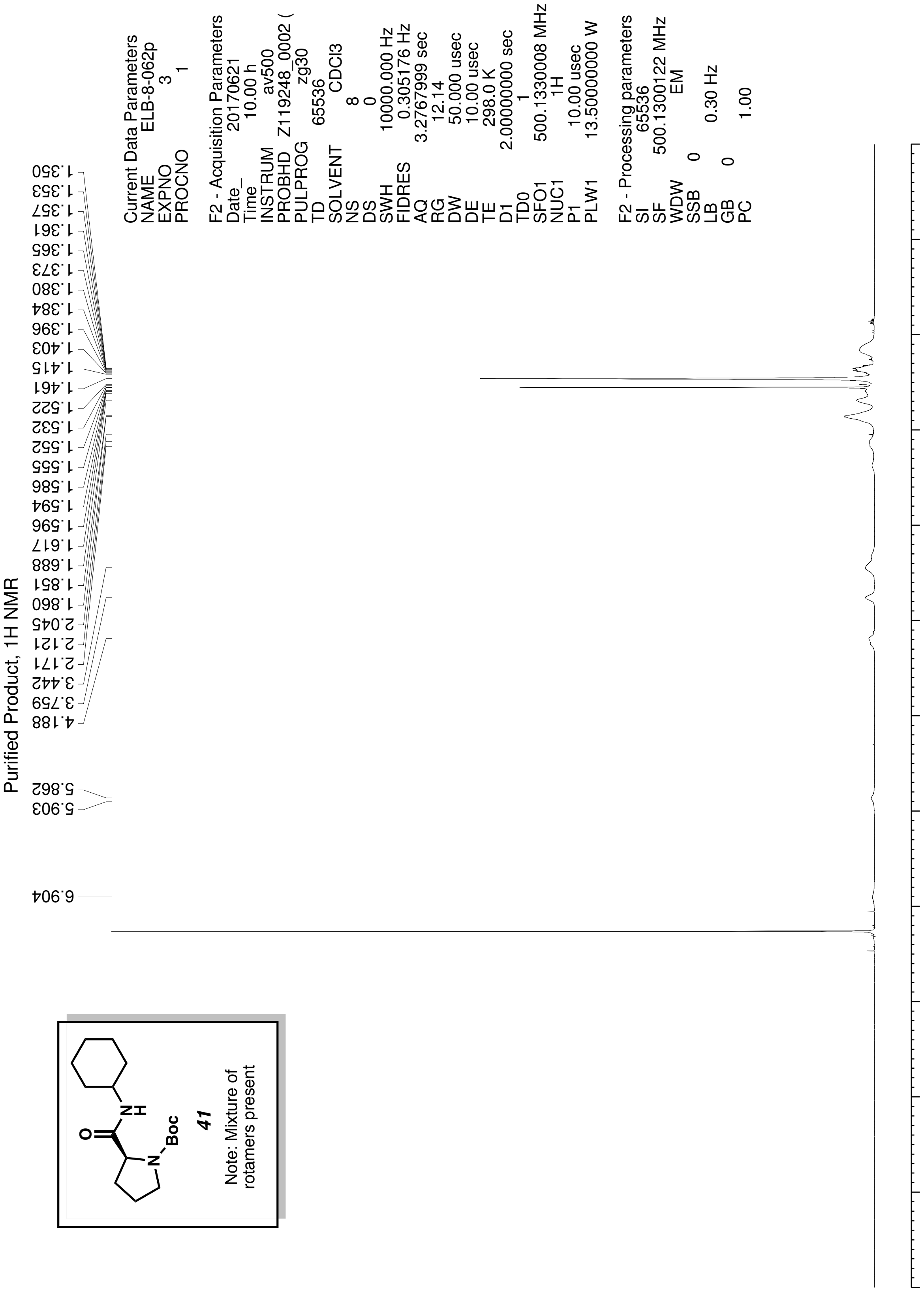
Purified Product, 1H NMR
6.904
5.903
5.862
4.188
3.759
3.442
2.171
2.121
2.045
1.860
1.851
1.688
1.617
1.596
1.594
1.586
1.555
1.552
1.532
1.522
1.461
1.415
1.403
1.396
1.384
1.380
1.373
1.365
1.361
1.357
1.353
1.350
Current Data Parameters
NAME ELB-8-062p
EXPNO 3
PROCNO 1
F2 - Acquisition Parameters
Date_ 20170621
Time 10.00 h
INSTRUM av500
PROBHD Z119248_0002 (
PULPROG zg30
TD 65536
SOLVENT CDCl3
NS 8
DS 0
SWH 10000.000 Hz
FIDRES 0.305176 Hz
AQ 3.2767999 sec
RG 12.14
DW 50.000 usec
DE 10.00 usec
TE 298.0 K
D1 2.00000000 sec
TD0 1
SFO1 500.1330008 MHz
NUC1 1H
P1 10.00 usec
PLW1 13.50000000 W
F2 - Processing parameters
SI 65536
SF 500.1300122 MHz
WDW EM
SSB 0
LB 0.30 Hz
GB 0
PC 1.00
0.396
0.500
1.007
1.002
2.032
1.357
7.731
9.134
5.054
10
9
8
7
6
5
4
3
2
1
0
ppm
O
N
H
N
Boc
41
Note: Mixture of rotamers present

^{13}C NMR Spectra

Purified Product, 13C NMR

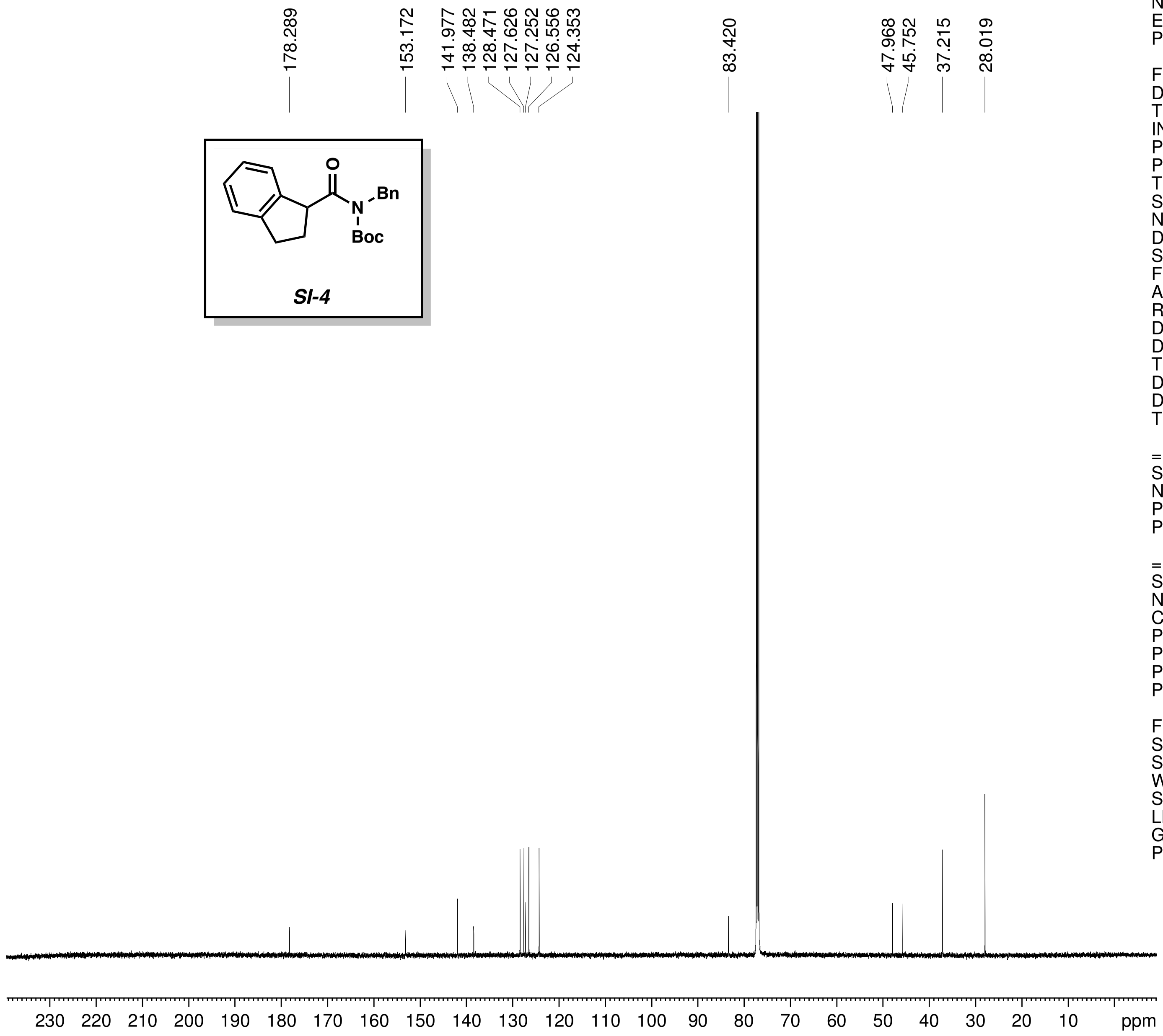

Current Data Parameters
NAME ELB-7-051p
EXPNO 5
PROCNO 1

F2 - Acquisition Parameters
Date_ 20161127
Time 11.43
INSTRUM av500
PROBHD 5 mm DCH 13C-1
PULPROG zgpg30
TD 65536
SOLVENT CDCl3
NS 108
DS 2
SWH 31250.000 Hz
FIDRES 0.476837 Hz
AQ 1.0485760 sec
RG 204.54
DW 16.000 usec
DE 18.00 usec
TE 298.0 K
D1 2.00000000 sec
D11 0.03000000 sec
TD0 1

======== CHANNEL f1 =====
SFO1 125.7722511 MHz
NUC1 13C
P1 9.63 usec
PLW1 23.00000000 W

======== CHANNEL f2 =====
SFO2 500.1330008 MHz
NUC2 1H
CPDPRG[2 waltz16
PCPD2 80.00 usec
PLW2 13.50000000 W
PLW12 0.21094000 W
PLW13 0.13500001 W

F2 - Processing parameters
SI 131072
SF 125.7577730 MHz
WDW EM
SSB 0
LB 1.00 Hz
GB 0
PC 1.40

Purified Product, 13C NMR

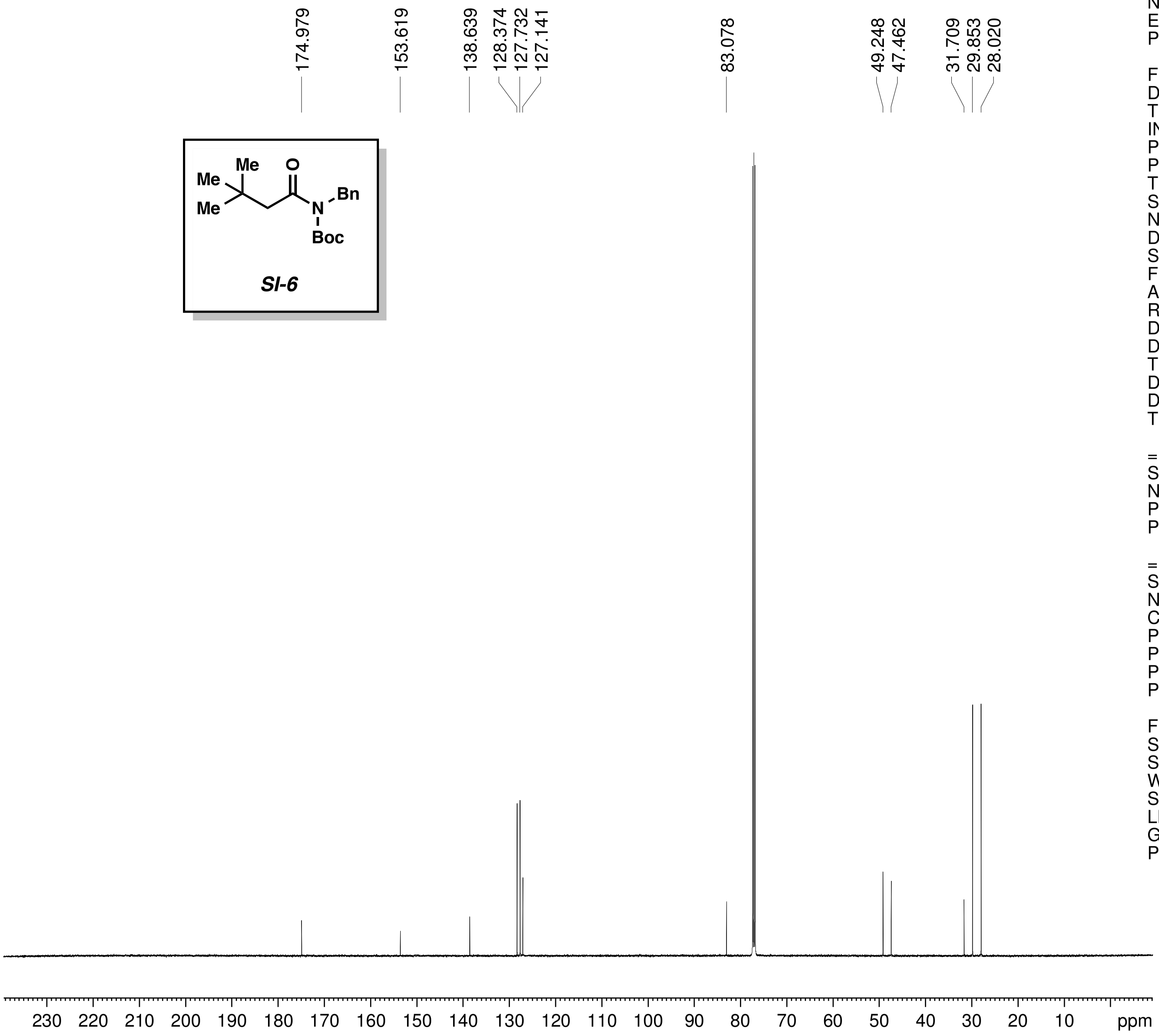

Current Data Parameters
NAME ELB-7-123p
EXPNO 3
PROCNO 1

F2 - Acquisition Parameters
Date_ 20161207
Time 9.24
INSTRUM av500
PROBHD 5 mm DCH 13C-1
PULPROG zgpg30
TD 65536
SOLVENT CDCl3
NS 50
DS 2
SWH 31250.000 Hz
FIDRES 0.476837 Hz
AQ 1.0485760 sec
RG 204.54
DW 16.000 usec
DE 18.00 usec
TE 298.0 K
D1 2.00000000 sec
D11 0.03000000 sec
TD0 1

======== CHANNEL f1 =====
SFO1 125.7722511 MHz
NUC1 13C
P1 9.63 usec
PLW1 23.00000000 W

======== CHANNEL f2 =====
SFO2 500.1330008 MHz
NUC2 1H
CPDPRG[2 waltz16
PCPD2 80.00 usec
PLW2 13.50000000 W
PLW12 0.21094000 W
PLW13 0.13500001 W

F2 - Processing parameters
SI 131072
SF 125.7577726 MHz
WDW EM
SSB 0
LB 1.00 Hz
GB 0
PC 1.40

Purified Product, 13C NMR

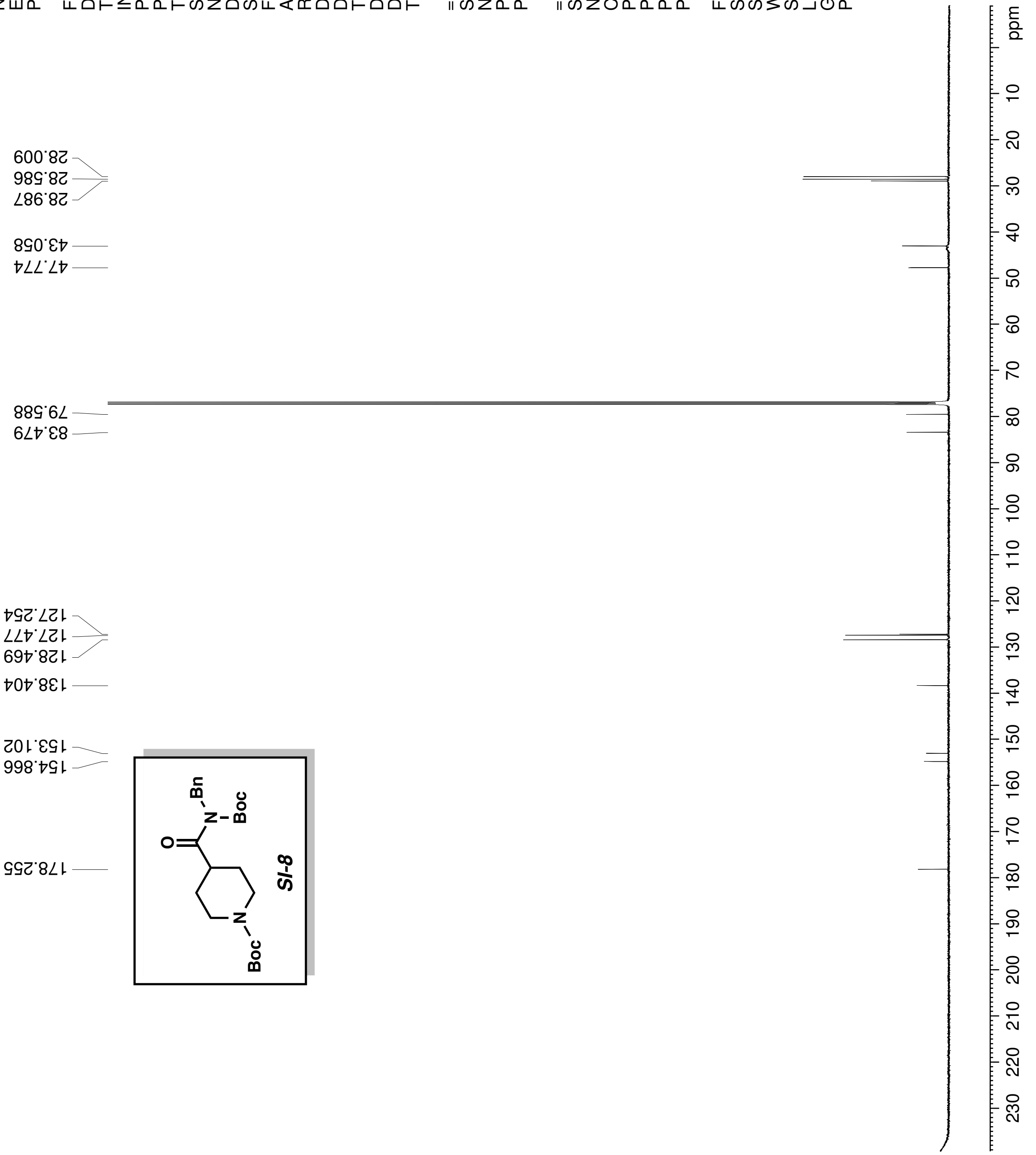

Current Data Parameters
NAME TBB-1-087p
EXPNO 2
PROCNO 1

F2 - Acquisition Parameters
Date_ 20161127
Time 11.56
INSTRUM av500
PROBHD 5 mm DCH 13C-1
PULPROG zgpg30
TD 65536
SOLVENT CDCl3
NS 108
DS 2
SWH 31250.000 Hz
FIDRES 0.476837 Hz
AQ 1.0485760 sec
RG 204.54
DW 16.000 usec
DE 18.00 usec
TE 298.0 K
D1 2.00000000 sec
D11 0.03000000 sec
TD0 1

======== CHANNEL f1 ========
SFO1 125.7722511 MHz
NUC1 13C
P1 9.63 usec
PLW1 23.00000000 W

======== CHANNEL f2 ========
SFO2 500.1330008 MHz
NUC2 1H
CPDPRG[2 waltz16
PCPD2 80.00 usec
PLW2 13.50000000 W
PLW12 0.21094000 W
PLW13 0.13500001 W

F2 - Processing parameters
SI 131072
SF 125.7577735 MHz
WDW EM
SSB 0
LB 1.00 Hz
GB 0
PC 1.40

Purified Product, 13C NMR

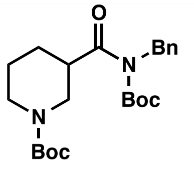

SI-10

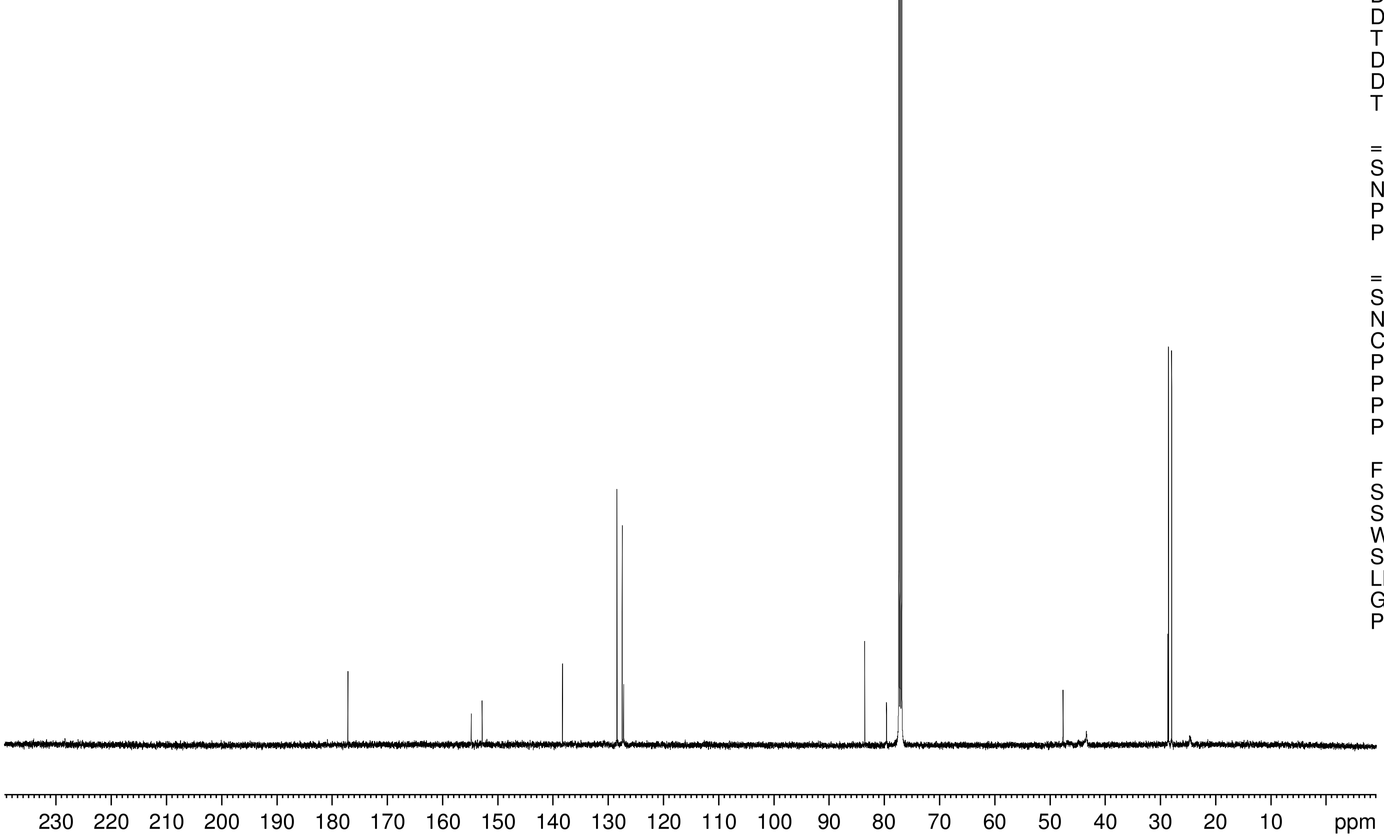

Current Data Parameters
NAME TBB-1-082p
EXPNO 2
PROCNO 1

F2 - Acquisition Parameters
Date_ 20161127
Time 12.21
INSTRUM av500
PROBHD 5 mm DCH 13C-1
PULPROG zgpg30
TD 65536
SOLVENT CDCl3
NS 106
DS 2
SWH 31250.000 Hz
FIDRES 0.476837 Hz
AQ 1.0485760 sec
RG 204.54
DW 16.000 usec
DE 18.00 usec
TE 298.0 K
D1 2.00000000 sec
D11 0.03000000 sec
TD0 1

======== CHANNEL f1 =====
SFO1 125.7722511 MHz
NUC1 13C
P1 9.63 usec
PLW1 23.00000000 W

======== CHANNEL f2 =====
SFO2 500.1330008 MHz
NUC2 1H
CPDPRG[2 waltz16
PCPD2 80.00 usec
PLW2 13.50000000 W
PLW12 0.21094000 W
PLW13 0.13500001 W

F2 - Processing parameters
SI 131072
SF 125.7577721 MHz
WDW EM
SSB 0
LB 1.00 Hz
GB 0
PC 1.40

Purified Product, 13C NMR

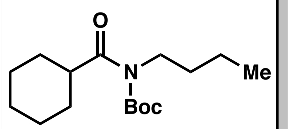

22

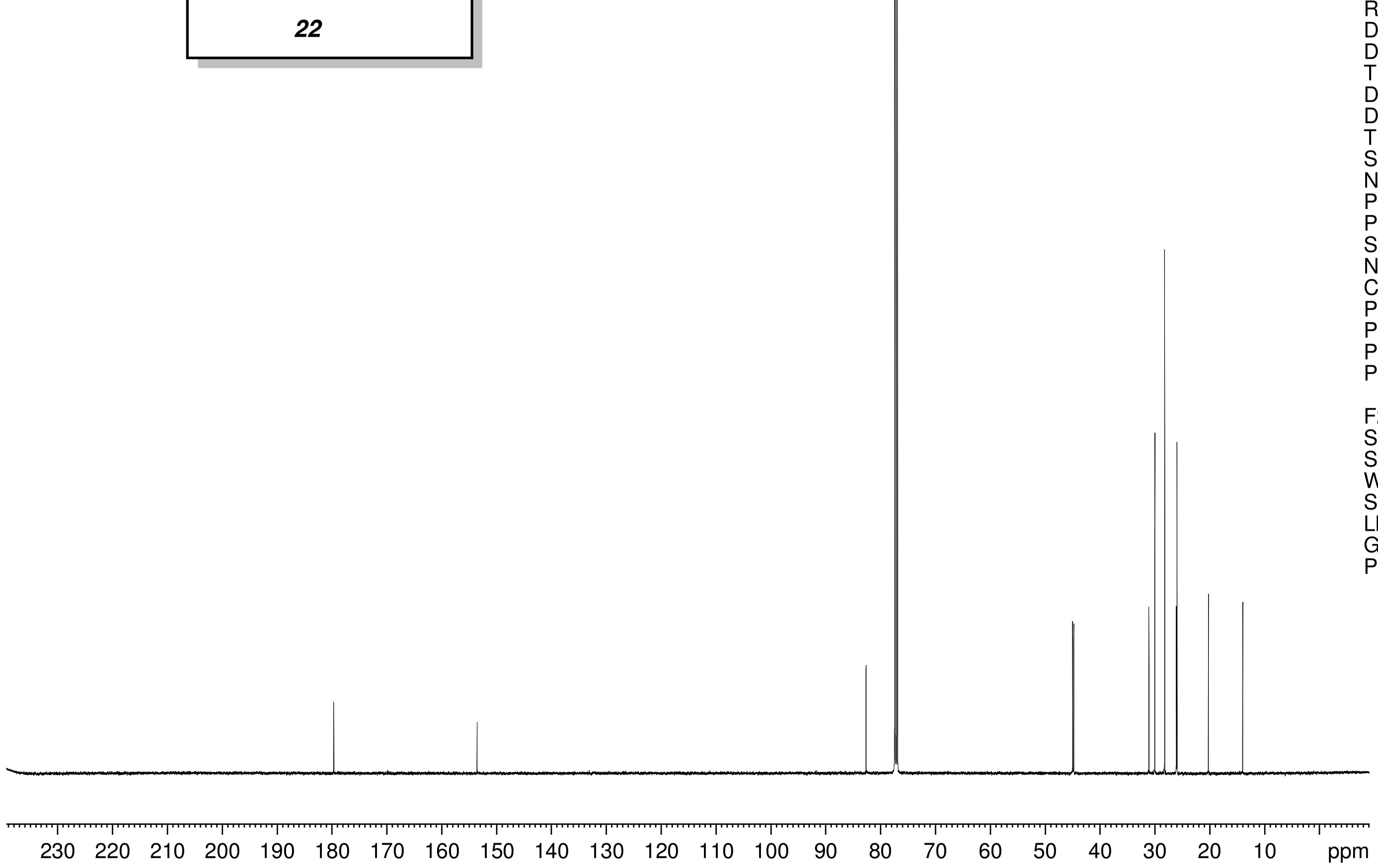

Current Data Parameters
NAME ELB-7-298p
EXPNO 10
PROCNO 1

F2 - Acquisition Parameters
Date_ 20170420
Time 13.14 h
INSTRUM av500
PROBHD Z119248_0002 (
PULPROG zgpg30
TD 65536
SOLVENT CDCl3
NS 41
DS 2
SWH 31250.000 Hz
FIDRES 0.953674 Hz
AQ 1.0485760 sec
RG 204.54
DW 16.000 usec
DE 18.00 usec
TE 298.0 K
D1 2.00000000 sec
D11 0.03000000 sec
TD0 1
SFO1 125.7722511 MHz
NUC1 13C
P1 10.50 usec
PLW1 23.00000000 W
SFO2 500.1330008 MHz
NUC2 1H
CPDPRG[2 waltz16
PCPD2 80.00 usec
PLW2 13.50000000 W
PLW12 0.21094000 W
PLW13 0.10610000 W

F2 - Processing parameters
SI 131072
SF 125.7577720 MHz
WDW EM
SSB 0
LB 1.00 Hz
GB 0
PC 1.40

Purified Product, 13C NMR

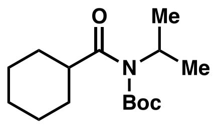

23

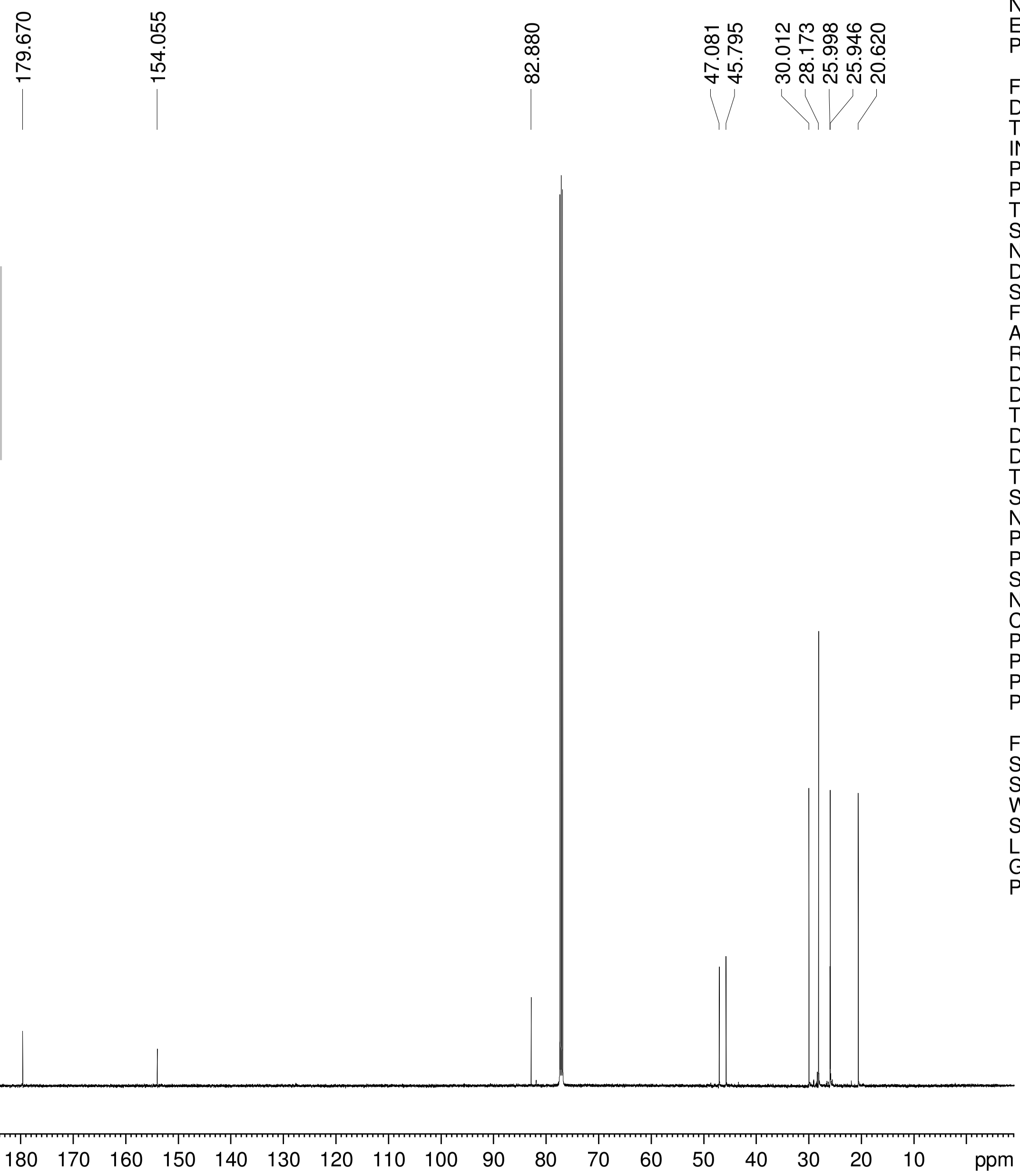

Current Data Parameters

NAME	ELB-8-018p
EXPNO	12
PROCNO	1

F2 - Acquisition Parameters

Date_	20170420
Time	15.09 h
INSTRUM	av500
PROBHD	Z119248_0002 (
PULPROG	zgpg30
TD	65536
SOLVENT	CDCl3
NS	61
DS	2
SWH	31250.000 Hz
FIDRES	0.953674 Hz
AQ	1.0485760 sec
RG	204.54
DW	16.000 usec
DE	18.00 usec
TE	298.0 K
D1	2.00000000 sec
D11	0.03000000 sec
TD0	1
SFO1	125.7722511 MHz
NUC1	13C
P1	10.50 usec
PLW1	23.00000000 W
SFO2	500.1330008 MHz
NUC2	1H
CPDPRG[2	waltz16
PCPD2	80.00 usec
PLW2	13.50000000 W
PLW12	0.21094000 W
PLW13	0.10610000 W

F2 - Processing parameters

SI	131072
SF	125.7577725 MHz
WDW	EM
SSB	0
LB	1.00 Hz
GB	0
PC	1.40

Purified Product, 13C NMR

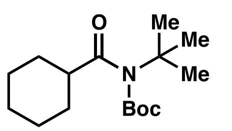

24

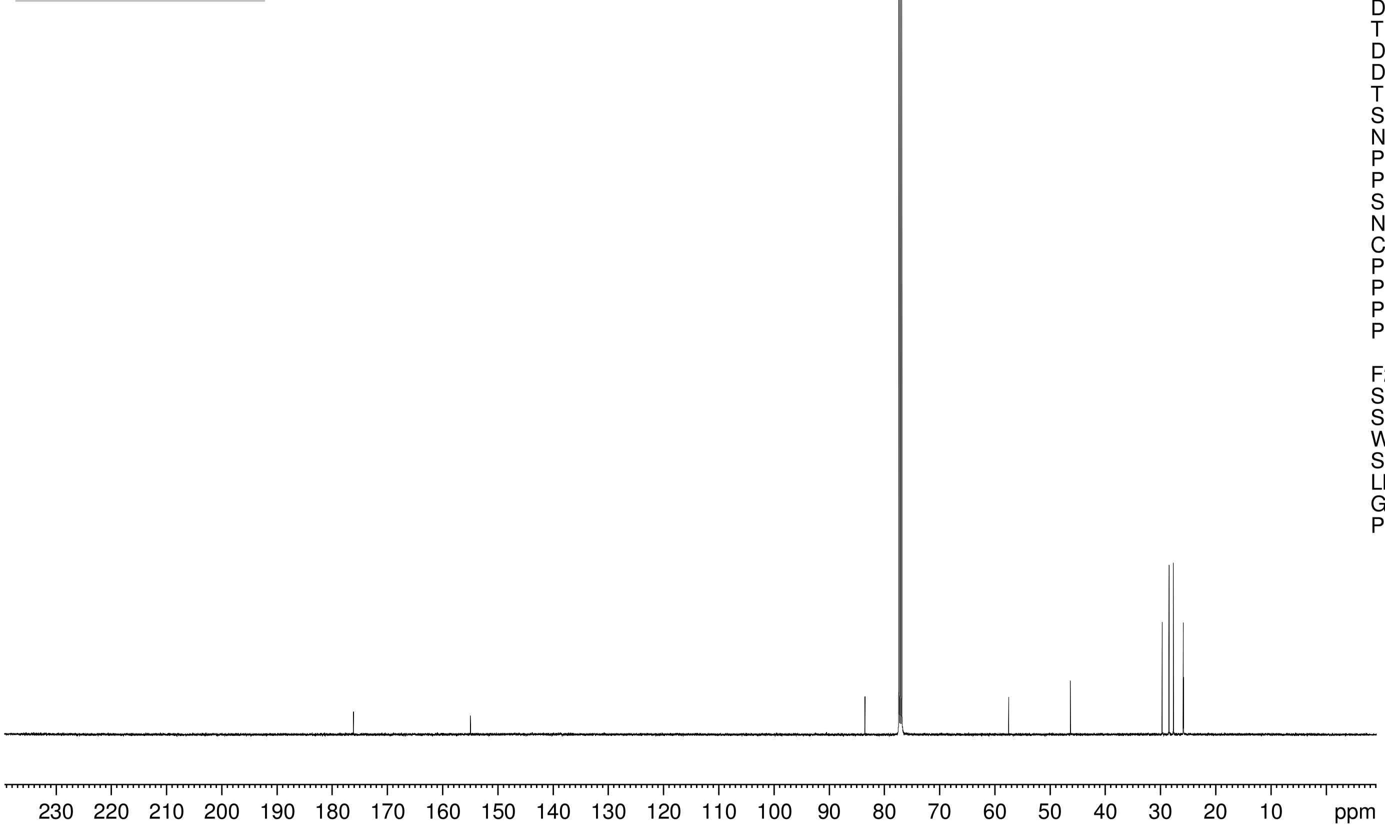

Current Data Parameters
NAME JED-3-152p
EXPNO 2
PROCNO 1

F2 - Acquisition Parameters
Date_ 20170501
Time 13.01 h
INSTRUM av500
PROBHD Z119248_0002 (
PULPROG zgpg30
TD 65536
SOLVENT CDCl3
NS 108
DS 2
SWH 31250.000 Hz
FIDRES 0.953674 Hz
AQ 1.0485760 sec
RG 204.54
DW 16.000 usec
DE 18.00 usec
TE 298.0 K
D1 2.00000000 sec
D11 0.03000000 sec
TD0 1
SFO1 125.7722511 MHz
NUC1 13C
P1 10.50 usec
PLW1 23.00000000 W
SFO2 500.1330008 MHz
NUC2 1H
CPDPRG[2 waltz16
PCPD2 80.00 usec
PLW2 13.50000000 W
PLW12 0.21094000 W
PLW13 0.10610000 W

F2 - Processing parameters
SI 131072
SF 125.7577711 MHz
WDW EM
SSB 0
LB 1.00 Hz
GB 0
PC 1.40

Purified Product, 13C NMR

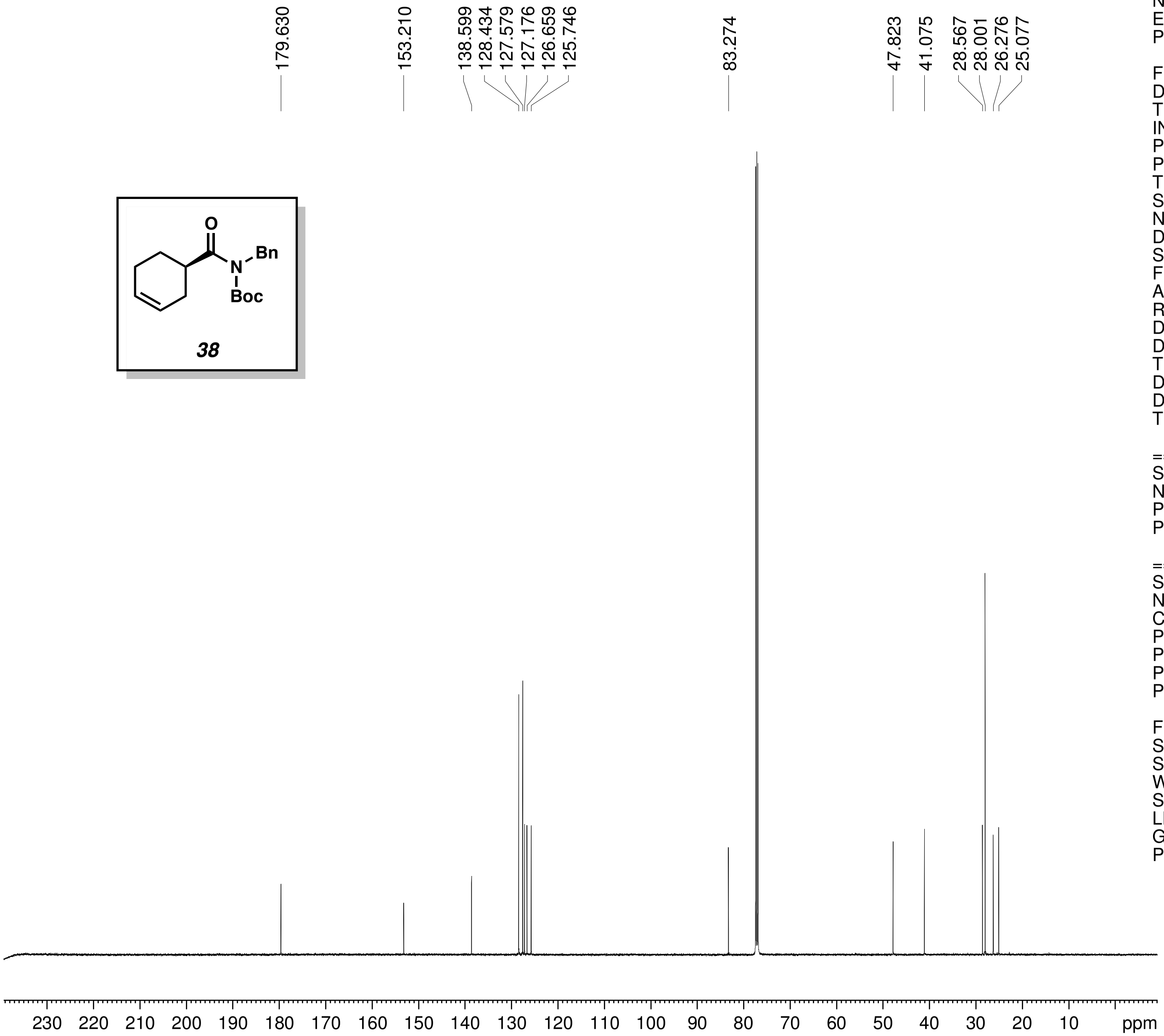

Current Data Parameters
NAME TBB-1-168p
EXPNO 2
PROCNO 1

F2 - Acquisition Parameters
Date_ 20161128
Time 15.22
INSTRUM av500
PROBHD 5 mm DCH 13C-1
PULPROG zgpg30
TD 65536
SOLVENT CDCl3
NS 108
DS 2
SWH 31250.000 Hz
FIDRES 0.476837 Hz
AQ 1.0485760 sec
RG 204.54
DW 16.000 usec
DE 18.00 usec
TE 298.0 K
D1 2.00000000 sec
D11 0.03000000 sec
TD0 1

======== CHANNEL f1 =====
SFO1 125.7722511 MHz
NUC1 13C
P1 9.63 usec
PLW1 23.00000000 W

======== CHANNEL f2 =====
SFO2 500.1330008 MHz
NUC2 1H
CPDPRG[2 waltz16
PCPD2 80.00 usec
PLW2 13.50000000 W
PLW12 0.21094000 W
PLW13 0.13500001 W

F2 - Processing parameters
SI 131072
SF 125.7577730 MHz
WDW EM
SSB 0
LB 1.00 Hz
GB 0
PC 1.40

Purified Product, 13C NMR

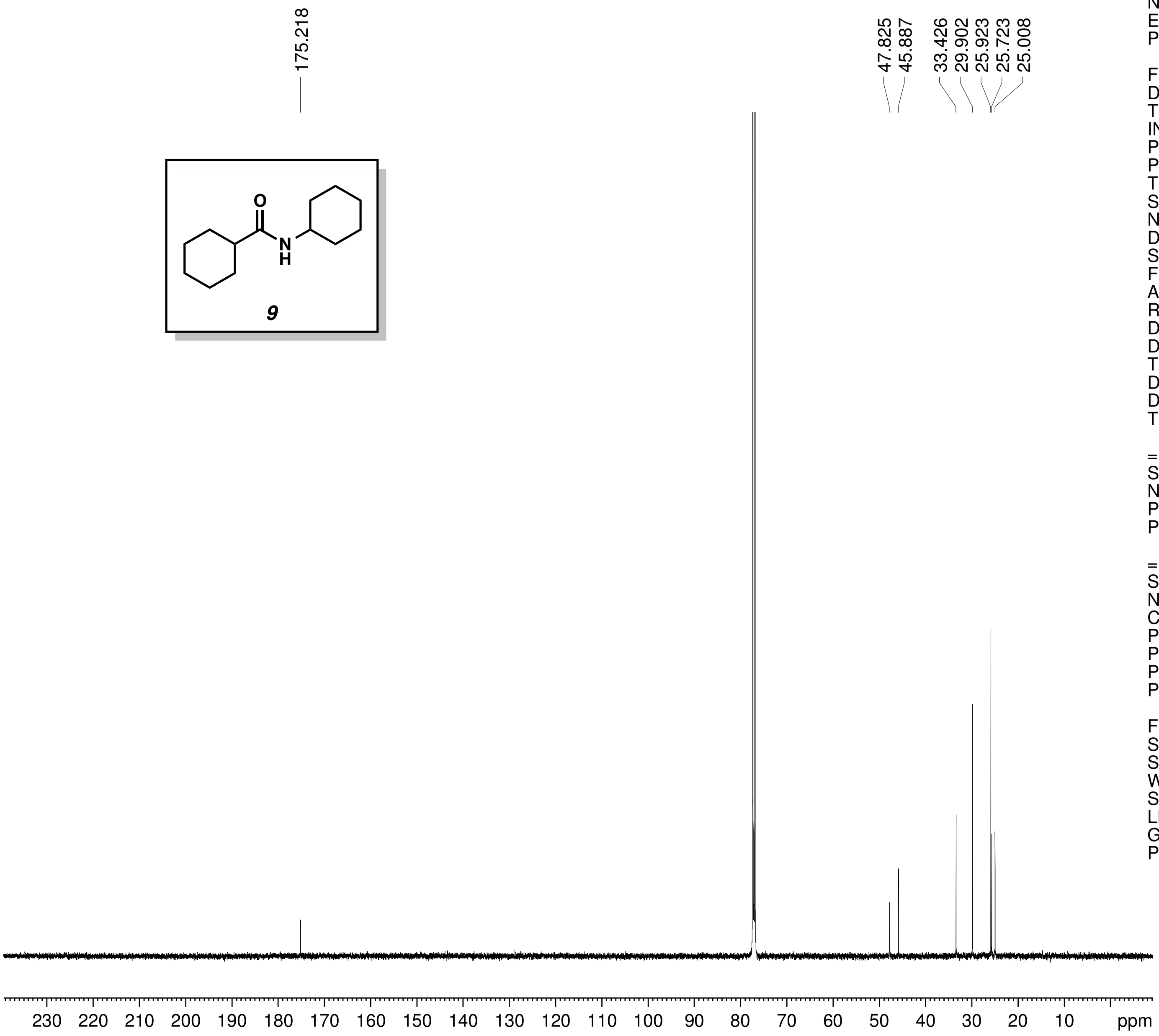

Current Data Parameters
NAME ELB-7-090p
EXPNO 2
PROCNO 1

F2 - Acquisition Parameters
Date_ 20161128
Time 10.18
INSTRUM av500
PROBHD 5 mm DCH 13C-1
PULPROG zgpg30
TD 65536
SOLVENT CDCl3
NS 108
DS 2
SWH 31250.000 Hz
FIDRES 0.476837 Hz
AQ 1.0485760 sec
RG 204.54
DW 16.000 usec
DE 18.00 usec
TE 298.0 K
D1 2.00000000 sec
D11 0.03000000 sec
TD0 1

======== CHANNEL f1 =====
SFO1 125.7722511 MHz
NUC1 13C
P1 9.63 usec
PLW1 23.00000000 W

======== CHANNEL f2 =====
SFO2 500.1330008 MHz
NUC2 1H
CPDPRG[2 waltz16
PCPD2 80.00 usec
PLW2 13.50000000 W
PLW12 0.21094000 W
PLW13 0.13500001 W

F2 - Processing parameters
SI 131072
SF 125.7577722 MHz
WDW EM
SSB 0
LB 1.00 Hz
GB 0
PC 1.40

Purified Product, 13C NMR

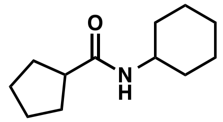

15

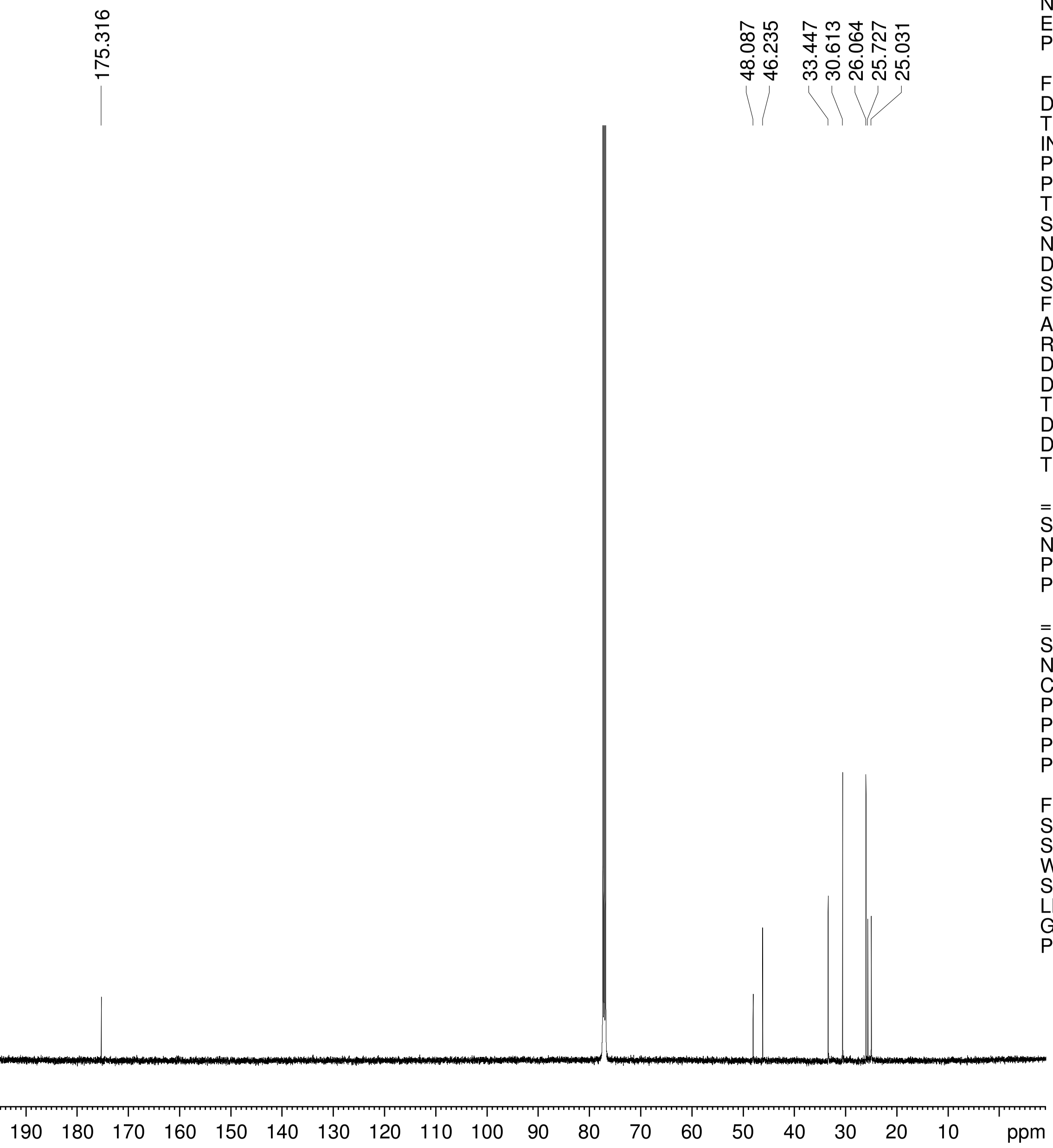

Current Data Parameters
NAME ELB-7-079p
EXPNO 3
PROCNO 1

F2 - Acquisition Parameters
Date_ 20161111
Time 13.41
INSTRUM av500
PROBHD 5 mm DCH 13C-1
PULPROG zgpg30
TD 65536
SOLVENT CDCl3
NS 108
DS 2
SWH 31250.000 Hz
FIDRES 0.476837 Hz
AQ 1.0485760 sec
RG 204.54
DW 16.000 usec
DE 18.00 usec
TE 298.0 K
D1 2.00000000 sec
D11 0.03000000 sec
TD0 1

======== CHANNEL f1 =====
SFO1 125.7722511 MHz
NUC1 13C
P1 9.63 usec
PLW1 23.00000000 W

======== CHANNEL f2 =====
SFO2 500.1330008 MHz
NUC2 1H
CPDPRG[2 waltz16
PCPD2 80.00 usec
PLW2 13.50000000 W
PLW12 0.21094000 W
PLW13 0.13500001 W

F2 - Processing parameters
SI 131072
SF 125.7577724 MHz
WDW EM
SSB 0
LB 1.00 Hz
GB 0
PC 1.40

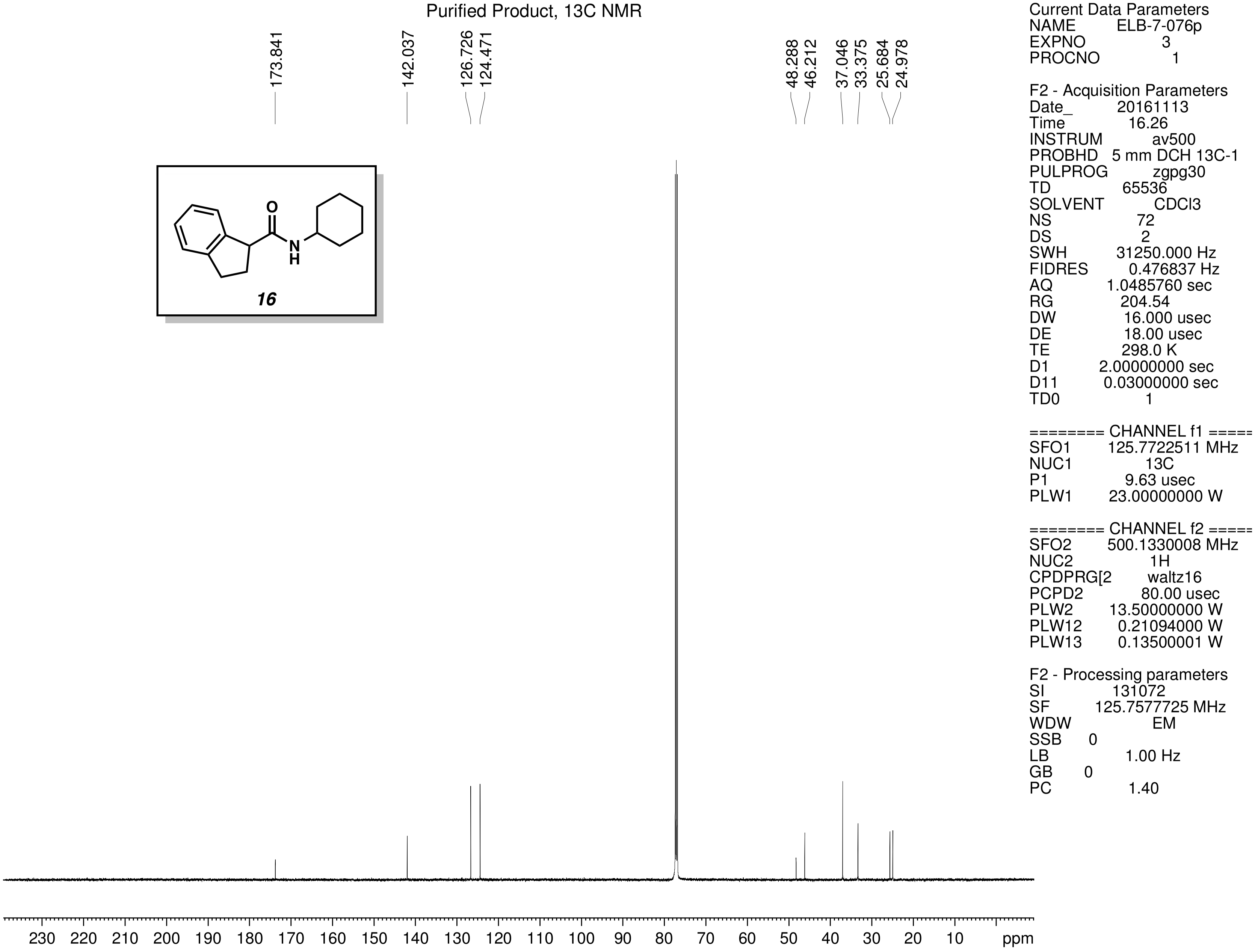
Purified Product, 13C NMR
173.841
142.037
126.726
124.471
48.288
46.212
37.046
33.375
25.684
24.978
O
N
H
16
230 220 210 200 190 180 170 160 150 140 130 120 110 100 90 80 70 60 50 40 30 20 10 ppm
Current Data Parameters
NAME ELB-7-076p
EXPNO 3
PROCNO 1
F2 - Acquisition Parameters
Date_ 20161113
Time 16.26
INSTRUM av500
PROBHD 5 mm DCH 13C-1
PULPROG zgpg30
TD 65536
SOLVENT CDCl3
NS 72
DS 2
SWH 31250.000 Hz
FIDRES 0.476837 Hz
AQ 1.0485760 sec
RG 204.54
DW 16.000 usec
DE 18.00 usec
TE 298.0 K
D1 2.00000000 sec
D11 0.03000000 sec
TD0 1
======== CHANNEL f1 =====
SFO1 125.7722511 MHz
NUC1 13C
P1 9.63 usec
PLW1 23.00000000 W
======== CHANNEL f2 =====
SFO2 500.1330008 MHz
NUC2 1H
CPDPRG[2 waltz16
PCPD2 80.00 usec
PLW2 13.50000000 W
PLW12 0.21094000 W
PLW13 0.13500001 W
F2 - Processing parameters
SI 131072
SF 125.7577725 MHz
WDW EM
SSB 0
LB 1.00 Hz
GB 0
PC 1.40

Purified Product, 13C NMR

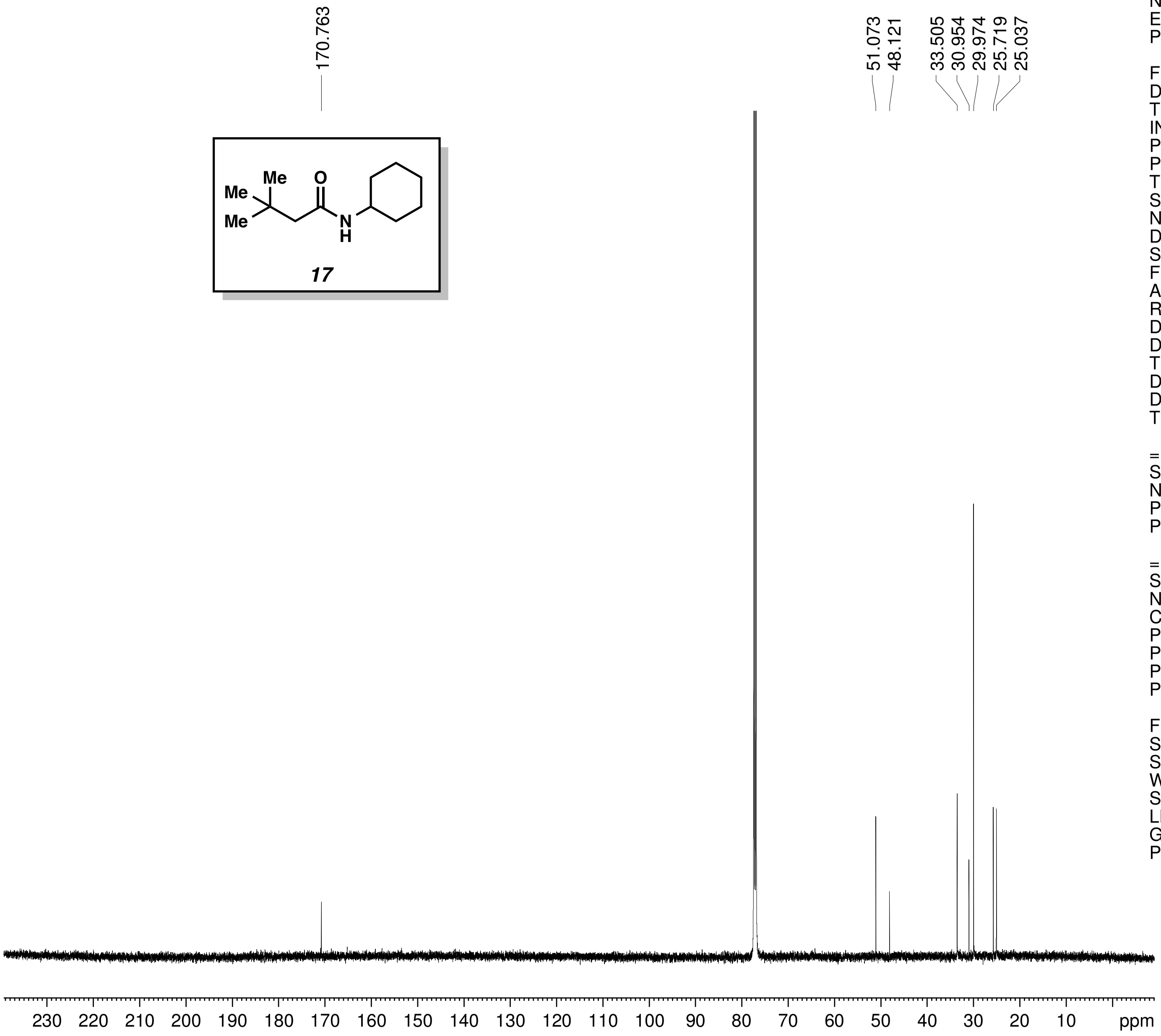

Current Data Parameters
NAME ELB-7-139p
EXPNO 4
PROCNO 1

F2 - Acquisition Parameters
Date_ 20161207
Time 11.48
INSTRUM av500
PROBHD 5 mm DCH 13C-1
PULPROG zgpg30
TD 65536
SOLVENT CDCl3
NS 108
DS 2
SWH 31250.000 Hz
FIDRES 0.476837 Hz
AQ 1.0485760 sec
RG 204.54
DW 16.000 usec
DE 18.00 usec
TE 298.0 K
D1 2.00000000 sec
D11 0.03000000 sec
TD0 1

======== CHANNEL f1 =====
SFO1 125.7722511 MHz
NUC1 13C
P1 9.63 usec
PLW1 23.00000000 W

======== CHANNEL f2 =====
SFO2 500.1330008 MHz
NUC2 1H
CPDPRG[2 waltz16
PCPD2 80.00 usec
PLW2 13.50000000 W
PLW12 0.21094000 W
PLW13 0.13500001 W

F2 - Processing parameters
SI 131072
SF 125.7577719 MHz
WDW EM
SSB 0
LB 1.00 Hz
GB 0
PC 1.40

Purified Product, 13C NMR

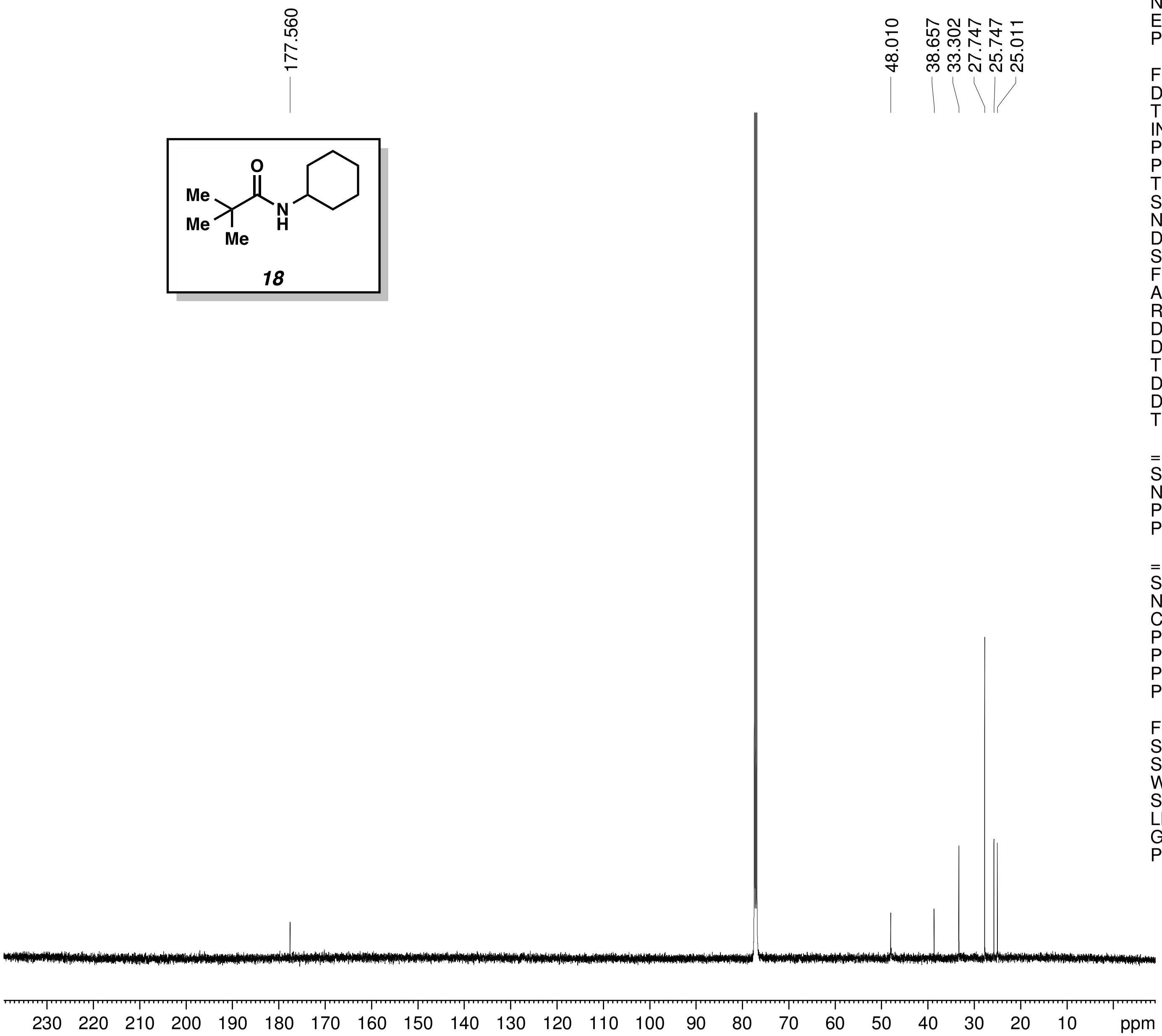

Current Data Parameters
NAME ELB-7-070p
EXPNO 6
PROCNO 1

F2 - Acquisition Parameters
Date_ 20161128
Time 15.13
INSTRUM av500
PROBHD 5 mm DCH 13C-1
PULPROG zgpg30
TD 65536
SOLVENT CDCl3
NS 108
DS 2
SWH 31250.000 Hz
FIDRES 0.476837 Hz
AQ 1.0485760 sec
RG 204.54
DW 16.000 usec
DE 18.00 usec
TE 298.0 K
D1 2.00000000 sec
D11 0.03000000 sec
TD0 1

======== CHANNEL f1 =====
SFO1 125.7722511 MHz
NUC1 13C
P1 9.63 usec
PLW1 23.00000000 W

======== CHANNEL f2 =====
SFO2 500.1330008 MHz
NUC2 1H
CPDPRG[2 waltz16
PCPD2 80.00 usec
PLW2 13.50000000 W
PLW12 0.21094000 W
PLW13 0.13500001 W

F2 - Processing parameters
SI 131072
SF 125.7577728 MHz
WDW EM
SSB 0
LB 1.00 Hz
GB 0
PC 1.40

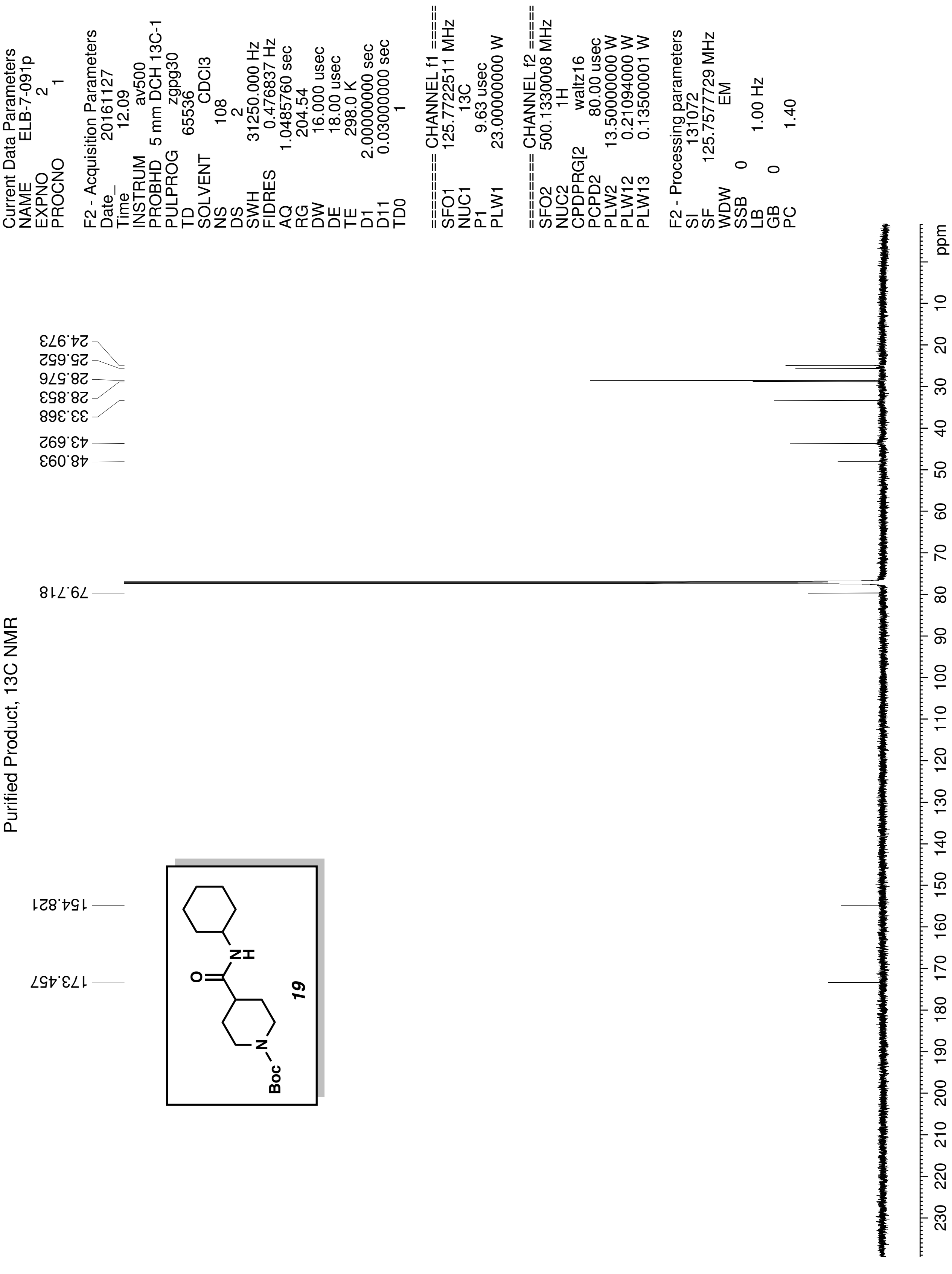
Purified Product, 13C NMR
173.457
154.821
79.718
48.093
43.692
33.368
28.853
28.576
25.652
24.973
Boc
N
O
H
19
Current Data Parameters
NAME ELB-7-091p
EXPNO 2
PROCNO 1
F2 - Acquisition Parameters
Date_ 20161127
Time 12.09
INSTRUM av500
PROBHD 5 mm DCH 13C-1
PULPROG zgpg30
TD 65536
SOLVENT CDCl3
NS 108
DS 2
SWH 31250.000 Hz
FIDRES 0.476837 Hz
AQ 1.0485760 sec
RG 204.54
DW 16.000 usec
DE 18.00 usec
TE 298.0 K
D1 2.00000000 sec
D11 0.03000000 sec
TD0 1
======== CHANNEL f1 ========
SFO1 125.7722511 MHz
NUC1 13C
P1 9.63 usec
PLW1 23.00000000 W
======== CHANNEL f2 ========
SFO2 500.1330008 MHz
NUC2 1H
CPDPRG[2 waltz16
PCPD2 80.00 usec
PLW2 13.50000000 W
PLW12 0.21094000 W
PLW13 0.13500001 W
F2 - Processing parameters
SI 131072
SF 125.7577729 MHz
WDW EM
SSB 0
LB 1.00 Hz
GB 0
PC 1.40
230 220 210 200 190 180 170 160 150 140 130 120 110 100 90 80 70 60 50 40 30 20 10 ppm

Purified Product, 13C NMR

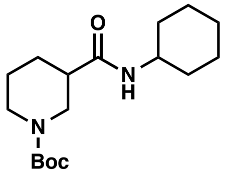

20

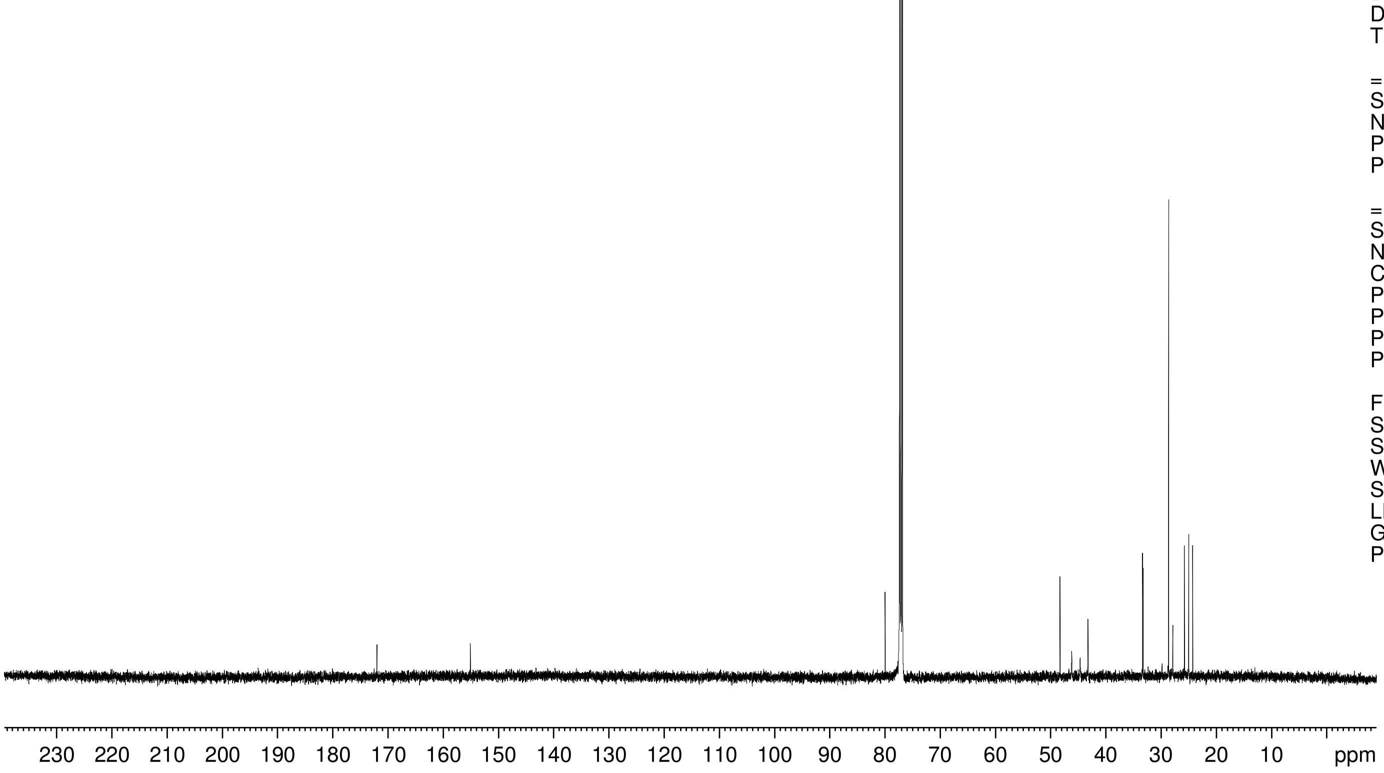

Current Data Parameters
NAME ELB-7-043p
EXPNO 20
PROCNO 1

F2 - Acquisition Parameters
Date_ 20170319
Time 14.28
INSTRUM av500
PROBHD 5 mm DCH 13C-1
PULPROG zgpg30
TD 65536
SOLVENT CDCl3
NS 123
DS 2
SWH 31250.000 Hz
FIDRES 0.476837 Hz
AQ 1.0485760 sec
RG 204.54
DW 16.000 usec
DE 18.00 usec
TE 333.0 K
D1 2.00000000 sec
D11 0.03000000 sec
TD0 1

======== CHANNEL f1 =====
SFO1 125.7722511 MHz
NUC1 13C
P1 9.63 usec
PLW1 23.00000000 W

======== CHANNEL f2 =====
SFO2 500.1330008 MHz
NUC2 1H
CPDPRG[2 waltz16
PCPD2 80.00 usec
PLW2 13.50000000 W
PLW12 0.21094000 W
PLW13 0.13500001 W

F2 - Processing parameters
SI 131072
SF 125.7577571 MHz
WDW EM
SSB 0
LB 1.00 Hz
GB 0
PC 1.40

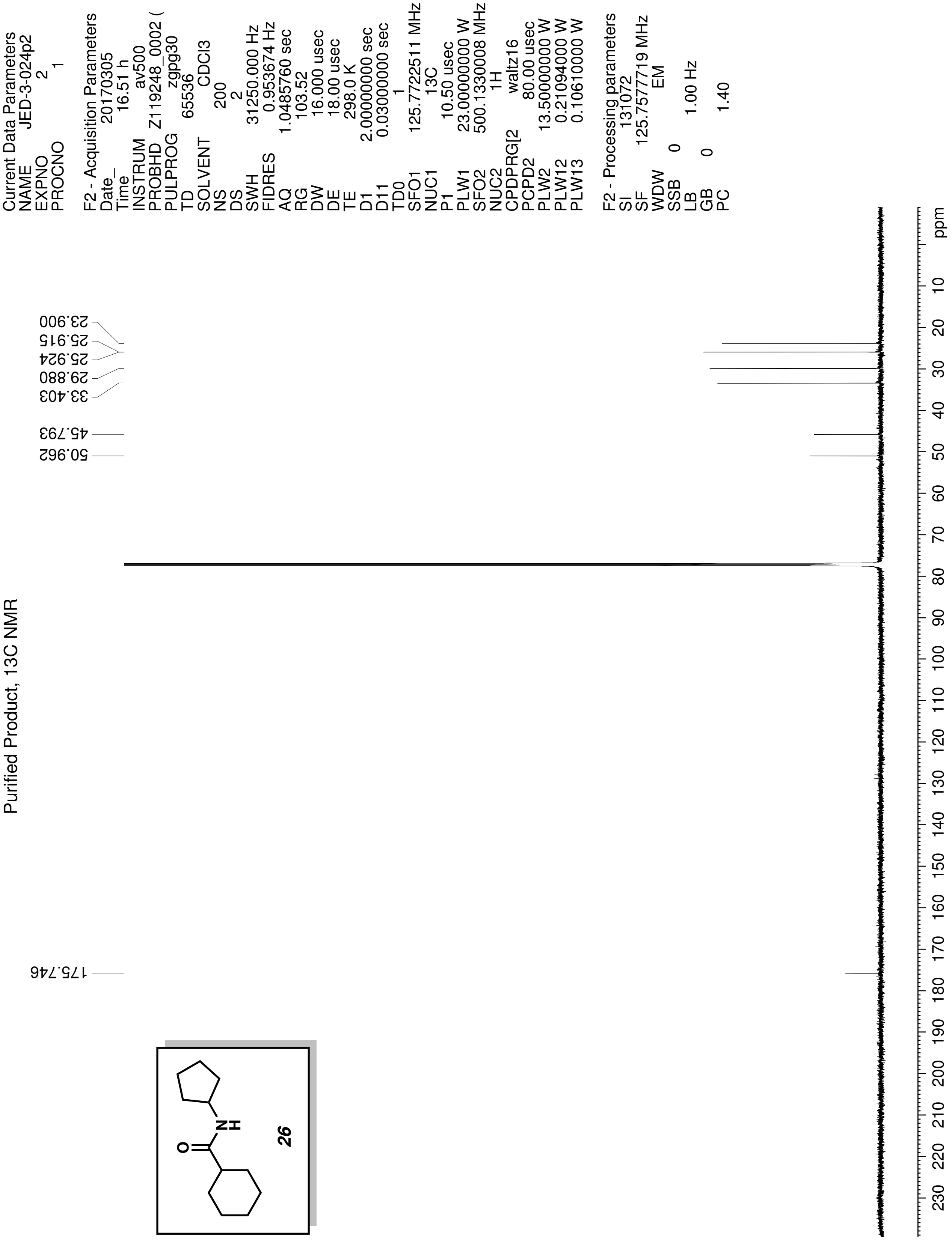
Purified Product, 13C NMR
26
O
N
H
175.746
50.962
45.793
33.403
29.880
25.924
25.915
23.900
230 220 210 200 190 180 170 160 150 140 130 120 110 100 90 80 70 60 50 40 30 20 10 ppm
Current Data Parameters
NAME JED-3-024p2
EXPNO 2
PROCNO 1
F2 - Acquisition Parameters
Date_ 20170305
Time 16.51 h
INSTRUM av500
PROBHD Z119248_0002 (
PULPROG zgpg30
TD 65536
SOLVENT CDCl3
NS 200
DS 2
SWH 31250.000 Hz
FIDRES 0.953674 Hz
AQ 1.0485760 sec
RG 103.52
DW 16.000 usec
DE 18.00 usec
TE 298.0 K
D1 2.00000000 sec
D11 0.03000000 sec
TD0 1
SFO1 125.7722511 MHz
NUC1 13C
P1 10.50 usec
PLW1 23.00000000 W
SFO2 500.1330008 MHz
NUC2 1H
CPDPRG[2 waltz16
PCPD2 80.00 usec
PLW2 13.50000000 W
PLW12 0.21094000 W
PLW13 0.10610000 W
F2 - Processing parameters
SI 131072
SF 125.7577719 MHz
WDW EM
SSB 0
LB 1.00 Hz
GB 0
PC 1.40

Purified Product, 13C NMR

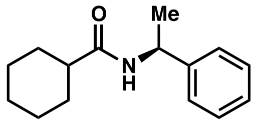

28

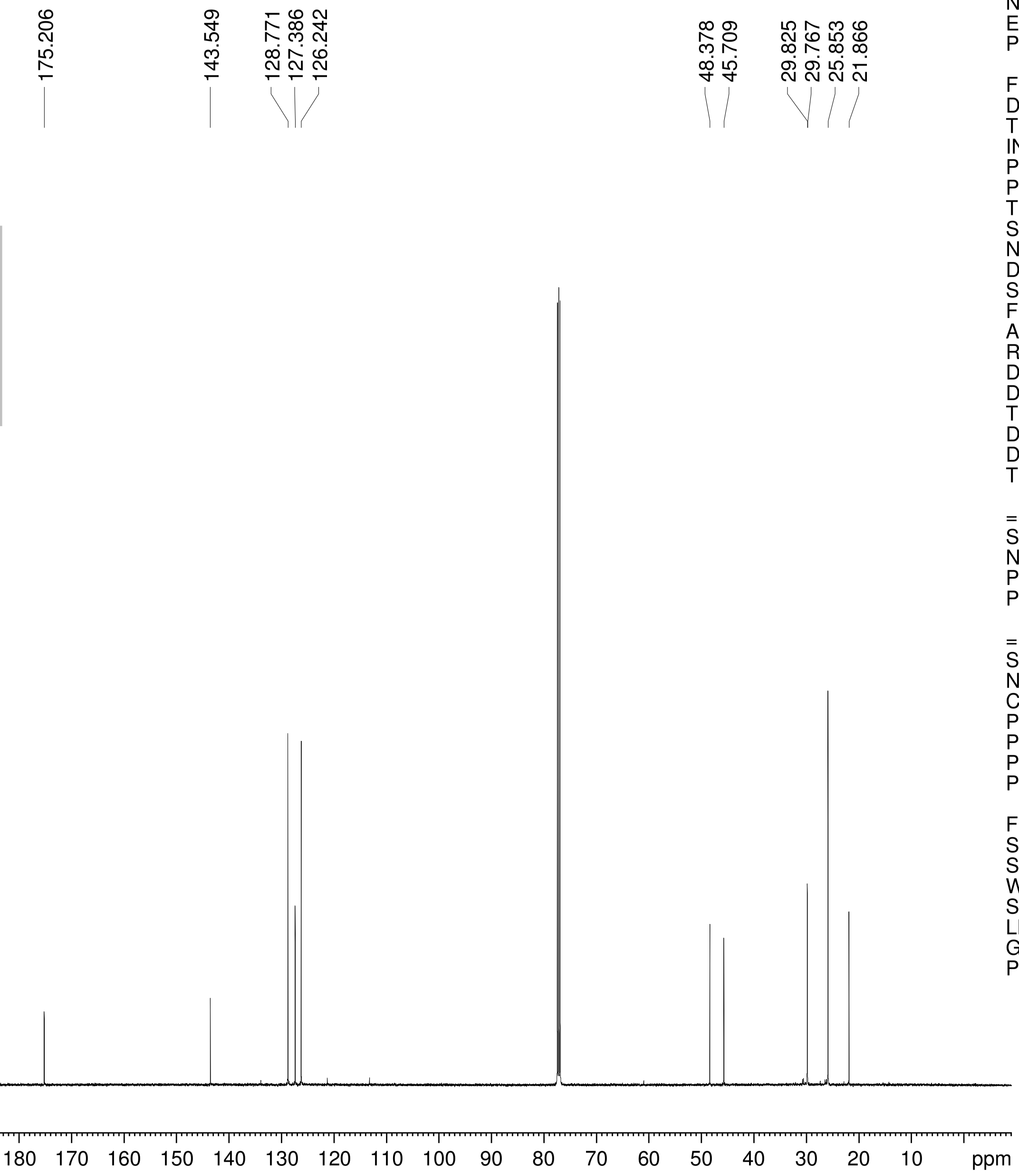

Current Data Parameters
NAME JED-3-064p
EXPNO 2
PROCNO 1

F2 - Acquisition Parameters
Date_ 20170116
Time 16.25
INSTRUM av500
PROBHD 5 mm DCH 13C-1
PULPROG zgpg30
TD 65536
SOLVENT CDCl3
NS 62
DS 2
SWH 31250.000 Hz
FIDRES 0.476837 Hz
AQ 1.0485760 sec
RG 204.54
DW 16.000 usec
DE 18.00 usec
TE 298.0 K
D1 2.00000000 sec
D11 0.03000000 sec
TD0 1

======== CHANNEL f1 =====
SFO1 125.7722511 MHz
NUC1 13C
P1 9.63 usec
PLW1 23.00000000 W

======== CHANNEL f2 =====
SFO2 500.1330008 MHz
NUC2 1H
CPDPRG[2 waltz16
PCPD2 80.00 usec
PLW2 13.50000000 W
PLW12 0.21094000 W
PLW13 0.13500001 W

F2 - Processing parameters
SI 131072
SF 125.7577745 MHz
WDW EM
SSB 0
LB 1.00 Hz
GB 0
PC 1.40

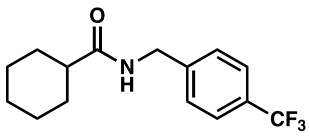

30

Purified Product, 13C NMR

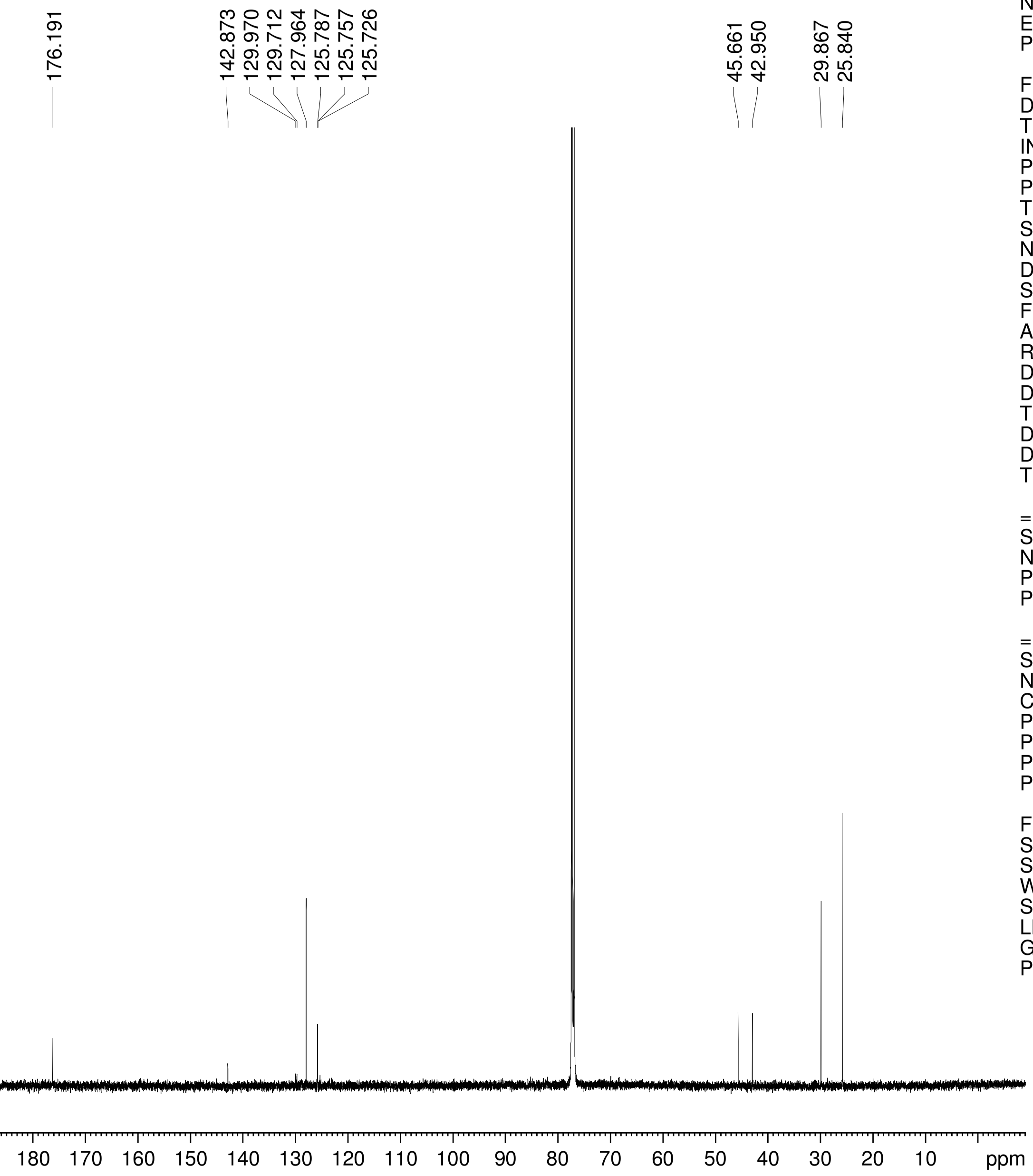

Current Data Parameters
NAME ELB-7-182p
EXPNO 2
PROCNO 1

F2 - Acquisition Parameters
Date_ 20170112
Time 12.19
INSTRUM av500
PROBHD 5 mm DCH 13C-1
PULPROG zgpg30
TD 65536
SOLVENT CDCl3
NS 108
DS 2
SWH 31250.000 Hz
FIDRES 0.476837 Hz
AQ 1.0485760 sec
RG 204.54
DW 16.000 usec
DE 18.00 usec
TE 298.0 K
D1 2.00000000 sec
D11 0.03000000 sec
TD0 1

======== CHANNEL f1 =====
SFO1 125.7722511 MHz
NUC1 13C
P1 9.63 usec
PLW1 23.00000000 W

======== CHANNEL f2 =====
SFO2 500.1330008 MHz
NUC2 1H
CPDPRG[2 waltz16
PCPD2 80.00 usec
PLW2 13.50000000 W
PLW12 0.21094000 W
PLW13 0.13500001 W

F2 - Processing parameters
SI 131072
SF 125.7577722 MHz
WDW EM
SSB 0
LB 1.00 Hz
GB 0
PC 1.40

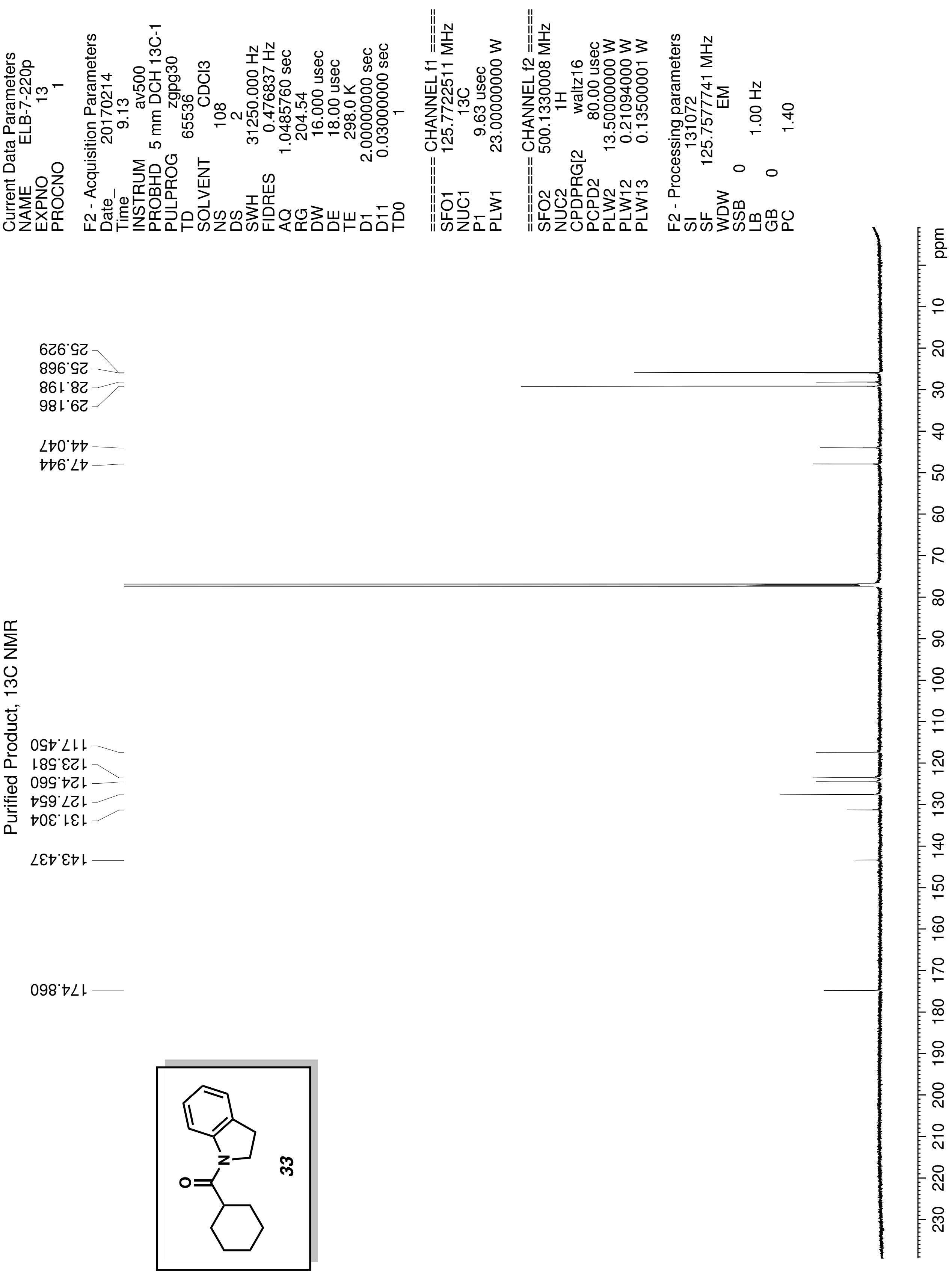
Purified Product, 13C NMR
174.860
143.437
131.304
127.654
124.560
123.581
117.450
47.944
44.047
29.186
28.198
25.968
25.929
33
ppm
Current Data Parameters
NAME ELB-7-220p
EXPNO 13
PROCNO 1
F2 - Acquisition Parameters
Date_ 20170214
Time 9.13
INSTRUM av500
PROBHD 5 mm DCH 13C-1
PULPROG zgpg30
TD 65536
SOLVENT CDCl3
NS 108
DS 2
SWH 31250.000 Hz
FIDRES 0.476837 Hz
AQ 1.0485760 sec
RG 204.54
DW 16.000 usec
DE 18.00 usec
TE 298.0 K
D1 2.00000000 sec
D11 0.03000000 sec
TD0 1
======== CHANNEL f1 ========
SFO1 125.7722511 MHz
NUC1 13C
P1 9.63 usec
PLW1 23.00000000 W
======== CHANNEL f2 ========
SFO2 500.1330008 MHz
NUC2 1H
CPDPRG[2 waltz16
PCPD2 80.00 usec
PLW2 13.50000000 W
PLW12 0.21094000 W
PLW13 0.13500001 W
F2 - Processing parameters
SI 131072
SF 125.7577741 MHz
WDW EM
SSB 0
LB 1.00 Hz
GB 0
PC 1.40

Purified Product, 13C NMR

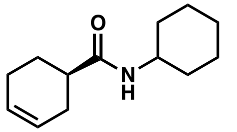

39

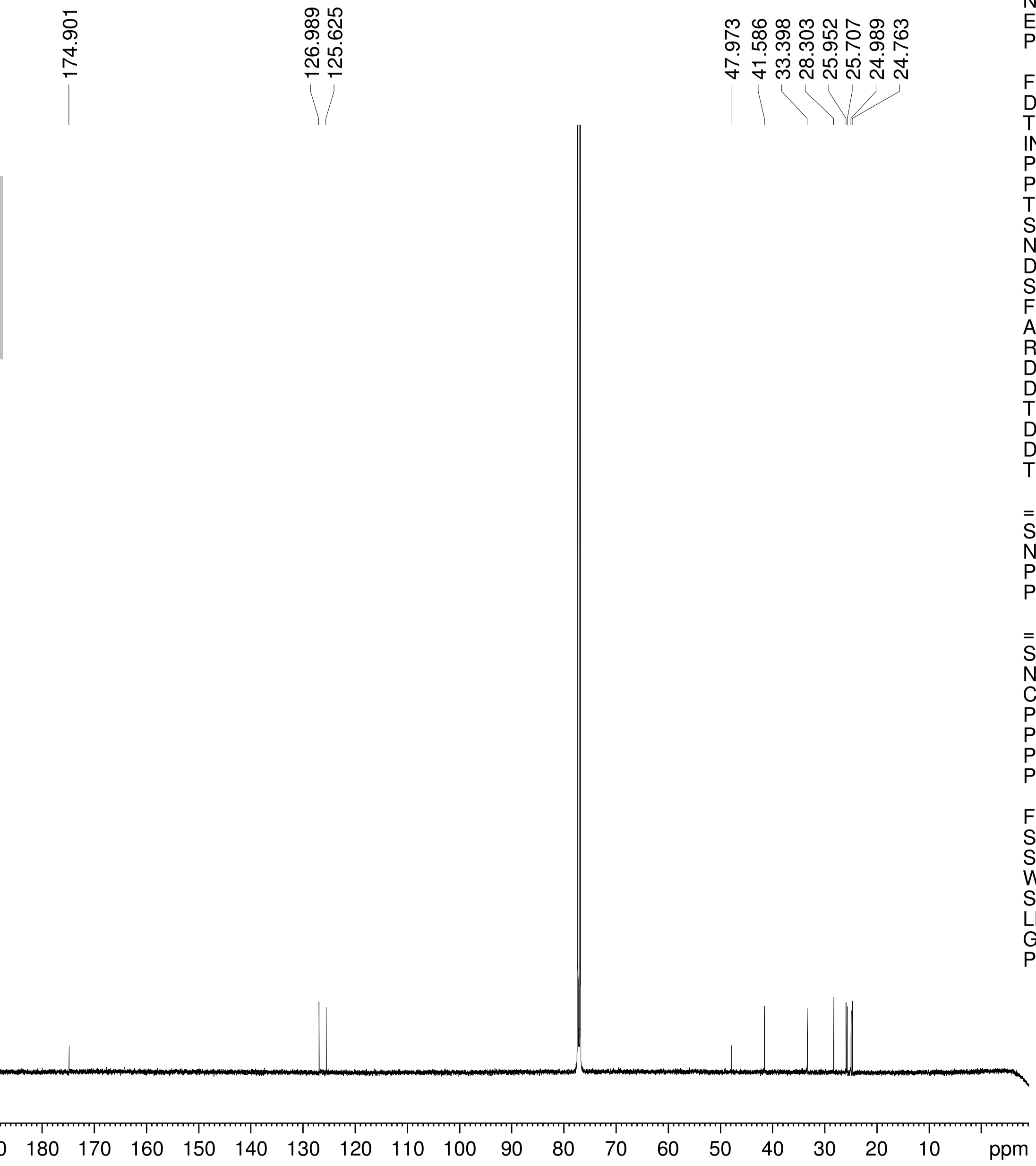

Current Data Parameters
NAME ELB-7-111p
EXPNO 4
PROCNO 1

F2 - Acquisition Parameters
Date_ 20161128
Time 13.14
INSTRUM av500
PROBHD 5 mm DCH 13C-1
PULPROG zgpg30
TD 65536
SOLVENT CDCl3
NS 108
DS 2
SWH 31250.000 Hz
FIDRES 0.476837 Hz
AQ 1.0485760 sec
RG 204.54
DW 16.000 usec
DE 18.00 usec
TE 298.0 K
D1 2.00000000 sec
D11 0.03000000 sec
TD0 1

======== CHANNEL f1 =====
SFO1 125.7722511 MHz
NUC1 13C
P1 9.63 usec
PLW1 23.00000000 W

======== CHANNEL f2 =====
SFO2 500.1330008 MHz
NUC2 1H
CPDPRG[2 waltz16
PCPD2 80.00 usec
PLW2 13.50000000 W
PLW12 0.21094000 W
PLW13 0.13500001 W

F2 - Processing parameters
SI 131072
SF 125.7577723 MHz
WDW EM
SSB 0
LB 1.00 Hz
GB 0
PC 1.40
